# Nine anthropometric insulin resistance indices predict mortality and life expectancy across glucose states

**DOI:** 10.1016/j.isci.2026.117400

**Published:** 2026-09-07

**Authors:** Yi Tang, Yameng Xu, Zilong Yue, Wencai Wang, Song Wen, Haiyang Wang, Lingyun Liu, Juncang Wu, Zhonghua Sun

**Affiliations:** 1 Department of Neurology, Anhui Medical University, Hefei 230032, China; 2 Department of Neurology, The Second People’s Hospital of Hefei, Hefei 230011, China; 3 Department of Epidemiology, School of Public Health, Nanjing Medical University, Nanjing 211166, China; 4 Department of General Surgery, Guoyang Branch of Anhui Provincial Hospital, Bozhou 233600, China; 5 The Second Affiliated Hospital of Harbin Medical University, Harbin 150086, China; 6 Department of Cardiology, Fuwai Hospital Chinese Academy of Medical Sciences and Peking Union Medical College, Beijing 100037, China; 7 College of Life Sciences, Anhui Normal University, Wuhu, Anhui 241000, China; 8 Department of Intensive Care Unit, The Second Affiliated Hospital of Nanjing Medical University, Nanjing 210011, China; 9 Medical School of Nanjing University, Nanjing 210093, China

**Keywords:** triglyceride-glucose index, TyG-ABSI, central obesity, insulin resistance, all-cause mortality, cardiovascular mortality, life expectancy, NHANES, CHARLS

## Abstract

We compared the impact of insulin resistance indicators (triglyceride-glucose index, TyG) combined with different obesity metrics on long-term survival rates across varying states of glucose metabolism. In 11,787 U.S. adults aged ≥45 years from NHANES (1999–2018; mortality follow-up through December 31, 2019) and 12,915 participants from CHARLS for external validation, we examined nine TyG-derived indices using glycemic-status-stratified Cox models and flexible dose-response analyses, and assessed their predictive utility and clinical net benefit. The TyG index combined with a body shape index (TyG-ABSI) showed relatively consistent associations with all-cause and cardiovascular mortality and comparatively favorable incremental predictive performance; external validation in CHARLS supported its association with all-cause mortality, although its standalone discriminatory ability was modest. Higher TyG-ABSI was also associated with shorter life expectancy and a larger estimated population-attributable fraction, supporting its potential role in risk stratification across glucose metabolism states.

## Highlights

• TyG-ABSI showed consistent mortality associations across glycemic states
• TyG-ABSI improved reclassification and net benefit over other TyG indices
• Higher TyG-ABSI was linked to shorter life expectancy and mortality burden
• CHARLS validation supported TyG-ABSI for all-cause mortality risk stratification

## Graphical abstract

**Figure:**
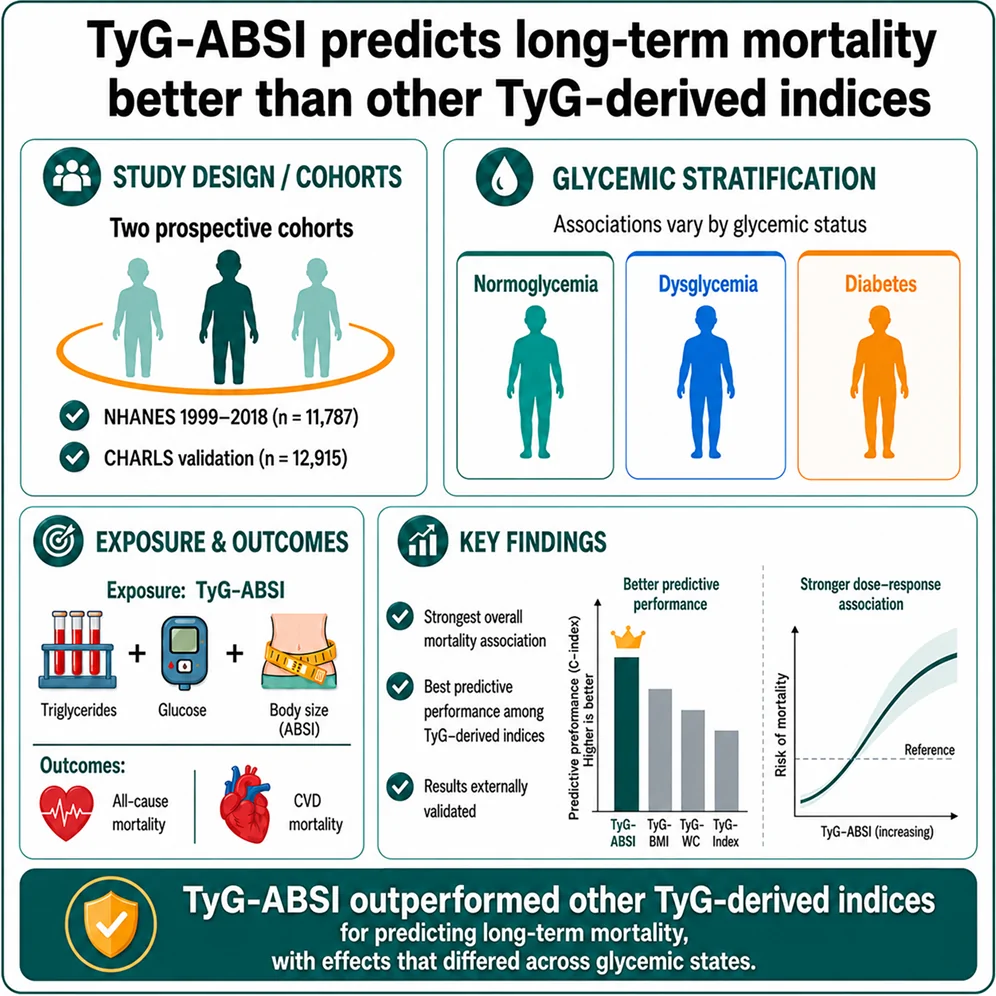

## Teaser

Health sciences

## Introduction

Insulin resistance is a key pathophysiological mechanism linking metabolic dysfunction to cardiovascular disease and premature mortality, particularly in middle-aged and elderly populations.^1^^,^^2^^,^^3^ Traditional assessment methods, particularly the hyperinsulinemic-euglycemic clamp and homeostatic model assessment of insulin resistance (HOMA-IR), face significant limitations in clinical practice due to high cost, technical complexity, and limited accessibility in resource-constrained settings.^4^^,^^5^ The triglyceride-glucose index (TyG) has emerged as a practical surrogate biomarker, as it can be obtained through routine laboratory testing alone. Large-scale validation studies demonstrated strong correlations with gold-standard clamp measurements (r = −0.68)^6^ and superior predictive performance compared to HOMA-IR.^7^^,^^8^^,^^9^ Recent evidence from the multinational PURE study (141,243 participants)^10^ and the ChinaHEART cohort (3.5 million participants)^11^ established robust associations between elevated TyG index and increased risks of cardiovascular events and mortality across diverse populations, with particularly pronounced effects in low- and middle-income countries where traditional risk assessment tools may be inaccessible.^12^^,^^13^ In addition, the physiological trajectory of aging is inextricably coupled with progressive decline in insulin sensitivity and cumulative metabolic burden, rendering middle-aged and older adults disproportionately susceptible to atherosclerotic cardiovascular disease.^14^ In the context of accelerating global population aging, validating the TyG index in this specific demographic is imperative to refine risk stratification and optimize primary prevention strategies for the population bearing the highest burden of mortality.^15^

Building upon the foundation of TyG as an insulin resistance marker, researchers have developed composite indices integrating anthropometric parameters to capture both metabolic dysfunction and adipose tissue distribution abnormalities.^16^ These composite indices include the TyG index combined with body mass index (TyG-BMI), waist circumference (TyG-WC), waist-to-height ratio (TyG-WHtR), weight-adjusted waist index (TyG-WWI), a body shape index (TyG-ABSI), conicity index (TyG-CI), body roundness index (TyG-BRI), and relative fat mass (TyG-RFM), and have demonstrated superior predictive performance for cardiovascular outcomes over the TyG index alone in multiple cohort studies.^17^^,^^18^ For instance, analysis from the UK Biobank revealed that TyG-WC demonstrated a hazard ratio of 1.53 for myocardial infarction incidence, significantly higher than that observed with TyG alone (HR = 1.36).^19^ Similarly, National Health and Nutrition Examination Survey (NHANES) investigations showed TyG-WHtR outperformed standalone TyG for mortality prediction in U.S. populations.^20^ However, critical knowledge gaps persist regarding the comparative performance of multiple TyG-derived indices within the same population, particularly across different glycemic states. Existing studies predominantly focus on cardiovascular disease incidence rather than mortality outcomes, and systematic comparisons stratified by glucose metabolism status (normal glucose tolerance, impaired glucose metabolism, and diabetes) remain notably scarce.^21^ In addition, understanding the pathways linking the TyG index to mortality is equally important; key mechanisms to investigate include systemic inflammation, endothelial dysfunction, and other potential mediators.

Therefore, we conducted a large-scale study using NHANES data, with external validation in the China Health and Retirement Longitudinal Study (CHARLS) cohort, to comprehensively evaluate the comparative prognostic utility of nine insulin resistance surrogates—specifically TyG, TyG-BMI, TyG-WC, TyG-WHtR, TyG-WWI, TyG-ABSI, TyG-CI, TyG-BRI, and TyG-RFM—in predicting all-cause and cardiovascular mortality among middle-aged and older adults. By integrating analyses of dose-response patterns, inflammatory mediation pathways, and life expectancy projections across the glycemic spectrum, we aimed to compare the relative prognostic performance of these TyG-derived indices and to evaluate their potential value for mortality risk stratification across the glycemic spectrum.

## Results

### Baseline characteristics

**Figure 1:**
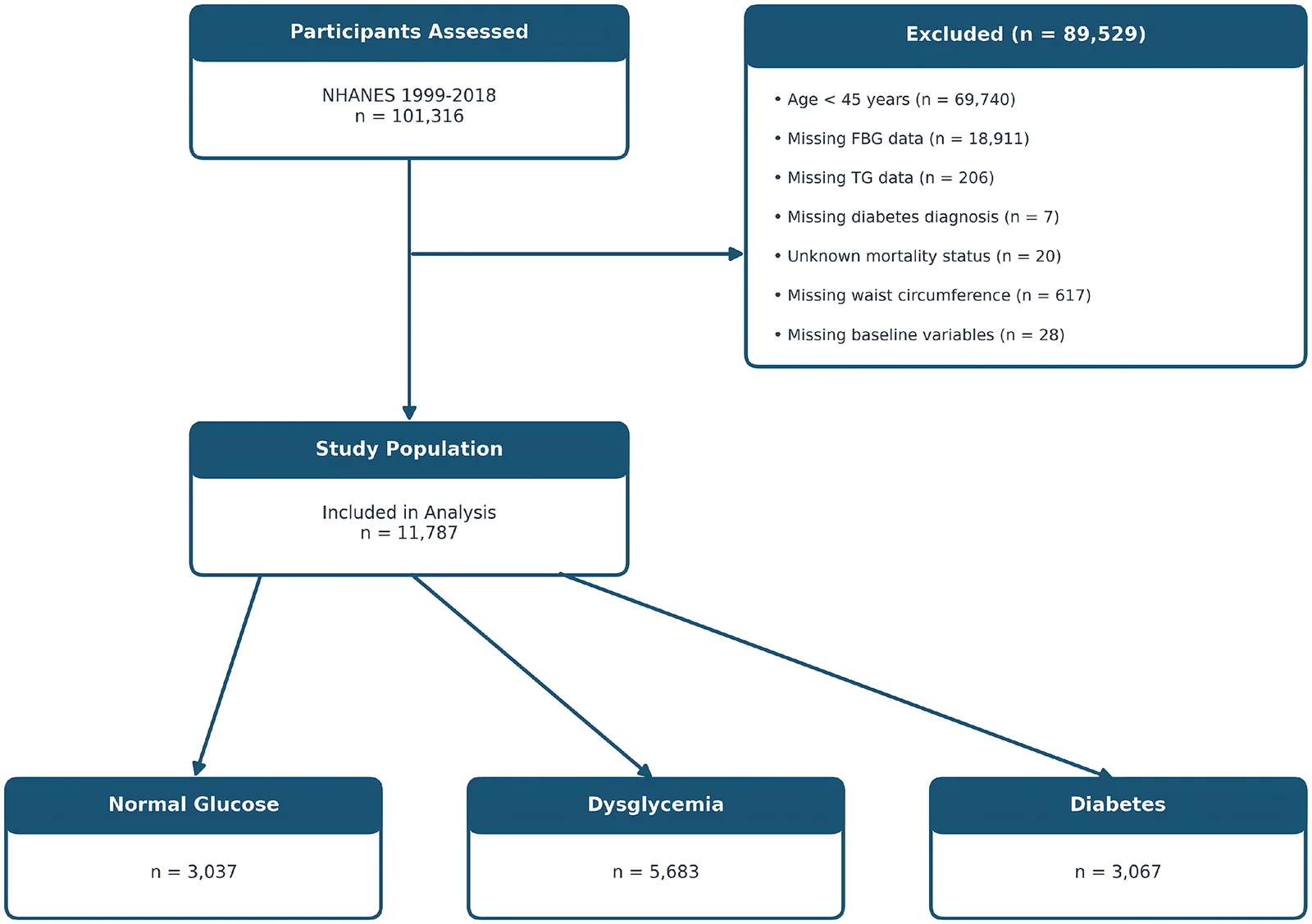
Flow diagram of participant selection from the NHANES 1999–2018 NHANES, National Health and Nutrition Examination Survey.

This study (Table 1; Figure 1) included 11,787 participants (49.43% male; mean age 62.55 years). Compared with survivors, participants in the mortality group were older, more likely to be male, and had a higher prevalence of hypertension, diabetes, and cardiovascular comorbidities. Among TyG-derived indices, TyG-ABSI, TyG-WWI, and TyG-CI were significantly elevated in the mortality group (all *p* < 0.001). During follow-up, 2,604 deaths occurred (22.09%), of which 830 (31.87%) were attributable to cardiovascular disease. Additionally, we compared participants included and excluded from the study to assess potential selection bias (Table S1). Included and excluded participants differed in fasting glucose, triglycerides, TyG index, and TyG-BMI, indicating that complete-case restriction may have affected the distribution of TyG-related exposures. Moreover, the CHARLS validation cohort included 12,915 participants, with a mean age of 57.61 years and 7,019 women (54.35%); 5,002 participants (38.74%) had normal glucose metabolism, 5,706 (44.17%) had dysglycemia, and 2,207 (17.09%) had diabetes. During follow-up, 1,332 deaths occurred (10.31%), and the mortality group was older, more often male, and had a higher prevalence of diabetes; TyG-ABSI, TyG-WWI, and TyG-CI were similarly elevated in the mortality group (Table S2).

**Table 1:** Baseline characteristics of participants in the general population stratified by all-cause mortality

Characteristics	Total	Alive	Deceased	*p*-value
N	11787	9183	2604	–
Age (years)	62.55 ± 11.02	60.096 ± 9.984	71.189 ± 10.122	<0.001
PIR	2.63 ± 1.61	2.742 ± 1.640	2.235 ± 1.431	<0.001
Waist (cm)	101.20 ± 14.98	101.084 ± 14.947	101.599 ± 15.072	0.121
Height (cm)	166.36 ± 10.11	166.375 ± 10.107	166.301 ± 10.120	0.740
Weight (kg)	80.88 ± 19.75	81.554 ± 19.747	78.502 ± 19.570	<0.001
Neutrophil count (×10^9^/L)	3.98 ± 1.75	3.863 ± 1.518	4.407 ± 2.341	<0.001
Monocyte count (×10^9^/L)	0.54 ± 0.21	0.529 ± 0.179	0.599 ± 0.303	<0.001
Lymphocyte count (×10^9^/L)	1.99 ± 1.58	1.992 ± 0.898	1.978 ± 2.919	0.685
White blood cell (×10^9^/L)	6.77 ± 2.61	6.634 ± 2.003	7.253 ± 4.059	<0.001
Platelet count (×10^9^/L)	241.62 ± 67.10	242.275 ± 64.527	239.318 ± 75.448	0.047
SII	548.70 ± 448.17	515.246 ± 305.949	666.688 ± 749.257	<0.001
SIRI	1.26 ± 0.97	1.147 ± 0.786	1.649 ± 1.357	<0.001
AISI	308.74 ± 335.75	280.738 ± 221.504	407.496 ± 569.937	<0.001
Permth-INT (month)	101.52 ± 60.59	105.690 ± 61.245	86.827 ± 55.795	<0.001
HbA1c (%)	5.98 ± 1.17	5.950 ± 1.122	6.107 ± 1.329	<0.001
Fasting blood glucose (mg/dL)	115.32 ± 40.08	113.727 ± 37.181	120.932 ± 48.542	<0.001
Triglycerides (mg/dL)	138.14 ± 110.66	136.948 ± 112.202	142.354 ± 104.944	0.028
TyG	8.78 ± 0.65	8.757 ± 0.649	8.851 ± 0.670	<0.001
TyG-BMI	256.71 ± 62.66	258.313 ± 63.075	251.063 ± 60.840	<0.001
TyG-WC	891.13 ± 163.66	888.085 ± 163.241	901.851 ± 164.734	<0.001
TyG-WHTR	5.37 ± 0.98	5.348 ± 0.984	5.430 ± 0.974	<0.001
TyG-WWI	99.48 ± 11.30	98.700 ± 11.195	102.226 ± 11.227	<0.001
TyG-ABSI	0.73 ± 0.07	0.726 ± 0.073	0.756 ± 0.073	<0.001
TyG-CI	11.76 ± 1.30	11.665 ± 1.286	12.080 ± 1.292	<0.001
TyG-BRI	51.30 ± 20.49	51.063 ± 20.573	52.129 ± 20.181	0.019
TyG-RFM	321.56 ± 78.66	322.619 ± 78.791	317.846 ± 78.077	0.006
Gender				<0.001
Male	5,826 (49.4%)	4346 (47.327%)	1480 (56.836%)	
Female	5,961 (50.6%)	4837 (52.673%)	1124 (43.164%)	
Race				<0.001
Mexican American	1,843 (15.6%)	1491 (16.237%)	352 (13.518%)	
Other Hispanic	1,064 (9.0%)	947 (10.313%)	117 (4.493%)	
Non-Hispanic White	5,563 (47.2%)	3978 (43.319%)	1585 (60.868%)	
Non-Hispanic Black	2,301 (19.5%)	1846 (20.102%)	455 (17.473%)	
Other Race	1,016 (8.6%)	921 (10.029%)	95 (3.648%)	
Education				<0.001
Less than 9th grade	1,803 (15.3%)	1248 (13.590%)	555 (21.313%)	
9-11th grade	1,736 (14.7%)	1268 (13.808%)	468 (17.972%)	
High school graduate/GED	2,722 (23.1%)	2061 (22.444%)	661 (25.384%)	
Some college or AA degree	3,047 (25.9%)	2495 (27.170%)	552 (21.198%)	
College graduate or above	2,479 (21.0%)	2111 (22.988%)	368 (14.132%)	
Marital status				<0.001
Married/living with partner	7,397 (62.8%)	5999 (65.327%)	1398 (53.687%)	
Others	4,390 (37.2%)	3184 (34.673%)	1206 (46.313%)	
Smoke				<0.001
Yes	6008 (50.971%)	4444 (48.394%)	1564 (60.061%)	
No	5779 (49.029%)	4739 (51.606%)	1040 (39.939%)	
Alcohol Use				<0.001
Yes	1970 (16.713%)	1436 (15.638%)	534 (20.507%)	
No	9817 (83.287%)	7747 (84.362%)	2070 (79.493%)	
Antihypertensive drug use				<0.001
Yes	9874 (83.770%)	7535 (82.054%)	2339 (89.823%)	
No	1913 (16.230%)	1648 (17.946%)	265 (10.177%)	
Hypertension				<0.001
No	4756 (40.350%)	4049 (44.092%)	707 (27.151%)	
Yes	7031 (59.650%)	5134 (55.908%)	1897 (72.849%)	
Heart failure				<0.001
No	11184 (94.884%)	8890 (96.809%)	2294 (88.095%)	
Yes	603 (5.116%)	293 (3.191%)	310 (11.905%)	
Coronary heart disease				<0.001
No	10966 (93.035%)	8712 (94.871%)	2254 (86.559%)	
Yes	821 (6.965%)	471 (5.129%)	350 (13.441%)	
Angina				<0.001
No	11256 (95.495%)	8886 (96.766%)	2370 (91.014%)	
Yes	531 (4.505%)	297 (3.234%)	234 (8.986%)	
Heart attack				<0.001
No	10952 (92.916%)	8730 (95.067%)	2222 (85.330%)	
Yes	835 (7.084%)	453 (4.933%)	382 (14.670%)	
Stroke				<0.001
No	11089 (94.078%)	8791 (95.731%)	2298 (88.249%)	
Yes	698 (5.922%)	392 (4.269%)	306 (11.751%)	
Cancer				<0.001
No	10131 (85.951%)	8100 (88.206%)	2031 (77.995%)	
Yes	1656 (14.049%)	1083 (11.794%)	573 (22.005%)	
Failing kidney				<0.001
Yes	530 (4.496%)	322 (3.506%)	208 (7.988%)	
No	11257 (95.504%)	8861 (96.494%)	2396 (92.012%)	
CVD-death				<0.001
No	10957 (92.958%)	9183 (100.000%)	1774 (68.126%)	
Yes	830 (7.042%)	0 (0.000%)	830 (31.874%)	
State of glucose metabolism				<0.001
Normal glucose metabolism	3,037 (25.8%)	2419 (26.342%)	618 (23.733%)	
Diabetes	3,067 (26.0%)	2214 (24.110%)	853 (32.757%)	
Dysglycemia	5,683 (48.2%)	4550 (49.548%)	1133 (43.510%)	

CVD, cardiovascular disease; PIR, poverty-income ratio; TyG, triglyceride-glucose index; BMI, body mass index; WC, waist circumference; WHtR, waist-to-height ratio; WWI, weight-adjusted waist index; ABSI, a body shape index; CI, conicity index; BRI, body roundness index; RFM, relative fat mass; SII, systemic immune-inflammation index; SIRI, systemic inflammatory response index; AISI, aggregate index of systemic inflammation; HbA1c, glycated hemoglobin; GED, General Educational Development; AA, associate degree.

### Incidence rates of cardiovascular and all-cause mortality

During follow-up, 2,604 all-cause deaths (22.09%; 26.11 per 1,000 person-years) and 830 cardiovascular deaths (7.04%; 8.32 per 1,000 person-years) occurred. Both all-cause and cardiovascular mortality rates increased significantly across TyG-ABSI quartiles (*P* for trend <0.001). The diabetes group exhibited the highest mortality burden (37.81 and 13.16 per 1,000 person-years for all-cause and cardiovascular mortality, respectively), followed by the dysglycemia and normoglycemia groups. Notably, within each glycemic subgroup, mortality rates were consistently lowest in the lowest TyG-ABSI quartile and highest in the top quartile (all *P* for trend <0.001), indicating a robust positive association regardless of glucose metabolic status (Table 2; detailed stratified analyses are provided in the supplemental information).

**Table 2:** Burden of all-cause and cardiovascular mortality stratified by glycemic status

	Participants	Cases	Incidence rate (%) (95% CI)	Per 1,000 person-years (95% CI)	P For trend (proportion)	P For trend (per 1,000 PY)
**All-cause Mortality**						

Total						
Overall	11787	2604	22.09 (21.35, 22.85)	26.11 (25.12, 27.14)	–	–
Q1	2947	376	12.76 (11.57, 14.02)	13.92 (12.55, 15.40)	–	–
Q2	2947	601	20.39 (18.95, 21.89)	23.69 (21.83, 25.66)	–	–
Q3	2946	702	23.83 (22.30, 25.41)	28.72 (26.63, 30.92)	–	–
Q4	2947	925	31.39 (29.71, 33.10)	40.41 (37.84, 43.10)	–	–
∗P for trend∗					<0.001	<0.001
Normal						
Overall	3037	618	20.35 (18.93, 21.83)	20.57 (18.98, 22.26)	–	–
Q1	1263	166	13.14 (11.33, 15.13)	12.96 (11.07, 15.09)	–	–
Q2	883	194	21.97 (19.28, 24.85)	22.13 (19.13, 25.47)	–	–
Q3	622	161	25.88 (22.48, 29.52)	26.82 (22.84, 31.30)	–	–
Q4	269	97	36.06 (30.32, 42.11)	39.38 (31.94, 48.05)	–	–
∗P for trend∗					<0.001	<0.001
Dysglycemia						
Overall	5683	1133	19.94 (18.90, 21.00)	24.04 (22.66, 25.49)	–	–
Q1	1388	163	11.74 (10.10, 13.55)	13.84 (11.80, 16.13)	–	–
Q2	1575	292	18.54 (16.65, 20.55)	22.32 (19.83, 25.03)	–	–
Q3	1543	329	21.32 (19.30, 23.45)	25.79 (23.08, 28.73)	–	–
Q4	1177	349	29.65 (27.05, 32.35)	36.72 (32.97, 40.78)	–	–
∗P for trend∗					<0.001	<0.001
Diabetes						
Overall	3067	853	27.81 (26.23, 29.43)	37.81 (35.31, 40.43)	–	–
Q1	296	47	15.88 (11.91, 20.55)	19.38 (14.24, 25.77)	–	–
Q2	489	115	23.52 (19.83, 27.53)	32.66 (26.96, 39.20)	–	–
Q3	781	212	27.14 (24.05, 30.41)	37.27 (32.42, 42.64)	–	–
Q4	1501	479	31.91 (29.56, 34.34)	43.84 (40.00, 47.95)	–	–
∗P for trend∗					<0.001	<0.001

**CVD-cause Mortality**						

Total						
Overall	11787	830	7.04 (6.59, 7.52)	8.32 (7.77, 8.91)	–	–
Q1	2947	117	3.97 (3.29, 4.74)	4.33 (3.58, 5.19)	–	–
Q2	2947	192	6.52 (5.65, 7.47)	7.57 (6.54, 8.72)	–	–
Q3	2946	216	7.33 (6.42, 8.33)	8.84 (7.70, 10.10)	–	–
Q4	2947	305	10.35 (9.27, 11.51)	13.32 (11.87, 14.91)	–	–
∗P for trend∗					<0.001	<0.001
Normal						
Overall	3037	164	5.40 (4.62, 6.26)	5.46 (4.66, 6.36)	–	–
Q1	1263	52	4.12 (3.09, 5.36)	4.06 (3.03, 5.32)	–	–
Q2	883	46	5.21 (3.84, 6.89)	5.25 (3.84, 7.00)	–	–
Q3	622	41	6.59 (4.77, 8.84)	6.83 (4.90, 9.27)	–	–
Q4	269	25	9.29 (6.10, 13.41)	10.15 (6.57, 14.98)	–	–
∗P for trend∗					<0.001	<0.001
Dysglycemia						
Overall	5683	369	6.49 (5.87, 7.17)	7.83 (7.05, 8.67)	–	–
Q1	1388	44	3.17 (2.31, 4.23)	3.74 (2.71, 5.01)	–	–
Q2	1575	104	6.60 (5.43, 7.94)	7.95 (6.50, 9.63)	–	–
Q3	1543	105	6.80 (5.60, 8.18)	8.23 (6.73, 9.96)	–	–
Q4	1177	116	9.86 (8.21, 11.70)	12.20 (10.09, 14.64)	–	–
∗P for trend∗					<0.001	<0.001
Diabetes						
Overall	3067	297	9.68 (8.66, 10.79)	13.16 (11.71, 14.75)	–	–
Q1	296	21	7.09 (4.45, 10.64)	8.66 (5.36, 13.23)	–	–
Q2	489	42	8.59 (6.26, 11.43)	11.93 (8.60, 16.12)	–	–
Q3	781	70	8.96 (7.05, 11.19)	12.31 (9.59, 15.55)	–	–
Q4	1501	164	10.93 (9.39, 12.61)	15.01 (12.80, 17.49)	–	–
∗P for trend∗					0.017	0.009

TyG-ABSI, triglyceride-glucose index combined with a body shape index; CI, confidence interval; PY, person-years.

### Associations between TyG-ABSI and mortality by glucose metabolic status

In the fully adjusted model (Model 2), TyG-ABSI demonstrated the strongest and most consistent associations with mortality among all indices examined (Table 3). Each SD increase in TyG-ABSI was associated with an 18.8% higher risk of all-cause mortality (HR = 1.188; 95% CI, 1.141–1.237; *p* < 0.001) and a 22.6% higher risk of cardiovascular mortality (HR = 1.226; 95% CI, 1.142–1.317; *p* < 0.001). Categorical analyses revealed clear dose-response relationships: compared with the lowest tertile, participants in the highest tertile had substantially elevated risks of both all-cause mortality (HR = 1.481; 95% CI, 1.328–1.651; *p* < 0.001) and cardiovascular mortality (HR = 1.596; 95% CI, 1.308–1.946; *p* < 0.001), with intermediate risks observed in the middle tertile (all-cause: HR = 1.229; cardiovascular: HR = 1.442; both *p* < 0.001). In contrast, the original TyG index was not associated with all-cause mortality in either continuous (HR = 1.031; *p* = 0.135) or categorical analyses (T3 vs. T1: HR = 0.987; *p* = 0.802), and showed only a modest continuous association with cardiovascular mortality (HR = 1.078; *p* = 0.036) that was attenuated in tertile comparisons (T3 vs. T1: HR = 1.098; *p* = 0.300).

**Table 3:** Association between TyG and its derivative indices with all-cause mortality and cardiovascular mortality in the general population and stratified by glucose metabolism status

	Crude model	Crude model	Model 1	Model 1	Model 2	Model 2
	HR (95%CI)	*p*-value	HR (95%CI)	*p*-value	HR (95%CI)	*p*-value
**All-cause mortality**						

Total						
TyG index (Per SD increase)	1.131 (1.090–1.173)	<0.001	1.082 (1.040–1.126)	<0.001	1.031 (0.991–1.073)	0.135
TyG-BMI (Per SD increase)	0.917 (0.880–0.956)	<0.001	0.923 (0.884–0.964)	<0.001	0.855 (0.818–0.893)	<0.001
TyG-WC (per SD increase)	1.126 (1.083–1.170)	<0.001	1.013 (0.973–1.056)	0.528	0.935 (0.896–0.976)	0.002
TyG-WHtR (per SD increase)	1.134 (1.091–1.178)	<0.001	1.048 (1.006–1.093)	0.025	0.967 (0.927–1.009)	0.126
TyG-WWI (per SD increase)	1.369 (1.320–1.420)	<0.001	1.216 (1.167–1.266)	<0.001	1.139 (1.092–1.187)	<0.001
TyG-ABSI (per SD increase)	1.459 (1.408–1.511)	<0.001	1.250 (1.202–1.301)	<0.001	1.188 (1.141–1.237)	<0.001
TyG-BRI (per SD increase)	1.112 (1.071–1.156)	<0.001	1.037 (0.995–1.081)	0.088	0.959 (0.918–1.001)	0.055
TyG-CI (per SD increase)	1.372 (1.323–1.424)	<0.001	1.179 (1.132–1.227)	<0.001	1.104 (1.059–1.151)	<0.001
TyG-RFM (per SD increase)	0.962 (0.925–1.000)	0.051	1.061 (0.999–1.126)	0.056	0.944 (0.888–1.005)	0.070
TyG index tertile groups						
T1	1.00 (Ref)	–	1.00 (Ref)	–	1.00 (Ref)	–
T2	1.139 (1.034–1.255)	0.008	1.032 (0.936–1.138)	0.527	0.978 (0.886–1.079)	0.652
T3	1.272 (1.157–1.399)	<0.001	1.102 (0.999–1.214)	0.051	0.987 (0.895–1.090)	0.802
TyG-BMI tertile groups						
T1	1.00 (Ref)	–	1.00 (Ref)	–	1.00 (Ref)	–
T2	0.898 (0.818–0.985)	0.022	0.874 (0.797–0.959)	0.005	0.800 (0.728–0.878)	<0.001
T3	0.838 (0.763–0.921)	<0.001	0.852 (0.775–0.937)	<0.001	0.728 (0.661–0.803)	<0.001
TyG-WC tertile groups						
T1	1.00 (Ref)	–	1.00 (Ref)	–	1.00 (Ref)	–
T2	1.127 (1.023–1.241)	0.015	0.931 (0.844–1.026)	0.150	0.864 (0.783–0.954)	0.004
T3	1.295 (1.178–1.424)	<0.001	1.011 (0.918–1.114)	0.821	0.859 (0.778–0.949)	0.003
TyG-WHtR tertile groups						
T1	1.00 (Ref)	–	1.00 (Ref)	–	1.00 (Ref)	–
T2	1.121 (1.018–1.234)	0.020	0.998 (0.905–1.100)	0.964	0.908 (0.823–1.001)	0.053
T3	1.319 (1.200–1.450)	<0.001	1.101 (1.000–1.213)	0.051	0.936 (0.847–1.034)	0.192
TyG-WWI tertile groups						
T1	1.00 (Ref)	–	1.00 (Ref)	–	1.00 (Ref)	–
T2	1.520 (1.372–1.685)	<0.001	1.238 (1.115–1.375)	<0.001	1.170 (1.053–1.300)	0.003
T3	2.141 (1.940–2.363)	<0.001	1.526 (1.376–1.693)	<0.001	1.336 (1.202–1.486)	<0.001
TyG-ABSI tertile groups						
T1	1.00 (Ref)	–	1.00 (Ref)	–	1.00 (Ref)	–
T2	1.698 (1.526–1.889)	<0.001	1.282 (1.149–1.431)	<0.001	1.229 (1.101–1.373)	<0.001
T3	2.606 (2.355–2.884)	<0.001	1.654 (1.486–1.842)	<0.001	1.481 (1.328–1.651)	<0.001
TyG-BRI tertile groups						
T1	1.00 (Ref)	–	1.00 (Ref)	–	1.00 (Ref)	–
T2	1.074 (0.976–1.183)	0.142	0.966 (0.877–1.064)	0.477	0.882 (0.800–0.972)	0.012
T3	1.266 (1.153–1.392)	<0.001	1.055 (0.958–1.161)	0.280	0.898 (0.813–0.992)	0.034
TyG-CI tertile groups						
T1	1.00 (Ref)	–	1.00 (Ref)	–	1.00 (Ref)	–
T2	1.494 (1.347–1.656)	<0.001	1.195 (1.076–1.328)	<0.001	1.128 (1.015–1.255)	0.026
T3	2.120 (1.921–2.339)	<0.001	1.424 (1.285–1.577)	<0.001	1.244 (1.120–1.381)	<0.001
TyG-RFM tertile groups						
T1	1.00 (Ref)	–	1.00 (Ref)	–	1.00 (Ref)	–
T2	1.061 (0.967–1.163)	0.209	1.136 (1.028–1.256)	0.012	1.012 (0.914–1.120)	0.815
T3	0.910 (0.826–1.002)	0.054	1.184 (1.026–1.366)	0.021	0.963 (0.832–1.114)	0.611
Normal						
TyG index (per SD increase)	1.019 (0.942–1.103)	0.638	0.983 (0.903–1.071)	0.697	0.959 (0.880–1.046)	0.345
TyG-BMI (per SD increase)	0.772 (0.708–0.842)	<0.001	0.794 (0.724–0.872)	<0.001	0.744 (0.676–0.819)	<0.001
TyG-WC (per SD increase)	0.995 (0.919–1.076)	0.892	0.905 (0.831–0.986)	0.023	0.850 (0.778–0.928)	<0.001
TyG-WHtR (per SD increase)	1.031 (0.953–1.116)	0.446	0.943 (0.865–1.027)	0.176	0.884 (0.809–0.966)	0.006
TyG-WWI (per SD increase)	1.375 (1.272–1.487)	<0.001	1.157 (1.060–1.263)	0.001	1.103 (1.009–1.206)	0.031
TyG-ABSI (per SD increase)	1.508 (1.395–1.630)	<0.001	1.230 (1.128–1.340)	<0.001	1.190 (1.091–1.299)	<0.001
TyG-BRI (per SD increase)	1.027 (0.950–1.110)	0.509	0.944 (0.867–1.027)	0.183	0.887 (0.812–0.970)	0.008
TyG-CI (per SD increase)	1.339 (1.239–1.449)	<0.001	1.111 (1.019–1.210)	0.016	1.061 (0.972–1.158)	0.186
TyG-RFM (per SD increase)	0.895 (0.826–0.970)	0.007	0.911 (0.803–1.034)	0.150	0.822 (0.722–0.936)	0.003
TyG index tertile groups						
T1	1.00 (Ref)	–	1.00 (Ref)	–	1.00 (Ref)	–
T2	1.124 (0.923–1.368)	0.245	0.914 (0.748–1.115)	0.375	0.906 (0.741–1.107)	0.334
T3	1.017 (0.835–1.238)	0.870	0.909 (0.742–1.113)	0.356	0.858 (0.700–1.053)	0.143
TyG-BMI tertile groups						
T1	1.00 (Ref)	–	1.00 (Ref)	–	1.00 (Ref)	–
T2	0.758 (0.629–0.912)	0.003	0.723 (0.599–0.873)	<0.001	0.691 (0.572–0.835)	<0.001
T3	0.584 (0.480–0.711)	<0.001	0.621 (0.509–0.758)	<0.001	0.539 (0.440–0.661)	<0.001
TyG-WC tertile groups						
T1	1.00 (Ref)	–	1.00 (Ref)	–	1.00 (Ref)	–
T2	1.011 (0.834–1.227)	0.908	0.862 (0.708–1.048)	0.137	0.792 (0.650–0.966)	0.021
T3	0.966 (0.795–1.174)	0.727	0.806 (0.660–0.984)	0.034	0.694 (0.566–0.850)	<0.001
TyG-WHtR tertile groups						
T1	1.00 (Ref)	–	1.00 (Ref)	–	1.00 (Ref)	–
T2	1.016 (0.835–1.236)	0.876	0.822 (0.674–1.002)	0.053	0.752 (0.615–0.918)	0.005
T3	1.066 (0.879–1.294)	0.516	0.876 (0.718–1.070)	0.195	0.745 (0.607–0.913)	0.005
TyG-WWI tertile groups						
T1	1.00 (Ref)	–	1.00 (Ref)	–	1.00 (Ref)	–
T2	1.223 (0.992–1.508)	0.059	0.867 (0.699–1.075)	0.194	0.811 (0.653–1.007)	0.058
T3	1.769 (1.451–2.155)	<0.001	1.128 (0.912–1.394)	0.266	1.017 (0.821–1.260)	0.875
TyG-ABSI tertile groups						
T1	1.00 (Ref)	–	1.00 (Ref)	–	1.00 (Ref)	–
T2	1.552 (1.246–1.935)	<0.001	1.207 (0.962–1.514)	0.104	1.136 (0.905–1.426)	0.270
T3	2.402 (1.956–2.950)	<0.001	1.465 (1.174–1.827)	<0.001	1.341 (1.073–1.675)	0.010
TyG-BRI tertile groups						
T1	1.00 (Ref)	–	1.00 (Ref)	–	1.00 (Ref)	–
T2	0.989 (0.813–1.203)	0.912	0.800 (0.655–0.976)	0.028	0.757 (0.620–0.926)	0.007
T3	1.100 (0.907–1.334)	0.332	0.896 (0.735–1.093)	0.279	0.753 (0.615–0.922)	0.006
TyG-CI tertile groups						
T1	1.00 (Ref)	–	1.00 (Ref)	–	1.00 (Ref)	–
T2	1.261 (1.020–1.559)	0.032	0.960 (0.773–1.194)	0.715	0.909 (0.731–1.131)	0.392
T3	1.885 (1.547–2.297)	<0.001	1.199 (0.973–1.476)	0.088	1.084 (0.879–1.337)	0.451
TyG-RFM tertile groups						
T1	1.00 (Ref)	–	1.00 (Ref)	–	1.00 (Ref)	–
T2	0.895 (0.740–1.083)	0.254	1.046 (0.838–1.307)	0.689	0.959 (0.766–1.200)	0.711
T3	0.829 (0.684–1.006)	0.058	1.023 (0.760–1.378)	0.878	0.859 (0.635–1.162)	0.324
Dysglycemia						
TyG index (per SD increase)	0.993 (0.937–1.051)	0.799	0.950 (0.893–1.011)	0.108	0.934 (0.877–0.994)	0.033
TyG-BMI (per SD increase)	0.821 (0.769–0.876)	<0.001	0.857 (0.802–0.917)	<0.001	0.836 (0.781–0.894)	<0.001
TyG-WC (per SD increase)	1.021 (0.962–1.083)	0.496	0.929 (0.873–0.989)	0.021	0.895 (0.840–0.954)	<0.001
TyG-WHtR (per SD increase)	1.035 (0.975–1.098)	0.257	0.975 (0.915–1.038)	0.425	0.938 (0.879–1.000)	0.049
TyG-WWI (per SD increase)	1.290 (1.218–1.367)	<0.001	1.126 (1.056–1.200)	<0.001	1.079 (1.011–1.152)	0.022
TyG-ABSI (per SD increase)	1.403 (1.326–1.483)	<0.001	1.154 (1.085–1.229)	<0.001	1.113 (1.045–1.186)	<0.001
TyG-BRI (per SD increase)	1.046 (0.986–1.110)	0.136	0.994 (0.933–1.059)	0.850	0.956 (0.896–1.020)	0.175
TyG-CI (per SD increase)	1.284 (1.212–1.360)	<0.001	1.075 (1.010–1.143)	0.023	1.032 (0.969–1.100)	0.328
TyG-RFM (per SD increase)	0.895 (0.843–0.949)	<0.001	0.937 (0.849–1.033)	0.189	0.883 (0.800–0.976)	0.015
TyG index tertile groups						
T1	1.00 (Ref)	–	1.00 (Ref)	–	1.00 (Ref)	–
T2	1.016 (0.879–1.174)	0.829	0.933 (0.806–1.080)	0.353	0.899 (0.777–1.041)	0.156
T3	0.998 (0.865–1.153)	0.981	0.899 (0.775–1.043)	0.161	0.862 (0.742–1.001)	0.051
TyG-BMI tertile groups						
T1	1.00 (Ref)	–	1.00 (Ref)	–	1.00 (Ref)	–
T2	0.868 (0.756–0.996)	0.044	0.892 (0.776–1.025)	0.108	0.841 (0.731–0.968)	0.016
T3	0.678 (0.587–0.784)	<0.001	0.746 (0.644–0.864)	<0.001	0.712 (0.614–0.827)	<0.001
TyG-WC tertile groups						
T1	1.00 (Ref)	–	1.00 (Ref)	–	1.00 (Ref)	–
T2	0.970 (0.839–1.122)	0.685	0.798 (0.687–0.926)	0.003	0.768 (0.662–0.893)	<0.001
T3	1.048 (0.909–1.208)	0.516	0.857 (0.740–0.992)	0.039	0.787 (0.678–0.913)	0.002
TyG-WHtR tertile groups						
T1	1.00 (Ref)	–	1.00 (Ref)	–	1.00 (Ref)	–
T2	1.074 (0.929–1.241)	0.335	0.997 (0.861–1.154)	0.964	0.941 (0.812–1.091)	0.423
T3	1.104 (0.957–1.275)	0.175	0.992 (0.856–1.151)	0.919	0.923 (0.794–1.072)	0.294
TyG-WWI tertile groups						
T1	1.00 (Ref)	–	1.00 (Ref)	–	1.00 (Ref)	–
T2	1.325 (1.136–1.546)	<0.001	1.124 (0.960–1.316)	0.146	1.082 (0.923–1.267)	0.330
T3	1.756 (1.516–2.035)	<0.001	1.262 (1.078–1.476)	0.004	1.167 (0.995–1.367)	0.057
TyG-ABSI tertile groups						
T1	1.00 (Ref)	–	1.00 (Ref)	–	1.00 (Ref)	–
T2	1.734 (1.475–2.038)	<0.001	1.334 (1.128–1.577)	<0.001	1.312 (1.109–1.552)	0.002
T3	2.287 (1.959–2.670)	<0.001	1.430 (1.211–1.689)	<0.001	1.333 (1.127–1.576)	<0.001
TyG-BRI tertile groups						
T1	1.00 (Ref)	–	1.00 (Ref)	–	1.00 (Ref)	–
T2	0.939 (0.811–1.087)	0.401	0.896 (0.772–1.039)	0.145	0.845 (0.728–0.981)	0.027
T3	1.141 (0.991–1.313)	0.066	1.007 (0.871–1.164)	0.927	0.939 (0.810–1.088)	0.403
TyG-CI tertile groups						
T1	1.00 (Ref)	–	1.00 (Ref)	–	1.00 (Ref)	–
T2	1.327 (1.137–1.550)	<0.001	1.071 (0.913–1.255)	0.400	1.044 (0.890–1.226)	0.596
T3	1.734 (1.496–2.009)	<0.001	1.181 (1.010–1.380)	0.037	1.090 (0.931–1.277)	0.286
TyG-RFM tertile groups						
T1	1.00 (Ref)	–	1.00 (Ref)	–	1.00 (Ref)	–
T2	1.001 (0.872–1.148)	0.992	1.034 (0.890–1.202)	0.660	0.985 (0.845–1.147)	0.844
T3	0.778 (0.672–0.901)	<0.001	0.930 (0.739–1.169)	0.532	0.871 (0.691–1.098)	0.242
Diabetes						
TyG index (per SD increase)	1.013 (0.949–1.081)	0.699	1.042 (0.972–1.117)	0.244	1.015 (0.948–1.088)	0.664
TyG-BMI (per SD increase)	0.820 (0.761–0.884)	<0.001	0.846 (0.782–0.915)	<0.001	0.790 (0.729–0.857)	<0.001
TyG-WC (per SD increase)	0.979 (0.913–1.051)	0.564	0.929 (0.862–1.001)	0.054	0.875 (0.811–0.945)	<0.001
TyG-WHtR (per SD increase)	0.958 (0.893–1.027)	0.226	0.950 (0.881–1.023)	0.176	0.893 (0.827–0.964)	0.004
TyG-WWI (per SD increase)	1.136 (1.064–1.212)	<0.001	1.114 (1.038–1.195)	0.003	1.076 (1.002–1.155)	0.044
TyG-ABSI (per SD increase)	1.230 (1.155–1.309)	<0.001	1.163 (1.087–1.244)	<0.001	1.141 (1.067–1.221)	<0.001
TyG-BRI (per SD increase)	0.949 (0.883–1.021)	0.159	0.935 (0.865–1.009)	0.085	0.877 (0.810–0.950)	0.001
TyG-CI (per SD increase)	1.158 (1.085–1.235)	<0.001	1.093 (1.020–1.172)	0.012	1.057 (0.986–1.134)	0.120
TyG-RFM (per SD increase)	0.885 (0.827–0.948)	<0.001	0.915 (0.812–1.030)	0.141	0.832 (0.738–0.939)	0.003
TyG index tertile groups						
T1	1.00 (Ref)	–	1.00 (Ref)	–	1.00 (Ref)	–
T2	0.944 (0.798–1.116)	0.498	0.912 (0.768–1.082)	0.290	0.893 (0.752–1.060)	0.196
T3	0.999 (0.848–1.175)	0.987	1.030 (0.870–1.219)	0.730	0.976 (0.823–1.157)	0.780
TyG-BMI tertile groups						
T1	1.00 (Ref)	–	1.00 (Ref)	–	1.00 (Ref)	–
T2	0.810 (0.691–0.949)	0.009	0.796 (0.678–0.934)	0.005	0.754 (0.641–0.888)	<0.001
T3	0.697 (0.589–0.824)	<0.001	0.731 (0.616–0.867)	<0.001	0.636 (0.534–0.757)	<0.001
TyG-WC tertile groups						
T1	1.00 (Ref)	–	1.00 (Ref)	–	1.00 (Ref)	–
T2	0.961 (0.818–1.130)	0.633	0.862 (0.732–1.016)	0.076	0.823 (0.697–0.970)	0.020
T3	0.916 (0.775–1.082)	0.300	0.817 (0.688–0.970)	0.021	0.709 (0.595–0.846)	<0.001
TyG-WHtR tertile groups						
T1	1.00 (Ref)	–	1.00 (Ref)	–	1.00 (Ref)	–
T2	1.044 (0.889–1.227)	0.600	0.996 (0.846–1.174)	0.965	0.954 (0.809–1.126)	0.580
T3	0.922 (0.779–1.091)	0.342	0.912 (0.766–1.086)	0.300	0.789 (0.659–0.943)	0.009
TyG-WWI tertile groups						
T1	1.00 (Ref)	–	1.00 (Ref)	–	1.00 (Ref)	–
T2	1.084 (0.916–1.284)	0.348	1.028 (0.865–1.221)	0.754	0.973 (0.818–1.157)	0.757
T3	1.282 (1.087–1.512)	0.003	1.234 (1.033–1.474)	0.020	1.152 (0.963–1.378)	0.123
TyG-ABSI tertile groups						
T1	1.00 (Ref)	–	1.00 (Ref)	–	1.00 (Ref)	–
T2	1.283 (1.078–1.528)	0.005	1.136 (0.949–1.359)	0.164	1.136 (0.949–1.360)	0.166
T3	1.598 (1.351–1.891)	<0.001	1.428 (1.196–1.703)	<0.001	1.387 (1.161–1.657)	<0.001
TyG-BRI tertile groups						
T1	1.00 (Ref)	–	1.00 (Ref)	–	1.00 (Ref)	–
T2	0.992 (0.844–1.165)	0.919	0.887 (0.752–1.047)	0.156	0.848 (0.718–1.002)	0.053
T3	0.926 (0.783–1.095)	0.370	0.857 (0.720–1.020)	0.082	0.747 (0.624–0.893)	0.001
TyG-CI tertile groups						
T1	1.00 (Ref)	–	1.00 (Ref)	–	1.00 (Ref)	–
T2	1.094 (0.922–1.297)	0.303	0.985 (0.828–1.171)	0.861	0.942 (0.791–1.121)	0.502
T3	1.352 (1.146–1.595)	<0.001	1.204 (1.013–1.431)	0.036	1.130 (0.949–1.346)	0.170
TyG-RFM tertile groups						
T1	1.00 (Ref)	–	1.00 (Ref)	–	1.00 (Ref)	–
T2	0.930 (0.793–1.090)	0.369	0.982 (0.822–1.174)	0.844	0.906 (0.758–1.085)	0.283
T3	0.744 (0.629–0.880)	<0.001	0.829 (0.632–1.087)	0.175	0.723 (0.549–0.952)	0.021

**CVD mortality**						

Total						
TyG index (per SD increase)	1.174 (1.101–1.252)	<0.001	1.152 (1.076–1.233)	<0.001	1.078 (1.005–1.156)	0.036
TyG-BMI (per SD increase)	1.014 (0.945–1.088)	0.696	1.042 (0.968–1.121)	0.279	0.934 (0.866–1.009)	0.082
TyG-WC (per SD increase)	1.219 (1.140–1.304)	<0.001	1.123 (1.045–1.206)	0.002	1.006 (0.933–1.084)	0.879
TyG-WHtR (per SD increase)	1.242 (1.161–1.328)	<0.001	1.182 (1.100–1.270)	<0.001	1.057 (0.980–1.139)	0.152
TyG-WWI (per SD increase)	1.449 (1.360–1.544)	<0.001	1.329 (1.238–1.427)	<0.001	1.216 (1.129–1.309)	<0.001
TyG-ABSI (per SD increase)	1.495 (1.405–1.591)	<0.001	1.310 (1.222–1.403)	<0.001	1.226 (1.142–1.317)	<0.001
TyG-BRI (per SD increase)	1.215 (1.138–1.296)	<0.001	1.163 (1.083–1.248)	<0.001	1.045 (0.970–1.125)	0.248
TyG-CI (per SD increase)	1.444 (1.354–1.539)	<0.001	1.274 (1.187–1.366)	<0.001	1.167 (1.085–1.256)	<0.001
TyG-RFM (per SD increase)	1.013 (0.946–1.086)	0.708	1.267 (1.138–1.412)	<0.001	1.073 (0.960–1.200)	0.215
TyG index tertile groups						
T1	1.00 (Ref)	–	1.00 (Ref)	–	1.00 (Ref)	–
T2	1.225 (1.030–1.458)	0.022	1.147 (0.962–1.368)	0.126	1.068 (0.895–1.274)	0.464
T3	1.403 (1.183–1.663)	<0.001	1.280 (1.076–1.523)	0.005	1.098 (0.920–1.310)	0.300
TyG-BMI tertile groups						
T1	1.00 (Ref)	–	1.00 (Ref)	–	1.00 (Ref)	–
T2	1.000 (0.845–1.183)	1.000	0.978 (0.826–1.158)	0.796	0.850 (0.717–1.008)	0.061
T3	1.046 (0.886–1.236)	0.592	1.090 (0.922–1.289)	0.314	0.860 (0.724–1.022)	0.087
TyG-WC tertile groups						
T1	1.00 (Ref)	–	1.00 (Ref)	–	1.00 (Ref)	–
T2	1.213 (1.018–1.446)	0.031	1.008 (0.844–1.204)	0.931	0.898 (0.751–1.074)	0.239
T3	1.537 (1.298–1.820)	<0.001	1.245 (1.048–1.480)	0.013	0.986 (0.826–1.177)	0.877
TyG-WHtR tertile groups						
T1	1.00 (Ref)	–	1.00 (Ref)	–	1.00 (Ref)	–
T2	1.226 (1.030–1.461)	0.022	1.100 (0.922–1.311)	0.291	0.953 (0.798–1.138)	0.595
T3	1.552 (1.310–1.838)	<0.001	1.348 (1.133–1.602)	<0.001	1.062 (0.889–1.269)	0.505
TyG-WWI tertile groups						
T1	1.00 (Ref)	–	1.00 (Ref)	–	1.00 (Ref)	–
T2	1.807 (1.497–2.181)	<0.001	1.506 (1.244–1.824)	<0.001	1.371 (1.131–1.662)	0.001
T3	2.544 (2.122–3.049)	<0.001	1.925 (1.591–2.329)	<0.001	1.596 (1.315–1.936)	<0.001
TyG-ABSI tertile groups						
T1	1.00 (Ref)	–	1.00 (Ref)	–	1.00 (Ref)	–
T2	1.963 (1.620–2.378)	<0.001	1.540 (1.264–1.877)	<0.001	1.442 (1.182–1.760)	<0.001
T3	2.818 (2.342–3.390)	<0.001	1.880 (1.546–2.287)	<0.001	1.596 (1.308–1.946)	<0.001
TyG-BRI tertile groups						
T1	1.00 (Ref)	–	1.00 (Ref)	–	1.00 (Ref)	–
T2	1.145 (0.961–1.364)	0.129	1.037 (0.869–1.237)	0.688	0.902 (0.755–1.078)	0.257
T3	1.530 (1.294–1.809)	<0.001	1.309 (1.103–1.554)	0.002	1.029 (0.864–1.227)	0.746
TyG-CI tertile groups						
T1	1.00 (Ref)	–	1.00 (Ref)	–	1.00 (Ref)	–
T2	1.651 (1.370–1.990)	<0.001	1.356 (1.121–1.641)	0.002	1.235 (1.019–1.496)	0.031
T3	2.377 (1.989–2.840)	<0.001	1.676 (1.392–2.018)	<0.001	1.378 (1.140–1.666)	<0.001
TyG-RFM tertile groups						
T1	1.00 (Ref)	–	1.00 (Ref)	–	1.00 (Ref)	–
T2	1.138 (0.964–1.343)	0.126	1.351 (1.132–1.612)	<0.001	1.116 (0.932–1.336)	0.233
T3	1.025 (0.864–1.216)	0.778	1.711 (1.323–2.213)	<0.001	1.242 (0.956–1.613)	0.104
Normal						
TyG index (per SD increase)	0.940 (0.805–1.098)	0.435	0.898 (0.759–1.063)	0.210	0.825 (0.695–0.980)	0.029
TyG-BMI (per SD increase)	0.798 (0.676–0.943)	0.008	0.816 (0.680–0.978)	0.028	0.724 (0.598–0.878)	<0.001
TyG-WC (per SD increase)	1.008 (0.865–1.175)	0.915	0.910 (0.768–1.077)	0.271	0.814 (0.682–0.973)	0.023
TyG-WHtR (per SD increase)	1.071 (0.921–1.246)	0.374	0.975 (0.824–1.153)	0.766	0.868 (0.727–1.036)	0.117
TyG-WWI (per SD increase)	1.390 (1.194–1.618)	<0.001	1.163 (0.979–1.381)	0.086	1.047 (0.877–1.250)	0.610
TyG-ABSI (per SD increase)	1.464 (1.259–1.704)	<0.001	1.184 (0.998–1.404)	0.053	1.097 (0.922–1.306)	0.296
TyG-BRI (per SD increase)	1.091 (0.942–1.263)	0.246	1.004 (0.853–1.181)	0.962	0.912 (0.766–1.085)	0.297
TyG-CI (per SD increase)	1.326 (1.139–1.544)	<0.001	1.090 (0.920–1.290)	0.319	0.989 (0.830–1.178)	0.899
TyG-RFM (per SD increase)	0.901 (0.771–1.054)	0.193	0.951 (0.740–1.222)	0.694	0.788 (0.607–1.023)	0.074
TyG index tertile groups						
T1	1.00 (Ref)	–	1.00 (Ref)	–	1.00 (Ref)	–
T2	1.110 (0.764–1.613)	0.584	0.907 (0.621–1.326)	0.615	0.913 (0.621–1.342)	0.644
T3	0.896 (0.609–1.317)	0.576	0.803 (0.540–1.194)	0.278	0.683 (0.453–1.029)	0.069
TyG-BMI tertile groups						
T1	1.00 (Ref)	–	1.00 (Ref)	–	1.00 (Ref)	–
T2	0.749 (0.521–1.076)	0.118	0.685 (0.474–0.991)	0.045	0.611 (0.420–0.890)	0.010
T3	0.609 (0.417–0.889)	0.010	0.630 (0.429–0.925)	0.018	0.482 (0.324–0.717)	<0.001
TyG-WC tertile groups						
T1	1.00 (Ref)	–	1.00 (Ref)	–	1.00 (Ref)	–
T2	1.061 (0.729–1.542)	0.758	0.866 (0.592–1.268)	0.460	0.774 (0.525–1.141)	0.195
T3	0.981 (0.670–1.436)	0.921	0.796 (0.537–1.178)	0.254	0.624 (0.416–0.935)	0.022
TyG-WHtR tertile groups						
T1	1.00 (Ref)	–	1.00 (Ref)	–	1.00 (Ref)	–
T2	0.882 (0.598–1.300)	0.526	0.691 (0.466–1.024)	0.066	0.652 (0.437–0.973)	0.036
T3	1.118 (0.775–1.613)	0.552	0.882 (0.604–1.289)	0.516	0.683 (0.461–1.011)	0.057
TyG-WWI tertile groups						
T1	1.00 (Ref)	–	1.00 (Ref)	–	1.00 (Ref)	–
T2	1.052 (0.702–1.577)	0.805	0.754 (0.497–1.143)	0.183	0.689 (0.453–1.049)	0.083
T3	1.644 (1.131–2.391)	0.009	1.018 (0.680–1.525)	0.930	0.840 (0.556–1.270)	0.409
TyG-ABSI tertile groups						
T1	1.00 (Ref)	–	1.00 (Ref)	–	1.00 (Ref)	–
T2	1.150 (0.760–1.738)	0.509	0.869 (0.568–1.331)	0.519	0.789 (0.513–1.213)	0.280
T3	1.895 (1.301–2.760)	<0.001	1.100 (0.731–1.655)	0.648	0.938 (0.620–1.420)	0.763
TyG-BRI tertile groups						
T1	1.00 (Ref)	–	1.00 (Ref)	–	1.00 (Ref)	–
T2	1.043 (0.702–1.550)	0.836	0.803 (0.536–1.201)	0.285	0.775 (0.515–1.167)	0.222
T3	1.397 (0.961–2.030)	0.080	1.081 (0.735–1.591)	0.691	0.839 (0.563–1.252)	0.391
TyG-CI tertile groups						
T1	1.00 (Ref)	–	1.00 (Ref)	–	1.00 (Ref)	–
T2	0.953 (0.637–1.425)	0.815	0.703 (0.465–1.061)	0.093	0.658 (0.433–0.998)	0.049
T3	1.493 (1.035–2.153)	0.032	0.915 (0.620–1.350)	0.653	0.750 (0.504–1.115)	0.155
TyG-RFM tertile groups						
T1	1.00 (Ref)	–	1.00 (Ref)	–	1.00 (Ref)	–
T2	1.001 (0.689–1.455)	0.994	1.288 (0.842–1.970)	0.243	1.040 (0.673–1.607)	0.859
T3	0.957 (0.656–1.396)	0.819	1.544 (0.857–2.780)	0.148	1.099 (0.599–2.015)	0.761
Dysglycemia						
TyG index (per SD increase)	0.993 (0.897–1.098)	0.886	0.980 (0.879–1.092)	0.717	0.936 (0.838–1.045)	0.240
TyG-BMI (per SD increase)	0.883 (0.790–0.986)	0.027	0.949 (0.846–1.064)	0.366	0.893 (0.795–1.004)	0.059
TyG-WC (per SD increase)	1.078 (0.973–1.195)	0.149	1.010 (0.906–1.125)	0.860	0.936 (0.837–1.047)	0.247
TyG-WHtR (per SD increase)	1.113 (1.005–1.233)	0.040	1.088 (0.975–1.213)	0.133	1.003 (0.896–1.122)	0.960
TyG-WWI (per SD increase)	1.349 (1.220–1.492)	<0.001	1.224 (1.095–1.367)	<0.001	1.120 (1.000–1.255)	0.050
TyG-ABSI (per SD increase)	1.408 (1.277–1.553)	<0.001	1.185 (1.063–1.321)	0.002	1.102 (0.985–1.232)	0.089
TyG-BRI (per SD increase)	1.127 (1.020–1.246)	0.019	1.108 (0.995–1.234)	0.063	1.026 (0.918–1.147)	0.647
TyG-CI (per SD increase)	1.324 (1.198–1.464)	<0.001	1.143 (1.026–1.274)	0.016	1.052 (0.941–1.176)	0.372
TyG-RFM (per SD increase)	0.923 (0.832–1.023)	0.127	1.119 (0.940–1.333)	0.206	0.986 (0.825–1.178)	0.877
TyG index tertile groups						
T1	1.00 (Ref)	–	1.00 (Ref)	–	1.00 (Ref)	–
T2	1.137 (0.883–1.465)	0.319	1.078 (0.835–1.393)	0.564	1.006 (0.778–1.301)	0.961
T3	1.030 (0.797–1.332)	0.821	0.998 (0.765–1.301)	0.987	0.895 (0.685–1.169)	0.415
TyG-BMI tertile groups						
T1	1.00 (Ref)	–	1.00 (Ref)	–	1.00 (Ref)	–
T2	0.919 (0.718–1.175)	0.500	0.962 (0.751–1.233)	0.761	0.852 (0.663–1.095)	0.212
T3	0.789 (0.614–1.014)	0.064	0.909 (0.705–1.174)	0.465	0.799 (0.617–1.034)	0.088
TyG-WC tertile groups						
T1	1.00 (Ref)	–	1.00 (Ref)	–	1.00 (Ref)	–
T2	1.160 (0.897–1.499)	0.257	0.979 (0.753–1.273)	0.875	0.904 (0.695–1.178)	0.456
T3	1.163 (0.902–1.500)	0.245	1.007 (0.775–1.310)	0.957	0.845 (0.647–1.104)	0.218
TyG-WHtR tertile groups						
T1	1.00 (Ref)	–	1.00 (Ref)	–	1.00 (Ref)	–
T2	1.185 (0.916–1.533)	0.196	1.133 (0.873–1.470)	0.349	1.014 (0.780–1.318)	0.916
T3	1.227 (0.951–1.582)	0.116	1.169 (0.899–1.520)	0.244	0.976 (0.748–1.273)	0.857
TyG-WWI tertile groups						
T1	1.00 (Ref)	–	1.00 (Ref)	–	1.00 (Ref)	–
T2	1.778 (1.343–2.355)	<0.001	1.581 (1.186–2.106)	0.002	1.419 (1.063–1.894)	0.017
T3	2.181 (1.658–2.868)	<0.001	1.707 (1.275–2.284)	<0.001	1.432 (1.068–1.920)	0.016
TyG-ABSI tertile groups						
T1	1.00 (Ref)	–	1.00 (Ref)	–	1.00 (Ref)	–
T2	2.328 (1.734–3.125)	<0.001	1.896 (1.397–2.574)	<0.001	1.765 (1.298–2.399)	<0.001
T3	2.670 (1.999–3.567)	<0.001	1.807 (1.324–2.465)	<0.001	1.541 (1.126–2.109)	0.007
TyG-BRI tertile groups						
T1	1.00 (Ref)	–	1.00 (Ref)	–	1.00 (Ref)	–
T2	1.008 (0.775–1.311)	0.951	0.985 (0.755–1.286)	0.914	0.878 (0.672–1.149)	0.343
T3	1.331 (1.038–1.705)	0.024	1.231 (0.953–1.591)	0.112	1.046 (0.807–1.355)	0.736
TyG-CI tertile groups						
T1	1.00 (Ref)	–	1.00 (Ref)	–	1.00 (Ref)	–
T2	1.612 (1.222–2.126)	<0.001	1.356 (1.022–1.801)	0.035	1.236 (0.928–1.646)	0.147
T3	1.935 (1.480–2.532)	<0.001	1.420 (1.069–1.886)	0.015	1.201 (0.901–1.601)	0.212
TyG-RFM tertile groups						
T1	1.00 (Ref)	–	1.00 (Ref)	–	1.00 (Ref)	–
T2	1.029 (0.807–1.311)	0.818	1.158 (0.891–1.504)	0.272	0.996 (0.764–1.300)	0.978
T3	0.811 (0.628–1.049)	0.111	1.203 (0.801–1.805)	0.373	0.987 (0.656–1.485)	0.950
Diabetes						
TyG index (per SD increase)	1.038 (0.931–1.158)	0.497	1.104 (0.983–1.239)	0.094	1.081 (0.963–1.213)	0.188
TyG-BMI (per SD increase)	0.884 (0.781–1.000)	0.051	0.925 (0.812–1.054)	0.242	0.864 (0.755–0.987)	0.032
TyG-WC (per SD increase)	1.021 (0.907–1.149)	0.735	0.989 (0.872–1.121)	0.861	0.934 (0.820–1.063)	0.298
TyG-WHtR (per SD increase)	1.003 (0.891–1.129)	0.961	1.019 (0.898–1.156)	0.772	0.958 (0.841–1.090)	0.513
TyG-WWI (per SD increase)	1.144 (1.026–1.277)	0.016	1.158 (1.028–1.303)	0.015	1.121 (0.995–1.263)	0.061
TyG-ABSI (per SD increase)	1.215 (1.092–1.351)	<0.001	1.180 (1.053–1.323)	0.004	1.166 (1.041–1.307)	0.008
TyG-BRI (per SD increase)	0.995 (0.882–1.122)	0.933	0.995 (0.875–1.131)	0.938	0.933 (0.816–1.066)	0.306
TyG-CI (per SD increase)	1.164 (1.044–1.299)	0.006	1.132 (1.007–1.273)	0.037	1.101 (0.978–1.239)	0.112
TyG-RFM (per SD increase)	0.913 (0.813–1.025)	0.122	1.015 (0.829–1.242)	0.888	0.920 (0.749–1.131)	0.428
TyG index tertile groups						
T1	1.00 (Ref)	–	1.00 (Ref)	–	1.00 (Ref)	–
T2	0.804 (0.600–1.076)	0.142	0.819 (0.608–1.104)	0.190	0.815 (0.605–1.099)	0.181
T3	1.036 (0.792–1.357)	0.794	1.143 (0.865–1.511)	0.348	1.095 (0.825–1.452)	0.531
TyG-BMI tertile groups						
T1	1.00 (Ref)	–	1.00 (Ref)	–	1.00 (Ref)	–
T2	0.847 (0.646–1.111)	0.231	0.847 (0.643–1.116)	0.237	0.793 (0.600–1.049)	0.104
T3	0.771 (0.581–1.024)	0.072	0.820 (0.614–1.094)	0.177	0.707 (0.526–0.949)	0.021
TyG-WC tertile groups						
T1	1.00 (Ref)	–	1.00 (Ref)	–	1.00 (Ref)	–
T2	0.957 (0.725–1.264)	0.758	0.872 (0.658–1.156)	0.341	0.838 (0.631–1.114)	0.225
T3	1.014 (0.767–1.341)	0.922	0.945 (0.708–1.260)	0.699	0.830 (0.617–1.117)	0.219
TyG-WHtR tertile groups						
T1	1.00 (Ref)	–	1.00 (Ref)	–	1.00 (Ref)	–
T2	1.184 (0.898–1.562)	0.232	1.157 (0.874–1.533)	0.308	1.104 (0.832–1.466)	0.493
T3	1.044 (0.782–1.394)	0.768	1.081 (0.801–1.458)	0.611	0.932 (0.685–1.266)	0.651
TyG-WWI tertile groups						
T1	1.00 (Ref)	–	1.00 (Ref)	–	1.00 (Ref)	–
T2	0.989 (0.743–1.315)	0.937	0.968 (0.725–1.294)	0.828	0.925 (0.691–1.239)	0.603
T3	1.194 (0.905–1.574)	0.209	1.235 (0.916–1.665)	0.165	1.155 (0.855–1.561)	0.349
TyG-ABSI tertile groups						
T1	1.00 (Ref)	–	1.00 (Ref)	–	1.00 (Ref)	–
T2	1.163 (0.865–1.564)	0.317	1.057 (0.779–1.435)	0.721	1.069 (0.787–1.453)	0.668
T3	1.581 (1.195–2.093)	0.001	1.506 (1.120–2.024)	0.007	1.483 (1.102–1.997)	0.009
TyG-BRI tertile groups						
T1	1.00 (Ref)	–	1.00 (Ref)	–	1.00 (Ref)	–
T2	0.983 (0.745–1.299)	0.906	0.896 (0.674–1.192)	0.452	0.846 (0.635–1.127)	0.253
T3	1.036 (0.782–1.371)	0.808	0.989 (0.739–1.324)	0.941	0.858 (0.636–1.157)	0.315
TyG-CI tertile groups						
T1	1.00 (Ref)	–	1.00 (Ref)	–	1.00 (Ref)	–
T2	0.987 (0.736–1.323)	0.930	0.906 (0.672–1.220)	0.515	0.869 (0.644–1.172)	0.358
T3	1.389 (1.055–1.829)	0.019	1.325 (0.992–1.769)	0.057	1.256 (0.937–1.683)	0.127
TyG-RFM tertile groups						
T1	1.00 (Ref)	–	1.00 (Ref)	–	1.00 (Ref)	–
T2	1.052 (0.801–1.383)	0.714	1.192 (0.881–1.612)	0.254	1.098 (0.811–1.486)	0.546
T3	1.144 (0.845–1.550)	0.381	1.480 (1.075–2.048)	0.017	1.484 (1.103–1.998)	0.009

Crude model: Unadjusted.

Model 1: age, gender, race, education, PIR, marital status, smoking, and alcohol use.

Model 2: age, gender, race, education, PIR, marital status, smoking, alcohol use, antihypertensive drug, hypertension, heart failure, heart disease, angina, heart attack, stroke, failing kidney, and cancer.

Continuous HRs are expressed per 1-SD increase. For total-population analyses, SDs were calculated from the overall analytic sample; for glycaemic-status-stratified analyses, SDs were calculated separately within each corresponding glycemic stratum. Therefore, per-SD HRs in the total population and in glycemic subgroups should be interpreted as population-specific standardized associations.

TyG index, triglyceride-glucose index; TyG-BMI, triglyceride-glucose index combined with body mass index; TyG-WC, triglyceride-glucose index combined with waist circumference; TyG-WHtR, triglyceride-glucose index combined with waist-to-height ratio; TyG-WWI, triglyceride-glucose index combined with weight-adjusted waist index; TyG-ABSI, triglyceride-glucose index combined with a body shape index; TyG-BRI, triglyceride-glucose index combined with body roundness index; TyG-CI, triglyceride-glucose index combined with conicity index; TyG-RFM, triglyceride-glucose index combined with relative fat mass; HR, hazard ratio; CI, confidence interval.

The association between TyG-ABSI and mortality varied by glucose metabolism status. For all-cause mortality, TyG-ABSI showed consistent positive associations across all glycemic strata in both continuous analyses (normal: HR = 1.190, *p* < 0.001; dysglycemia: HR = 1.113, *p* < 0.001; diabetes: HR = 1.141, *p* < 0.001) and categorical comparisons (T3 vs. T1: normal: HR = 1.341, *p* = 0.010; dysglycemia: HR = 1.333, *p* < 0.001; diabetes: HR = 1.387, *p* < 0.001). For cardiovascular mortality, however, the association demonstrated heterogeneity by glycemic status: significant associations were observed only in participants with diabetes (HR = 1.166 per SD, *p* = 0.008; T3 vs. T1: HR = 1.483, *p* = 0.009), whereas associations were attenuated in dysglycemia (HR = 1.102 per SD, *p* = 0.089; T3 vs. T1: HR = 1.541, *p* = 0.007) and absent in normal glucose metabolism (HR = 1.097 per SD, *p* = 0.296; T3 vs. T1: HR = 0.938, *p* = 0.763). Notably, indices reflecting overall adiposity showed inverse associations with all-cause mortality; TyG-BMI was inversely associated across all strata in both continuous and categorical analyses (total population: HR = 0.855 per SD, *p* < 0.001; T3 vs. T1: HR = 0.728, *p* < 0.001). Detailed findings for other TyG-derived indices are provided in the supplemental information.

Multicollinearity diagnostics indicated no evidence of severe collinearity among covariates, with variance inflation factor (VIF) values ranging from 1.04 to 1.72 across all populations, well below the conventional threshold of 10 (Figure S1).

### Linear relationships

**Figure 2:**
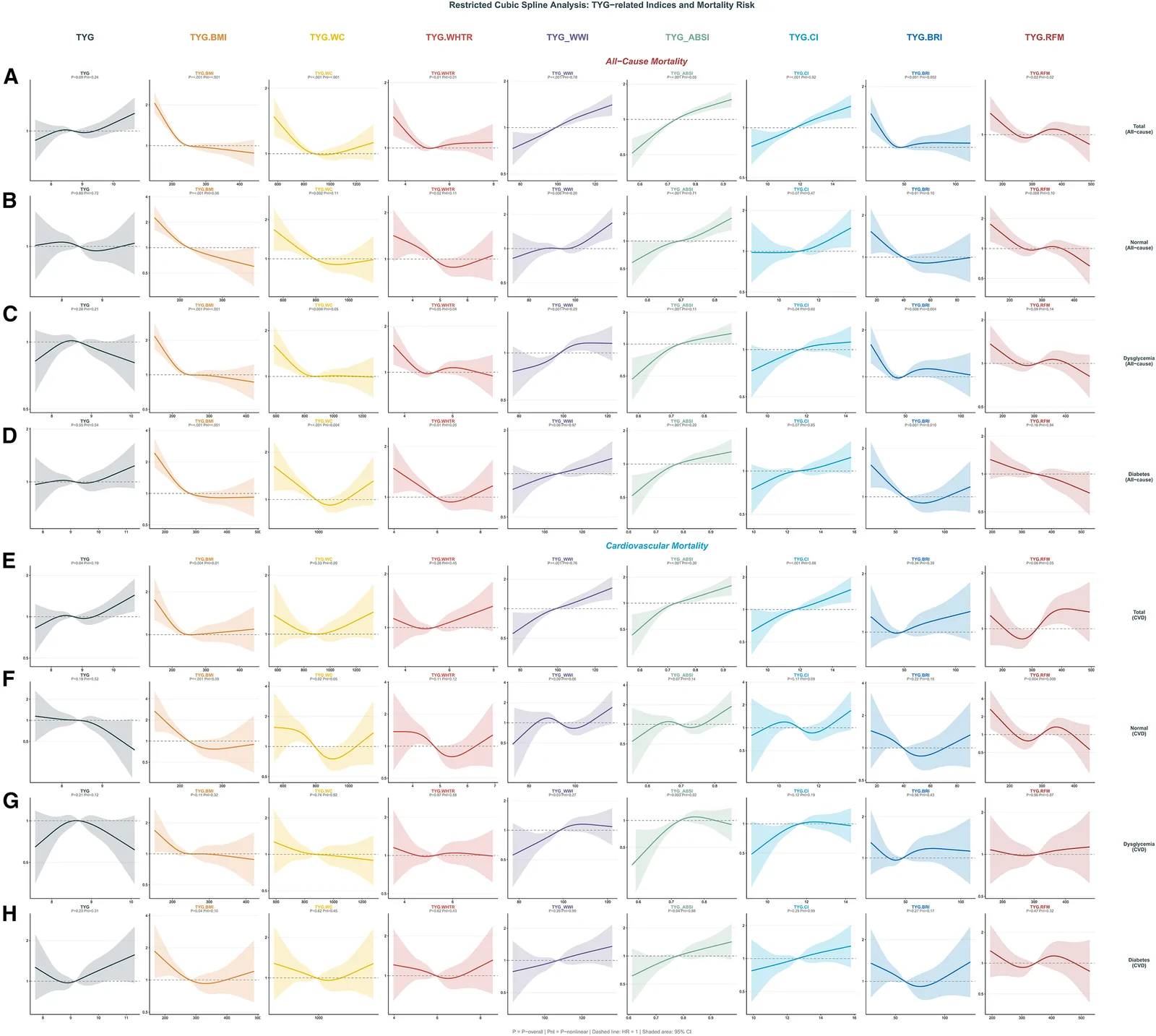
Dose-response associations of the TyG index and TyG-related parameters with the risks of all-cause and cardiovascular mortality using restricted cubic spline analyses Restricted cubic spline analyses of the association between the TyG index and TyG-related parameters (TyG-BMI, TyG-WC, TyG-WHtR, TyG-WWI, TyG-ABSI, TyG-CI, TyG-BRI, and TyG-RFM) and the risk of all-cause mortality (A–D) and cardiovascular mortality (E–H). A and E show the total population, B and F show participants with normal glucose tolerance, C and G show those with dysglycemia, and D and H show those with diabetes. Solid lines represent multivariable-adjusted hazard ratios, and shaded areas indicate 95% confidence intervals. The vertical dashed line denotes the reference value (set at the median level), and the horizontal dashed line indicates a hazard ratio of 1.0. P values shown in each panel correspond to tests for the overall association and for nonlinearity. All models were survey-weighted Cox proportional hazards models adjusted according to the fully adjusted model. Abbreviations. TyG, triglyceride-glucose index; BMI, body mass index; WC, waist circumference; WHtR, waist-to-height ratio; WWI, weight-adjusted waist index; ABSI, a body shape index; CI, conicity index; BRI, body roundness index; RFM, relative fat mass.

Restricted cubic spline analyses revealed dose-response associations between TyG-related indices and mortality (Figure 2). In the total population, TyG-ABSI demonstrated the steepest and most consistent risk gradient for both all-cause mortality (*P* for overall <0.001; *P* for nonlinearity = 0.05) and cardiovascular mortality (*P* for overall <0.001; P for nonlinearity = 0.30), with mortality risk increasing steadily across the exposure range in an approximately linear fashion. In contrast, the original TyG index showed no significant association with all-cause mortality (*p* = 0.09) and only a modest association with cardiovascular mortality (*p* = 0.04).

Across glucose metabolism strata, TyG-ABSI exhibited the most robust dose-response relationship with mortality. For all-cause mortality, significant positive associations were observed consistently across normal glucose metabolism (*p* < 0.001), dysglycemia (*p* < 0.001), and diabetes (*p* < 0.001). For cardiovascular mortality, however, the association varied by glycemic status: significant in dysglycemia (*p* = 0.003) and diabetes (*p* = 0.04), but attenuated in normal glucose metabolism (*p* = 0.07). Other TyG-derived indices showed more heterogeneous patterns, with indices reflecting overall adiposity (TyG-BMI, TyG-WC) demonstrating significant but often nonlinear associations with all-cause mortality, whereas their associations with cardiovascular mortality were weaker and less consistent across glycemic strata. Detailed dose-response findings for all TyG-derived indices are provided in the supplemental information.

### The combined effect of glucose metabolism status and TyG-ABSI on mortality

**Figure 3:**
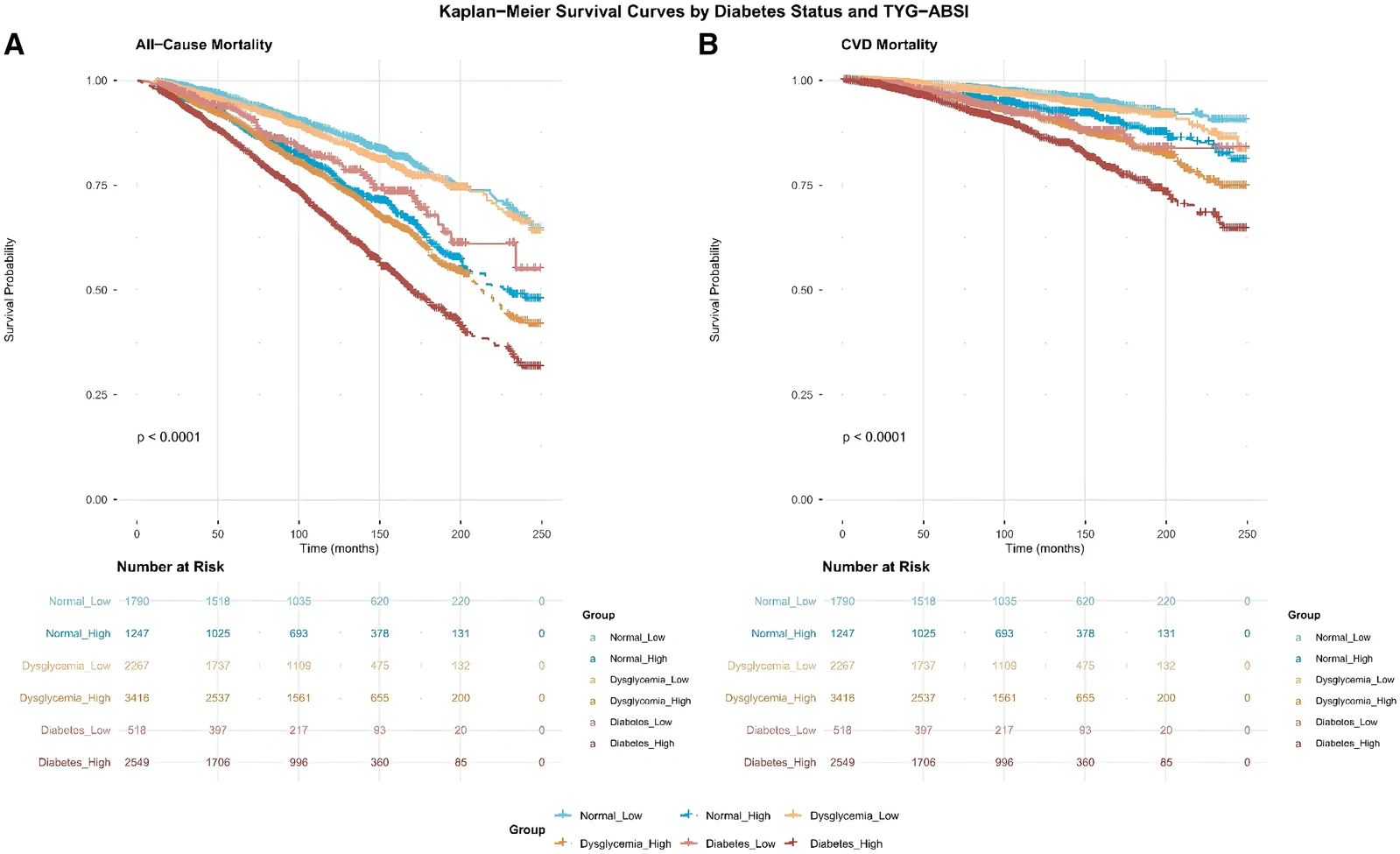
Kaplan-Meier survival estimates, stratified by combined glucose metabolism status and TyG-ABSI levels, assessed all-cause mortality and cardiovascular mortality (A) Kaplan-Meier curves for all-cause mortality. (B) Kaplan-Meier curves for cardiovascular disease (CVD) mortality. The study population was divided into six subgroups based on glycemic status (normal, impaired glucose tolerance, diabetes) and TyG-ABSI category (low, high). This *p* value indicates significant differences between groups, determined by the log rank test. Abbreviations: cardiovascular disease (CVD); TyG-ABSI, triglyceride-glucose index combined with a body shape index.

To assess the combined exposure effects of TyG-ABSI and glucose metabolism status, we divided the population into low- and high-risk groups using the optimal cutoff point for TyG-ABSI (0.71). These groups were then crossed with three glucose metabolism statuses (normal glucose metabolism, impaired glucose tolerance/impaired glucose metabolism, diabetes) to form six subgroups, with “normal glucose metabolism and low TyG-ABSI” serving as the reference group. Kaplan-Meier curves (Figure 3) revealed significant differences in survival probabilities for all-cause mortality and cardiovascular mortality across these six groups (log rank test, *p* < 0.0001 for both endpoints). Overall, survival curves exhibited clear gradient separation: within the same glucose metabolism stratum, survival rates in the high TyG-ABSI group consistently lagged behind those in the low TyG-ABSI group. Within the same TyG-ABSI level, survival rates progressively declined, and risks increased sequentially as glucose metabolism status progressed from normal to impaired glucose tolerance to diabetes. Among these, the curve for the diabetes with high TyG-ABSI group was the lowest and declined most steeply, indicating the strongest mortality risk under combined exposure. This adverse effect progressively accumulated and amplified throughout the follow-up period.

**Figure 4:**
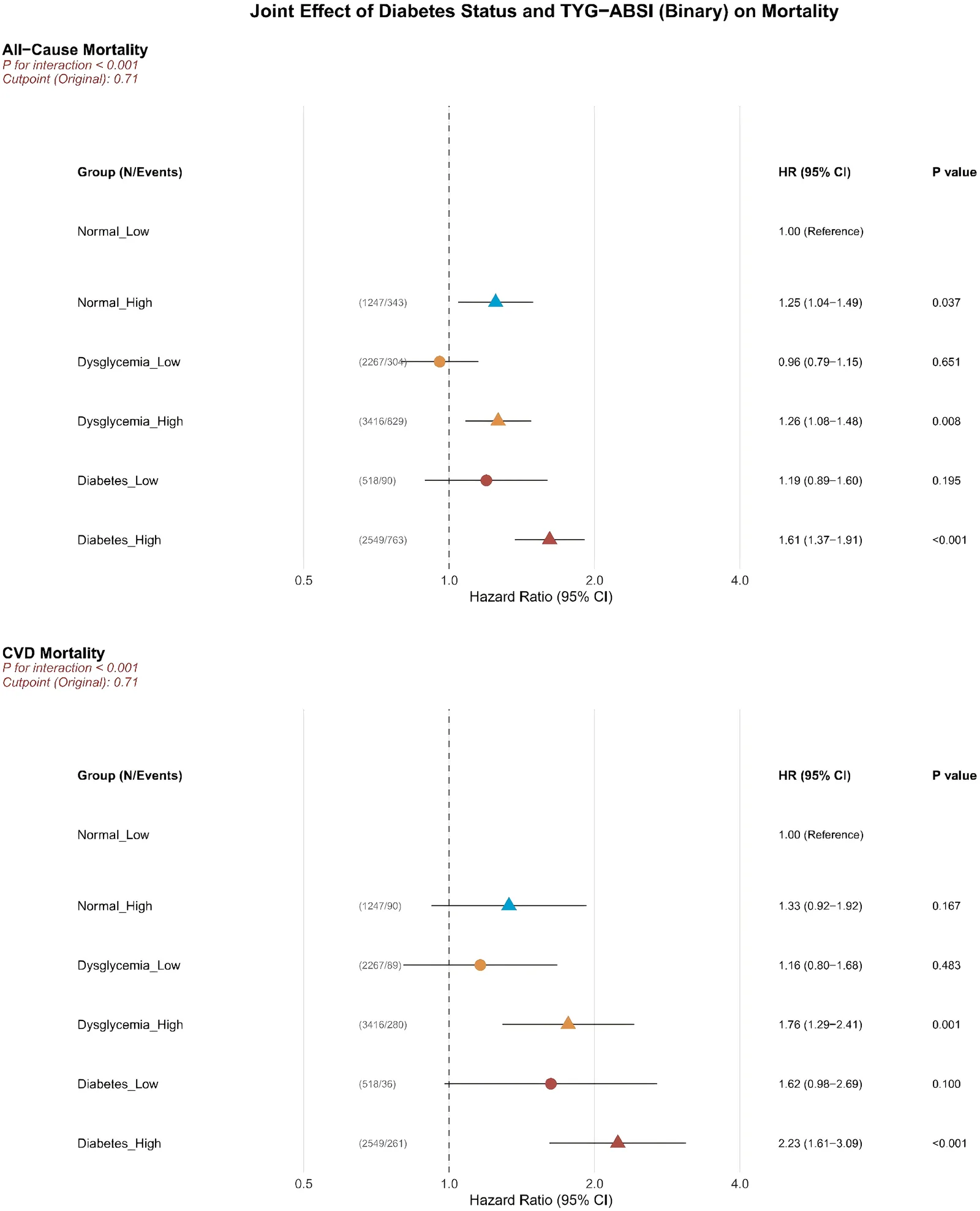
Forest plot of joint effects of diabetes status and TyG-ABSI on all-cause and cardiovascular disease mortality Adjusted hazard ratios (HRs) and 95% confidence intervals (CIs) were derived from Cox proportional hazards models. The reference group was participants with normal glucose and low TyG-ABSI levels (Normal_Low). Symbols represent adjusted HRs and horizontal lines represent 95% CIs. P values shown for individual groups compare each group with the Normal_Low reference group, and P for interaction assesses the interaction between glycemic status and TyG-ABSI category. The optimal cut-point for TyG-ABSI was determined using maximally selected rank statistics (*Z* score: −0.306, original value: 0.71). Models were adjusted for age, gender, race, education, poverty-income ratio, marital status, smoking, alcohol use, antihypertensive drug use, hypertension, congestive heart failure, coronary heart disease, angina, heart attack, stroke, kidney disease, and cancer. TyG-ABSI, triglyceride-glucose index combined with a body shape index; HR, hazard ratio; CI, confidence interval.

In the joint-effect analysis (Figure 4), participants were cross-classified by glycemic status (normal glucose metabolism, dysglycemia, and diabetes) and TyG-ABSI category, with the normal glucose/low TyG-ABSI group as the reference. A clear gradient emerged in the high TyG-ABSI stratum: compared with the reference group, all-cause mortality risk increased progressively with worsening glycemic status, reaching its maximum in diabetes with high TyG-ABSI (HR, 1.61; 95% CI, 1.37–1.91; *p* < 0.001), followed by dysglycemia with high TyG-ABSI (HR, 1.26; 95% CI, 1.08–1.48; *p* = 0.008) and normal glucose metabolism with high TyG-ABSI (HR, 1.25; 95% CI, 1.04–1.49; *p* = 0.037). For cardiovascular mortality, the excess risk was concentrated in hyperglycemic states when TyG-ABSI was high, with the strongest association again observed in diabetes with high TyG-ABSI (HR, 2.23; 95% CI, 1.61–3.09; *p* < 0.001), followed by dysglycemia with high TyG-ABSI (HR, 1.76; 95% CI, 1.29–2.41; *p* = 0.001). In contrast, across glycemic categories, the low TyG-ABSI groups did not show statistically significant elevations in mortality risk (all *p* > 0.05), supporting the interpretation that central obesity-related insulin resistance (captured by TyG-ABSI) amplifies the adverse mortality association of worsening glycemic status, with the largest hazard observed in diabetes.

### Comparison of TyG-derived indices in mortality prediction

**Figure 5:**
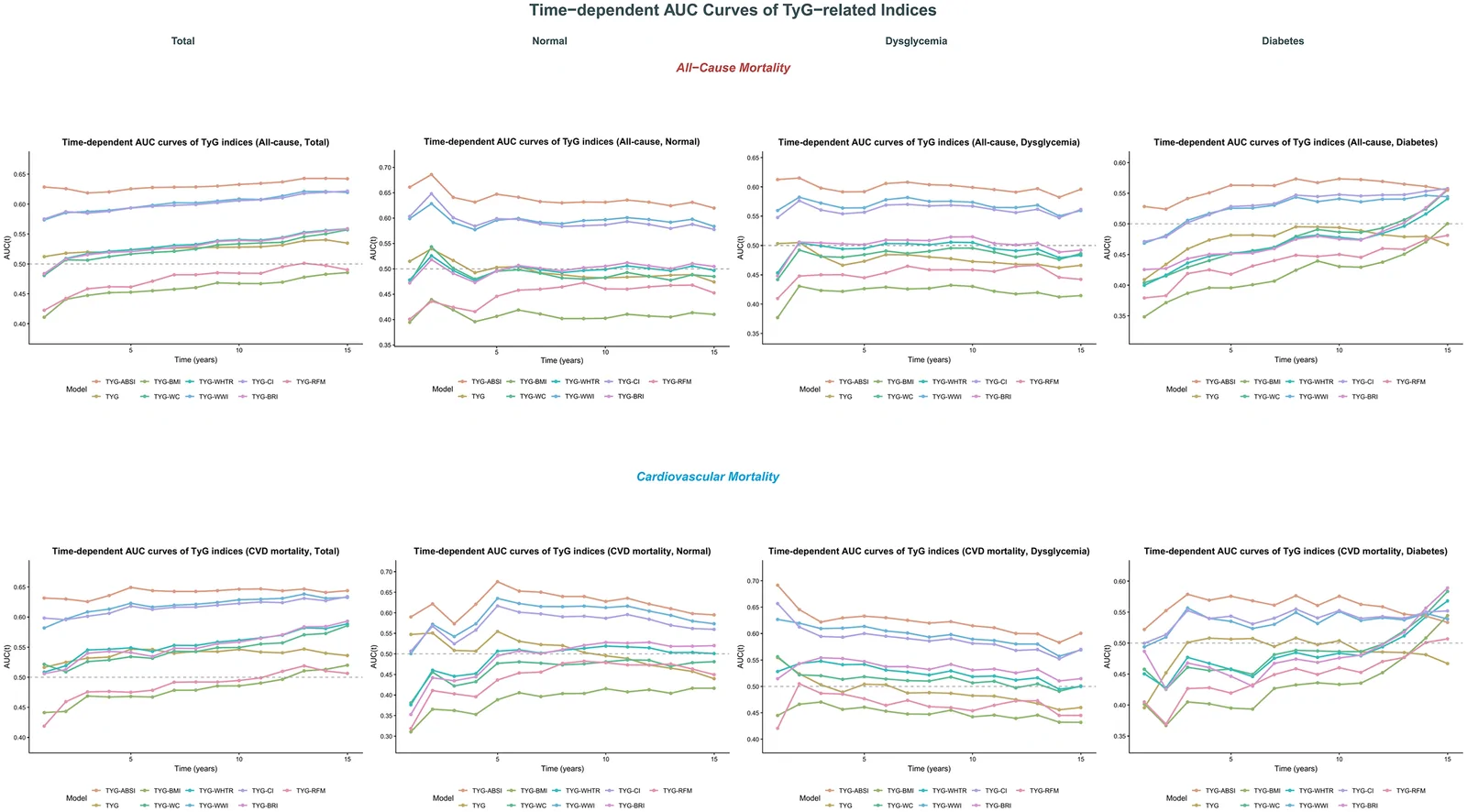
Time-dependent discriminative performance of TyG-related indices for predicting all-cause and cardiovascular mortality Time-dependent area under the receiver operating characteristic curve (AUC) analyses for TyG index and other TyG-related indices (TyG-BMI, TyG-WC, TyG-WHtR, TyG-WWI, TyG-ABSI, TyG-CI, TyG-BRI, and TyG-RFM) in predicting all-cause mortality (upper images) and cardiovascular mortality (lower images) over 15 years of follow-up. Curves depict the evolution of AUCs across follow-up time in the total population and across different glycemic categories (participants with normal glucose tolerance, dysglycemia, and diabetes). AUC, area under the receiver operating characteristic curve; TyG, triglyceride-glucose index; BMI, body mass index; WC, waist circumference; WHtR, waist-to-height ratio; WWI, weight-adjusted waist index; ABSI, a body shape index; CI, conicity index; BRI, body roundness index; RFM, relative fat mass.

Time-dependent ROC analysis showed that TyG-ABSI had generally higher and more stable AUC(t) trajectories than most other TyG-derived indices for both all-cause and cardiovascular mortality (Figure 5). In the overall cohort, TyG-ABSI maintained AUC values consistently above 0.60 throughout follow-up, with stable discrimination over time. When stratified by glucose metabolism status, TyG-ABSI achieved optimal discriminative ability in normoglycemic individuals (AUC, 0.65–0.70), followed by those with dysglycemia (AUC, 0.60–0.65). Although predictive accuracy attenuated among participants with diabetes, TyG-ABSI remained the most robust predictor relative to other TyG-derived indices. Detailed results for additional TyG-derived indicators are provided in the supplemental information.

### The incremental value of TyG and its derivative indices in risk prediction

Reclassification analyses indicated that TyG-ABSI provided significant incremental reclassification improvement over several alternative TyG-derived indices (Table S3). In the overall cohort, TyG-ABSI demonstrated significant improvement in risk reclassification when using conventional TyG as the reference model for both all-cause mortality (NRI, 0.203–0.237; IDI, 0.015–0.049; all *p* < 0.001) and cardiovascular mortality (NRI, 0.244–0.257; IDI, 0.008–0.023; all *p* < 0.001) across 5-, 10-, and 15-year follow-up. Similar advantages were observed when other composite indices served as reference models. When stratified by glucose metabolism status, TyG-ABSI retained significant reclassification improvement in normoglycemic and dysglycemic populations; however, its predictive superiority was substantially attenuated among individuals with diabetes, where most comparisons did not achieve statistical significance. Complete NRI and IDI results are detailed in the supplemental information and Table S3.

**Figure 6:**
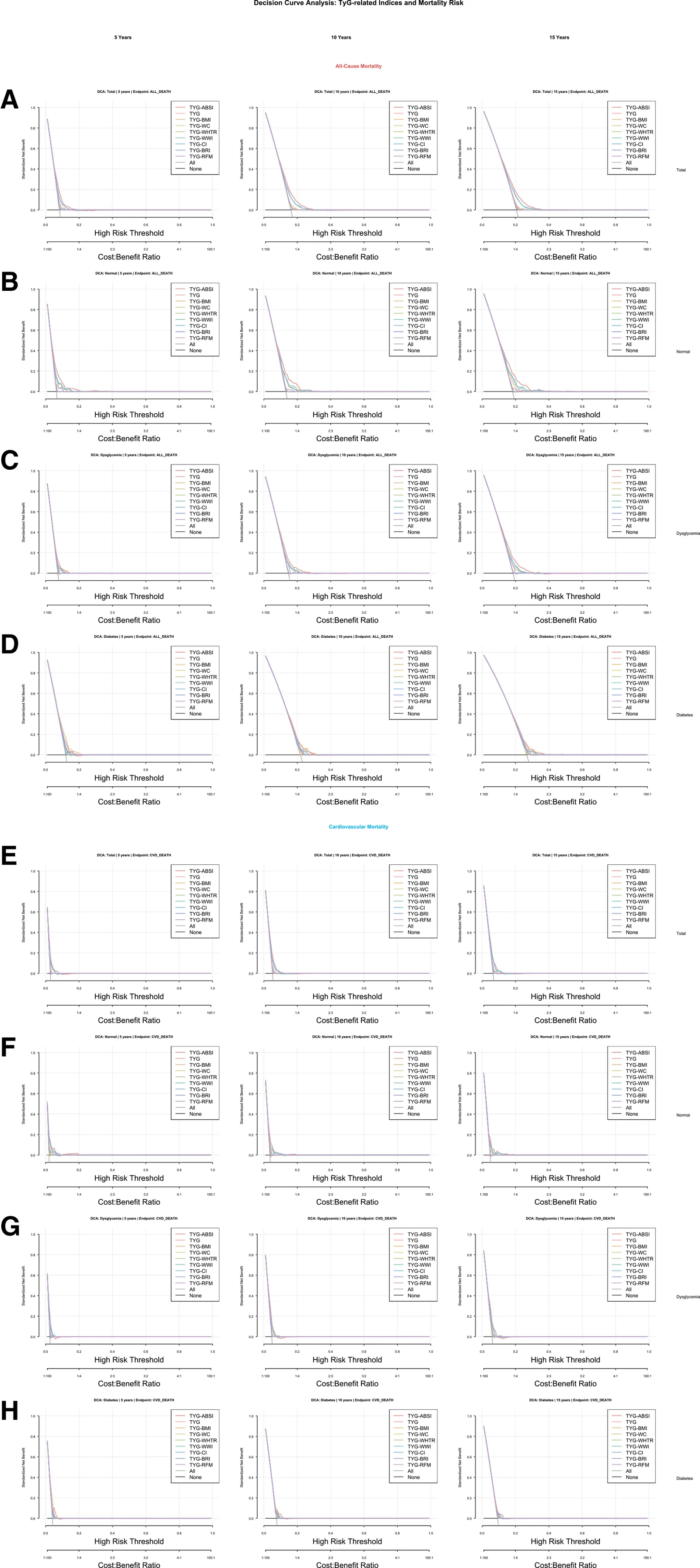
Decision curve analysis of TyG and its derivative indicators in predicting all-cause mortality and cardiovascular mortality Decision curve analysis (DCA) for the net clinical benefit of the TyG index and TyG-related indices (TyG-ABSI, TyG-BMI, TyG-WC, TyG-WHtR, TyG-WWI, TyG-CI, TyG-BRI, and TyG-RFM). (A–D) Decision curve analyses for all-cause mortality in the total population (A), participants with normal glucose tolerance (B), dysglycemia (C), and diabetes (D), respectively. (E–H) Decision curve analyses for cardiovascular mortality in the total population (E), participants with normal glucose tolerance (F), dysglycemia (G), and diabetes (H), respectively. Each panel presents results at 5-, 10-, and 15-year prediction horizons.

Decision curve analysis indicated that TyG-ABSI generally provided the highest net benefit among the evaluated TyG-derived indices for all-cause and cardiovascular mortality across 5-, 10-, and 15-year horizons (Figure 6). In the overall cohort, TyG-ABSI generally showed higher net benefit than the other TyG-derived indices across a wide range of threshold probabilities. A consistent finding across all glycemic strata was that TyG-ABSI maintained the highest net benefit relative to other indices. However, the magnitude of this advantage varied by metabolic status: in normoglycemic individuals, net-benefit differences among indices were modest with substantial curve overlap; in contrast, the superiority of TyG-ABSI became increasingly pronounced in dysglycemic and diabetic populations, particularly at midrange threshold probabilities and with longer follow-up durations. Detailed results for TyG and its derivative metrics are presented in Tables S1, S2, S3, S4, S5, S6, S7, S8, S9, S10, and S11.

### Life-table analysis and decomposition of survival differences based on TyG-derived indices

Using abridged period life tables, we estimated remaining life expectancy at age 45 years (e45) across tertiles of the triglyceride-glucose index (TyG) and TyG-derived indices (Table 4). Indices incorporating central adiposity and body-shape components (TyG-ABSI, TyG-CI, and TyG-WWI) showed the largest gradients in e45. For example, in the overall population, e45 decreased from 35.16 years in the lowest tertile (T1) of TyG-ABSI to 29.01 years in the highest tertile (T3), corresponding to a 6.15-year deficit (Δe45 = e45[T1] − e45[T3]). These findings suggest that central body-shape components capture prognostic information beyond glycemic and lipid measures alone.

**Table 4:** Life expectancy at age 45 years and years of life lost by tertiles of TyG-derived indices

TyG index	Population	T1 (years)	T3 (years)	ΔLE (years)
TyG	Total	33.7	30.3	+3.3
TyG	Normal	33.5	32.8	+0.7
TyG	dysglycemia	34.0	32.3	+1.7
TyG	diabetes	28.6	25.3	+3.4
TyG-BMI	total	32.8	30.6	+2.2
TyG-BMI	normal	32.5	34.9	−2.4
TyG-BMI	dysglycemia	33.9	33.0	+0.9
TyG-BMI	diabetes	27.6	25.2	+2.4
TyG-WC	total	33.7	29.7	+4.1
TyG-WC	normal	33.7	33.4	+0.3
TyG-WC	dysglycemia	34.4	31.1	+3.3
TyG-WC	diabetes	28.8	24.6	+4.2
TyG-WHtR	total	33.6	30.2	+3.4
TyG-WHtR	normal	33.5	33.3	+0.1
TyG-WHtR	dysglycemia	34.1	32.1	+2.0
TyG-WHtR	diabetes	28.8	25.4	+3.3
TyG-WWI	total	34.2	29.2	+4.9
TyG-WWI	normal	35.3	32.7	+2.7
TyG-WWI	dysglycemia	34.3	31.4	+2.9
TyG-WWI	diabetes	29.3	24.6	+4.7
TyG-ABSI	total	35.2	29.0	+6.1
TyG-ABSI	normal	35.7	31.8	+4.0
TyG-ABSI	dysglycemia	35.6	31.2	+4.4
TyG-ABSI	diabetes	29.8	24.7	+5.1
TyG-CI	total	34.8	29.1	+5.7
TyG-CI	normal	34.8	31.8	+3.0
TyG-CI	dysglycemia	35.3	31.5	+3.8
TyG-CI	diabetes	30.1	24.5	+5.6
TyG-BRI	total	33.3	30.2	+3.1
TyG-BRI	normal	33.4	33.1	+0.3
TyG-BRI	dysglycemia	34.3	31.8	+2.4
TyG-BRI	diabetes	27.9	25.1	+2.8
TyG-RFM	total	31.7	32.7	−1.0
TyG-RFM	normal	31.8	34.6	−2.8
TyG-RFM	dysglycemia	32.3	35.2	−2.9
TyG-RFM	diabetes	28.1	27.5	+0.6

T1, lowest tertile; T3, highest tertile; ΔLE, years of life lost calculated as T1 minus T3; TyG, triglyceride-glucose index; BMI, body mass index; WC, waist circumference; WHtR, waist-to-height ratio; WWI, weight-adjusted waist index; ABSI, a body shape index; CI, conicity index; BRI, body roundness index; RFM, relative fat mass.

Life expectancy at age 45 was calculated using abridged period life tables based on the Chiang method. Tertiles were defined using survey-weighted percentiles. Positive ΔLE values indicate that individuals in the highest tertile (T3) have shorter remaining life expectancy compared to those in the lowest tertile (T1); negative values indicate T3 has longer life expectancy. Total population includes all participants aged ≥45 years (*n* = 11,787).

Across glycemic strata (normoglycaemia, dysglycaemia, and diabetes), higher TyG and TyG-derived metrics were generally associated with shorter remaining life expectancy at e45. The magnitude of the e45 deficit varied by index. For example, the Δe45 gradient for TyG-ABSI was 3.97 years in the normoglycaemia group, 4.37 years in the dysglycaemia group, and 5.09 years in the diabetes group. Notably, within each stratum, central-adiposity-anchored composite indices consistently exhibited stronger discrimination than traditional obesity metrics, whereas several non-body-shape indices showed attenuated separation among participants with confirmed diabetes, consistent with risk saturation and the influence of clinical management.

**Figure 7:**
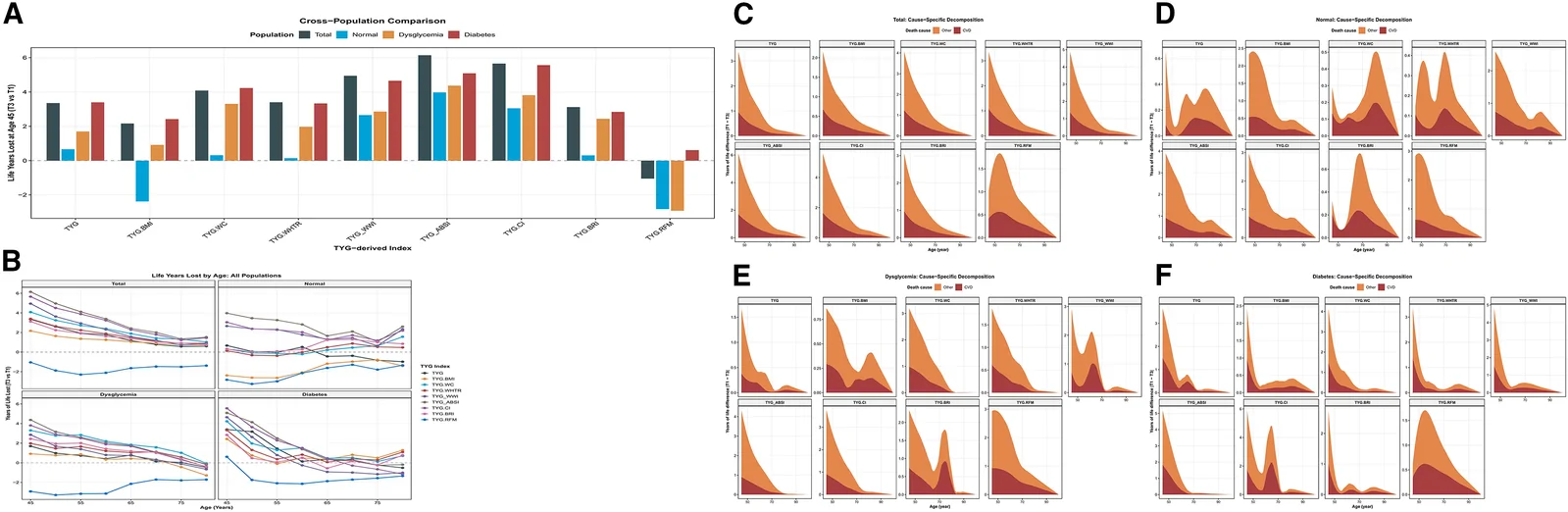
Differences in life expectancy and causes of death among individuals with different glycemic states based on tertile classification of TyG and its derived indices (A) Remaining life expectancy at age 45 comparing the highest (T3) versus lowest (T1) tertiles of TyG-derived indices across four populations. Positive values indicate shorter life expectancy in T3 compared to T1. (B) Age-specific patterns of life years lost (T3 vs. T1) from age 45 to 80 years, stratified by glycemic status. (C–F) Cause-specific decomposition of life expectancy differences between T1 and T3 tertiles, showing the contributions of cardiovascular disease (CVD, dark red) and other causes of death (orange) to the total life expectancy gap. C: Total population; D: Normal glycemic status; E: Dysglycemia; F: Diabetes. Life expectancy was estimated using abridged period life tables based on the Chiang method. Decomposition analysis was performed using the Arriaga method to partition life expectancy differences by cause of death. TyG, triglyceride-glucose index; BMI, body mass index; WC, waist circumference; WHtR, waist-to-height ratio; WWI, weight-adjusted waist index; ABSI, a body shape index; CI, conicity index; BRI, body roundness index; RFM, relative fat mass; CVD, cardiovascular disease; T1/T3, lowest/highest tertile.

Decomposition analyses indicated that the longevity gap associated with higher TyG-ABSI was driven predominantly by non-cardiovascular mortality (overall: 4.44 years; 72.3%), with cardiovascular mortality contributing a smaller but meaningful fraction (overall: 1.70 years; 27.7%) and a larger proportional share as glycemic status worsened (CVD share: 23.4% in normoglycemia, 21.0% in dysglycaemia, 34.7% in diabetes) (Figure 7).

### Population-attributable fraction analysis of TyG-related indices for mortality

**Figure 8:**
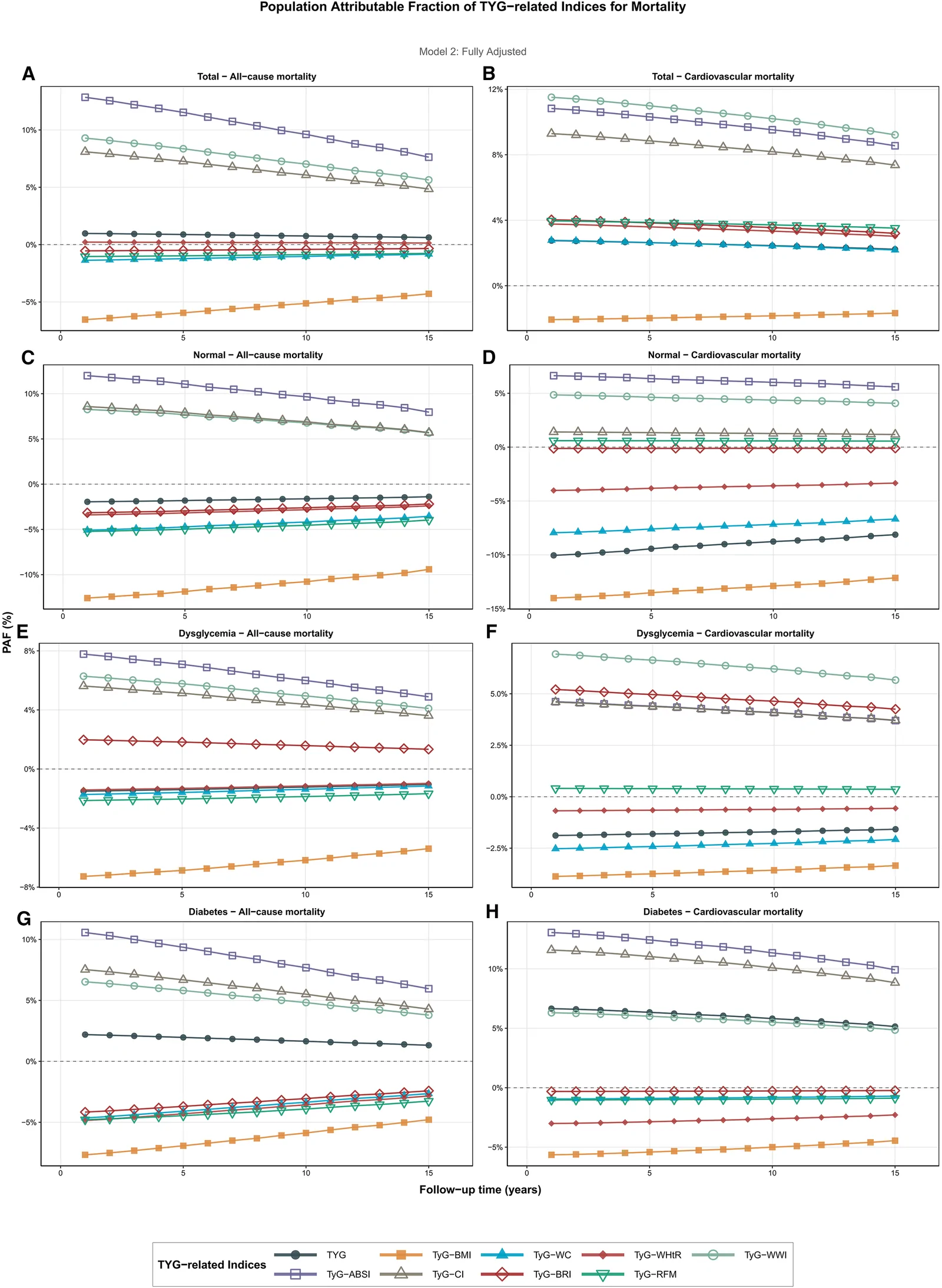
Time-dependent population-attributable fractions of mortality associated with TyG-related indices Time-dependent population attributable fractions (PAFs) of all-cause mortality (A, C, E, and G) and cardiovascular mortality (B, D, F, and H) associated with TyG index and TyG-related parameters (TyG-BMI, TyG-WC, TyG-WHtR, TyG-WWI, TyG-ABSI, TyG-CI, TyG-BRI, and TyG-RFM) over 15 years of follow-up. The analyses were stratified by glycemic status: total population (A, B), participants with normal glucose tolerance (C and D), dysglycemia (E and F), and diabetes (G and H). Model 2 (Fully adjusted model): Adjusted for age, gender, race, education, PIR, marital status, smoking, alcohol use, antihypertensive drug use, hypertension, heart failure, heart disease, angina, heart attack, stroke, kidney failure, and cancer. PAF, population-attributable fraction; TyG, triglyceride-glucose index; BMI, body mass index; WC, waist circumference; WHtR, waist-to-height ratio; WWI, weight-adjusted waist index; ABSI, a body shape index; CI, conicity index; BRI, body roundness index; RFM, relative fat mass; PIR, poverty-income ratio.

PAF analysis yielded the largest estimated population-attributable fraction for TyG-ABSI among the evaluated TyG-derived indices across the overall cohort (Figure 8). For all-cause mortality, the PAF for TyG-ABSI decreased from 12.8% at baseline to 7.6% at 15 years; for cardiovascular mortality, corresponding values declined from 10.8% to 8.5%. A consistent finding across all glycemic strata was that TyG-ABSI maintained the highest PAF relative to other indices at all time points. However, notable heterogeneity emerged across subgroups: for all-cause mortality, the attributable burden was greatest in normoglycemic individuals (PAF, 12.0%–8.0%), whereas for cardiovascular mortality, diabetic individuals demonstrated the highest PAF (13.0%–9.9%). The dysglycemia subgroup consistently showed the smallest attributable fraction for both outcomes. Across all populations and outcomes, the attributable burden was strongest early in follow-up and attenuated over time. Detailed attribution scores for all TyG-derived indicators are provided in the supplemental information.

### Mediation and network analyses of inflammatory indices in the association between TyG-ABSI and mortality

Exploratory mediation analyses suggested that systemic inflammatory indices statistically mediated a modest proportion of the TyG-ABSI-mortality association, with systemic inflammatory response index (SIRI) showing the largest estimated mediation proportion (Figures S2–S4). In the overall population, SIRI mediated 5.8% of the TyG-ABSI effect on all-cause mortality and 4.8% on cardiovascular mortality (both *p* < 0.001), followed by systemic immune-inflammation index (SII) (2.8% and 2.4%, respectively) and aggregate index of systemic inflammation (AISI) (1.4% and 1.3%, respectively). Stratified analyses revealed substantial heterogeneity across glycemic subgroups. The mediating effects were most pronounced in the dysglycemia subgroup, where SIRI mediated 16.9% and 14.6% of all-cause and cardiovascular mortality, respectively. Normoglycemic individuals demonstrated intermediate mediation proportions (SIRI: 8.7% for all-cause mortality), while diabetic individuals showed the weakest and often non-significant mediating effects. The mixed graphical model offers a complementary perspective, demonstrating that within the overall population, the SIRI exhibited the strongest edge weights both for the path from TyG-ABSI to the mediator (path a, weight = 0.11) and for the path from the mediator to the outcome (path b, weight = 0.35). Notably, the Path b weight for SIRI was highest in the dysglycemia subgroup (0.39), consistent with the enhanced mediation proportion observed in this metabolically vulnerable population.

### Subgroup analysis of TyG-ABSI association with mortality risk

Subgroup analyses revealed consistent associations between TyG-ABSI and mortality across sexes (all *P* for interaction >0.05; Figure S5). However, significant age-related interactions were observed (*P* for interaction <0.001 for both all-cause and CVD mortality), with stronger associations in younger individuals. This age interaction remained significant for all-cause mortality among normoglycemic participants (*P* for interaction = 0.038) but was non-significant in those with dysglycemia or diabetes (*P* for interaction >0.05). No significant age interactions were identified for CVD mortality within any glycemic stratum.

### Sensitivity analyses

Proportional hazards assumptions were evaluated for each TyG-related index across glycemic strata and endpoints; several indices showed evidence of non-proportionality in selected strata (Figure S6). We therefore fitted time-varying coefficient Cox models including an exposure×log(follow-up time) interaction; these analyses indicated no evidence of time-dependent effects for TyG-ABSI across all strata and both endpoints (all interaction *p* ≥ 0.05) (Table S4).

Sensitivity analyses further supported the robustness of our primary findings. After Benjamini-Hochberg false discovery rate (FDR) correction (Figure S7), TyG-ABSI remained significantly associated with all-cause mortality across all glycemic strata (all *p* < 0.001) and with cardiovascular mortality in the total population and glycemic subgroups, except among normoglycemic participants (all *p* < 0.05). E-value analysis (Table S5) further assessed the potential impact of unmeasured confounding. In the total population, the E-values for TyG-ABSI were 1.66 for all-cause mortality and 1.75 for cardiovascular mortality per SD increase, increasing to 2.33 and 2.57, respectively, for comparisons of the highest versus lowest tertiles. Across glycemic subgroups, the point-estimate E-values for the per-SD association of TyG-ABSI with all-cause mortality ranged from 1.47 to 1.67, whereas those for cardiovascular mortality ranged from 1.42 to 1.61. The corresponding E-values for the highest versus lowest tertile ranged from 2.00 to 2.12 for all-cause mortality and from 1.33 to 2.45 for cardiovascular mortality. These findings indicate that unmeasured confounding of at least the magnitude represented by these E-values would be required to fully explain away the observed associations. FDR-corrected results and E-values for all TyG-derived metrics are provided in Figure S7 and Table S5, respectively.

Second, to address potential reverse causality, we reanalyzed the data after excluding deaths occurring within the first 2 years of follow-up. The associations of TyG-ABSI with both all-cause mortality (HR = 1.192; 95% CI: 1.142–1.245; *p* < 0.001) and cardiovascular mortality (HR = 1.231; 95% CI: 1.141–1.328; *p* < 0.001) remained statistically significant and were broadly consistent with the primary analyses (Table S6).

Third, Fine-Gray competing risks regression confirmed the robustness of associations after accounting for non-cardiovascular mortality as a competing event (Table S7). In the overall population, each SD increase in TyG-ABSI was associated with a 16% higher risk of cardiovascular mortality (subdistribution HR, 1.16; 95% CI, 1.07–1.25; *p* < 0.001), with a significant dose-response relationship across tertiles (*P* for trend = 0.003). Stratified analyses revealed notable heterogeneity across glycemic subgroups. No significant association was observed among normoglycemic individuals (HR, 1.02; 95% CI, 0.84–1.23; *p* = 0.850). In contrast, the association became evident with worsening glucose metabolism: in the dysglycemia subgroup, a significant trend across tertiles was observed (*P* for trend = 0.015), while among diabetic individuals, each SD increase in TyG-ABSI conferred a 15% higher cardiovascular mortality risk (HR, 1.15; 95% CI, 1.02–1.30; *p* = 0.022). These findings suggest that the association between TyG-ABSI and cardiovascular mortality is more pronounced in populations with impaired glucose metabolism.

Fourth, to assess robustness under alternative adjustment and standardization approaches, we employed generalized propensity score (GPS) weighting and the G-formula. GPS-weighted analysis confirmed the association between TyG-ABSI and all-cause mortality (HR = 1.238; 95% CI: 1.186–1.293; *p* < 0.001) and cardiovascular mortality (HR = 1.269; 95% CI: 1.179–1.366; *p* < 0.001), consistent with the primary analysis. The G-formula analysis similarly confirmed the significant association between TyG-ABSI and mortality risk (RR for all-cause mortality = 1.489; RR for cardiovascular mortality = 1.655; both *p* < 0.001) (Table S8). In addition, IPTW analyses (Table S9) yielded concordant results (per SD: all-cause HR = 1.219, 95% CI: 1.176–1.264; cardiovascular HR = 1.250, 95% CI: 1.173–1.333; both *p* < 0.001), with a similar gradient in tertile analyses (T3 vs. T1: all-cause HR = 1.478, 95% CI: 1.345–1.625; cardiovascular HR = 1.576, 95% CI: 1.327–1.871; both *p* < 0.001).

We then turned to the CHARLS cohort (*n* = 12,915) for external validation (Table S10). Here, each SD increment in TyG-ABSI translated to a 10% increase in all-cause mortality (HR = 1.10, 95% CI: 1.07–1.14), with the top tertile facing 43% higher risk than the bottom. Notably, this association held across all three glycemic strata—normoglycemia (HR 1.08), prediabetes (HR = 1.12), and diabetes (HR = 1.11)—though effect sizes were somewhat attenuated compared with NHANES. Baseline TyG-ABSI showed relatively stable and generally higher AUC(t) trajectories for all-cause mortality across glycemic strata, although absolute discrimination was modest and differences from TyG-WWI and TyG-CI were limited at several time points (revised Figure S9). Thus, CHARLS supported the comparative stability of TyG-ABSI rather than its use as a standalone clinical prediction marker. TyG-WWI and TyG-CI tracked similar patterns in CHARLS as in NHANES, suggesting these findings are not artifacts of a single cohort or population structure.

To address potential instability of period life table estimates due to sparse cells in finer age strata, we repeated the analyses using broader 10-year age bands. Results were consistent for TyG-ABSI across glycemic strata: life expectancy at age 45 years was 35.2 years in T1 versus 28.9 years in T3 (ΔLE 6.2 years) in the total population, 35.8 versus 31.6 (ΔLE 4.2 years) in normoglycemia, 35.5 versus 31.2 (ΔLE 4.2 years) in dysglycaemia, and 29.6 versus 24.2 (ΔLE 5.4 years) in diabetes, indicating a broadly similar gradient with a larger absolute deficit in diabetes (Table S11).

## Discussion

In this systematic evaluation across two large, independent populations, TyG-ABSI showed the most consistent comparative prognostic performance among the nine evaluated TyG-related indices. Unlike traditional metrics based on general adiposity (such as TyG-BMI), which showed inconsistent associations, TyG-ABSI demonstrated robust, linear risk gradients and optimal predictive utility regarding discrimination and reclassification. The prognostic power of TyG-ABSI varied by metabolic status: while predictive of all-cause mortality across all strata, its association with cardiovascular death was most pronounced in individuals with dysglycemia and diabetes. Notably, in the joint-effect analysis, low TyG-ABSI groups were not associated with statistically significant excess mortality relative to the normal-glucose/low TyG-ABSI reference group. The association between TyG-ABSI and mortality is partially mediated by systemic inflammation. Sensitivity analyses supported the robustness of these associations, positioning TyG-ABSI as a readily accessible candidate risk marker for higher-risk central-adiposity phenotypes.

Previous large-scale cohorts, such as the ChinaHEART project, have established the TyG index as a robust predictor of mortality.^11^ However, subsequent investigations suggest that combining TyG with anthropometric markers of adiposity enhances this predictive power. Studies in US and UK populations have shown that indices such as TyG-WC and TyG-WHtR independently predict mortality and outperform the TyG index alone, particularly in high-risk groups with metabolic syndrome or hypertension.^22^^,^^23^

This advantage was reinforced by a recent meta-analysis confirming the consistent association of TyG-WC with cardiovascular outcomes.^24^ Against this background, our study addresses a critical gap by conducting a head-to-head comparison of a comprehensive panel of nine indices—incorporating body-shape measures such as TyG-ABSI—within the same population. Furthermore, we systematically evaluate their prognostic utility across distinct glycemic strata in both US and Chinese cohorts. Our findings demonstrate that TyG-ABSI, by capturing central body shape, offers superior discrimination for all-cause and cardiovascular mortality compared to traditional TyG-derived metrics, specifically across the spectrum of glucose metabolism.

In our multivariable-adjusted analyses, indices integrating central body shape—specifically TyG-ABSI, TyG-WWI, and TyG-CI—demonstrated robust, linear associations with mortality, contrasting sharply with the inverse or weak relationships observed for mass-based metrics such as TyG-BMI. This prognostic superiority was underscored by joint exposure analyses, which revealed a steep risk gradient: mortality hazards were maximal in individuals with concurrent diabetes and elevated TyG-ABSI, whereas maintaining low TyG-ABSI levels mitigated excess risk even in the presence of dysglycemia. These findings are consistent with TyG-ABSI integrating information related to insulin resistance and central adiposity; however, the underlying biological pathways were not directly measured. Insulin resistance drives hyperglycemia, atherogenic dyslipidemia, and endothelial dysfunction,^25^^,^^26^^,^^27^^,^^28^ perpetuating a cycle of oxidative stress and systemic inflammation.^29^^,^^30^ While the TyG index reflects this metabolic dysfunction,^31^^,^^32^ its combination with ABSI specifically isolates visceral fat—a depot uniquely prone to macrophage infiltration and pro-inflammatory adipokine secretion.^33^^,^^34^^,^^35^ Unlike BMI, which acts as a crude proxy for adiposity and may conflate lean mass with fat—potentially explaining the “obesity paradox” observed with TyG-BMI^36^—central measures such as ABSI more accurately reflect metabolic risk.^37^ Thus, TyG-ABSI likely outperforms other indices because it integrates two converging pathological pathways—systemic insulin resistance and central lipotoxicity—into a single, powerful prognostic marker.^38^

In addition, prior experimental and translational studies have linked endothelial injury and cardiovascular damage to mitochondrial abnormalities, altered mitochondrial quality control, and mitochondria-endoplasmic-reticulum stress responses.^39^^,^^40^^,^^41^^,^^42^^,^^43^^,^^44^ These studies provide biological plausibility for the possibility that insulin-resistance-related phenotypes may be accompanied by vascular and myocardial stress responses, particularly under hyperglycemic or adiposity-related metabolic stress. Related work has also implicated mechanosensitive/cytoskeletal remodeling, redox imbalance, and regulated cell-death processes in cardiovascular injury models.^42^^,^^45^^,^^46^^,^^47^^,^^48^^,^^49^^,^^50^^,^^51^^,^^52^^,^^53^ However, these pathways were not directly assessed in the present NHANES or CHARLS cohorts. Mitochondrial function, oxidative stress, endothelial function, endoplasmic-reticulum stress, ferroptosis, pyroptosis, and related molecular markers were not measured. Therefore, these mechanisms should be interpreted as hypothesis-generating explanations based on external literature rather than mechanistic evidence directly established by our data.

To further explore potential pathway-related explanations within the limits of the available data, we evaluated circulating inflammatory indices as statistical mediators of the association between TyG-ABSI and mortality. In these exploratory mediation analyses, SIRI, SII, and AISI mediated a modest proportion of the total association, with SIRI showing the largest mediating proportion, particularly among participants with dysglycaemia. These findings are consistent with prior evidence linking insulin resistance, adiposity-related metabolic dysfunction, oxidative stress, endothelial dysfunction, and low-grade inflammation with adverse cardiometabolic outcomes, as well as with cohort studies showing prognostic associations of SII, SIRI, and related systemic inflammatory indices with all-cause and cardiovascular mortality.^54^^,^^55^^,^^56^^,^^57^ However, SII, SIRI, and AISI are nonspecific, blood-cell-derived inflammatory indices and do not directly measure inflammatory pathways, cytokine activity, endothelial function, oxidative stress, or mitochondrial biology. Therefore, the mediation findings should be interpreted as hypothesis-generating and should not be considered evidence of a causal inflammatory mechanism. We also observed age-related heterogeneity in the association between TyG-ABSI and mortality, with stronger relative associations in younger participants. This pattern may reflect differences in biological aging, survivor selection, competing risks, or duration and severity of metabolic exposure, and is broadly consistent with prior work suggesting age-dependent associations between metabolic risk markers and mortality.^58^^,^^59^^,^^60^ Together with prior evidence on abdominal obesity and metabolic syndrome, these observations suggest that TyG-ABSI may help identify a higher-risk central-adiposity-related insulin-resistance phenotype, while supporting risk stratification rather than establishing TyG-ABSI as a therapeutic target.^61^

The primary strength of this study lies in its comparative prognostic design, the use of a nationally representative NHANES sample with long-term mortality follow-up, and external evaluation in the CHARLS cohort. Rather than testing a mechanistic model, we compared nine TyG-derived indices within a unified analytic framework and across clinically relevant glycemic strata. The joint-effect, life-table, population-attributable fraction, reclassification, and decision-curve analyses provided complementary perspectives on relative risk, absolute mortality burden, and potential risk-stratification utility. The broadly consistent findings across sensitivity analyses and the CHARLS cohort support the stability and generalizability of the observed associations, while future mechanistic and interventional studies are needed to determine whether TyG-ABSI reflects modifiable biological pathways or improves preventive decision-making.

### Clinical implications

TyG-ABSI can be calculated using routinely available clinical variables, including waist circumference, height, weight, fasting triglycerides, and fasting glucose. In this study, TyG-ABSI was calculated as TyG × [waist circumference (m)/(BMIˆ(2/3) × height (m)ˆ(1/2))], where TyG = ln[(fasting triglycerides × fasting glucose)/2] and BMI = weight/height^2^. Based on the maximally selected rank statistic, a TyG-ABSI value of approximately 0.71 identified a higher-risk group in the present cohort. Clinically, individuals above this threshold may warrant closer cardiometabolic risk assessment, lifestyle and weight-distribution management, and periodic follow-up of glycemic, lipid, blood pressure, and vascular risk profiles. However, this cut-off should be considered exploratory and requires external clinical validation before being used as a definitive decision threshold.

### Conclusion

In NHANES, TyG-ABSI showed the most consistent comparative associations and predictive performance for long-term mortality among the evaluated TyG-related indices, while CHARLS externally supported its association with all-cause mortality with modest discrimination. Associations varied by glycemic status: all-cause mortality associations were observed across strata, whereas cardiovascular associations were more apparent in dysglycemia and diabetes. Joint analysis identified diabetes with high TyG-ABSI as the highest-risk phenotype, whereas low TyG-ABSI groups were not associated with statistically significant excess risk across glycemic categories. Notably, life table analysis demonstrated that TyG metrics incorporating central adiposity—specifically TyG-ABSI—were strongly associated with significant loss of life expectancy. These losses were driven primarily by non-cardiovascular causes, though cardiovascular mortality accounted for a substantial proportion among patients with diabetes. Exploratory analyses suggested modest statistical mediation by systemic inflammatory indices, particularly SIRI.

Collectively, our findings underscore the necessity of concurrently managing central obesity and insulin resistance. As a routinely calculable candidate risk marker, TyG-ABSI may provide additional risk-stratification information; however, its clinical utility and decision thresholds require prospective external validation before routine use.

### Limitations of the study

Several limitations merit consideration. First, the observational design precludes definitive causal inference; despite extensive adjustment and E-value sensitivity analysis, residual confounding remains possible. Critically, we currently lack longitudinal interventional evidence demonstrating that therapeutic reduction of TyG-ABSI translates into improved survival—a pivotal evidence gap that must be bridged to establish its feasibility as a modifiable treatment target rather than solely a risk marker. In addition, because NHANES fasting biomarkers were unavailable for a subset of age-eligible participants, complete-case restriction may introduce selection bias. Excluded participants differed in fasting glucose, triglycerides, TyG index, and some TyG-derived indices, suggesting that exposure distributions may not be fully comparable. Fasting subsample weights and sensitivity analyses supported robustness within the analytic sample but cannot eliminate this concern or fully ensure generalizability. Second, exposure and covariate assessments were limited to baseline, preventing us from accounting for temporal trajectories in metabolic status or lifestyle, which may introduce regression dilution bias. Given the long follow-up, proportional hazards assumptions were not uniformly satisfied for all indices; time-varying coefficient analyses were therefore performed, and Cox estimates should be interpreted as average associations over follow-up. Third, in Model 2, adjusting for common cardiovascular diseases such as heart failure, coronary heart disease, myocardial infarction, and stroke may attenuate effect estimates—if these conditions partially exist within the causal pathway linking insulin resistance to mortality. Therefore, Model 2 should be regarded as a conservative setting rather than an exact estimate of the overall effect. Fourth, the reliance on self-reported covariates and surrogate markers rather than direct measures of insulin resistance (e.g., clamp techniques) or visceral adiposity limits mechanistic precision. Fifth, given the potential for sample sparsity in advanced age strata to introduce stochastic variation in estimated mortality rates and life expectancy projections, we interpret the life table results as descriptive, supplementary evidence to contextualize the long-term mortality burden, rather than as a standalone inferential framework. Finally, the optimal TyG-ABSI cutoff value was data-driven, and our mediation analyses should be regarded as hypothesis-generating findings that warrant validation in future prospective and interventional studies. To advance toward precision medicine, future research must validate these thresholds in diverse populations and determine whether integrating TyG-ABSI into clinical decision algorithms yields superior preventive outcomes compared to managing glucose or obesity in isolation.

## Resource availability

### Lead contact

Further information and requests for resources should be directed to the Lead Contact, Zhonghua Sun (sunzhonghua20@163.com).

### Materials availability

This study did not generate new unique reagents or materials.

### Data and code availability

NHANES data are publicly available from the National Center for Health Statistics through the links provided in the key resources table. CHARLS data are available to registered users upon application and approval by the CHARLS research team and are subject to the CHARLS data-use agreement. The R code supporting the analyses reported in this paper has been deposited at Zenodo: https://doi.org/10.5281/zenodo.21739285. Access to the code files is restricted and may be requested through Zenodo or from the lead contact. Requests will be considered for legitimate academic research purposes. Any additional information required to reanalyze the data reported in this paper is available from the lead contact upon reasonable request.

## Acknowledgments

We thank all participants and investigators involved in the collection and organization of the NHANES and CHARLS data. This work was supported by the “2025 Hefei Municipal Health Science and Technology Projects” of the Hefei Municipal Health Commission (Hwk2025yb010) and the Science and Technology Development Fund of 10.13039/501100007289Nanjing Medical University (2017NJMU028). The funders had no role in the study design, data collection and analysis, decision to publish, or preparation of the manuscript.

## Author contributions

Y.T. and Z.Y. conducted the methodological design. W.W., H.W., S.W., and L.L. conducted the data collation and analysis work. Y.T. and Y.X. drafted the manuscript. J.W. and Z.S. worked on revising and conceptualizing the first draft. All authors approved the submitted version of the manuscript.

## Declaration of interests

The authors declare no competing interests.

## STAR★Methods

### Key resources table

**Table:** 

REAGENT or RESOURCE	SOURCE	IDENTIFIER
**Deposited data**		

National Health and Nutrition Examination Survey (NHANES)	Centers for Disease Control and Prevention	https://www.cdc.gov/nchs/nhanes/
NHANES Public-Use Linked Mortality File	National Center for Health Statistics	https://www.cdc.gov/nchs/data-linkage/mortality-public.htm
China Health and Retirement Longitudinal Study (CHARLS)	Peking University	http://charls.pku.edu.cn/en/
R code supporting the analyses	This paper	Zenodo: https://doi.org/10.5281/zenodo.21739285

**Software and algorithms**		

R statistical software	R Foundation for Statistical Computing	Version 4.3.0

### Experimental model and study participant details

#### Study design and population

We analysed NHANES, a nationally representative survey of the US civilian, noninstitutionalized population using stratified, multistage probability sampling.^62^^,^^63^ We pooled 10 consecutive cycles (1999–2018; *n* = 101,316). We excluded participants aged <45 years (*n* = 69,740) and those missing fasting glucose (*n* = 18,911), triglycerides (*n* = 206), diabetes status (*n* = 7), mortality linkage (*n* = 20), waist circumference (*n* = 617), or prespecified covariates (*n* = 28), leaving 11,787 individuals. Participants were classified as normoglycemia (*n* = 3,037), dysglycemia (*n* = 5,683), or diabetes (*n* = 3,067) (Figure 1). External validation used baseline data from the 2011 and 2015 waves of the CHARLS; details are provided in the supplemental information. The selection process for the CHARLS validation cohort is shown in Figure S8.

The NHANES 1999–2018 survey protocols were approved by the National Center for Health Statistics Ethics Review Board under Protocol Nos. 98-12, 2005-06, 2011-17, and 2018-01. The CHARLS protocol was approved by the Institutional Review Board of Peking University (IRB00001052-11015). All participants provided written informed consent before participation. The present study involved secondary analyses of de-identified data and was conducted in accordance with the Declaration of Helsinki.

### Method details

#### Definition of insulin resistance indicators

We employed the TyG index as an established proxy measure for quantifying insulin resistance.^6^ To enhance predictive capacity, we constructed composite indicators by combining the TyG index with various obesity parameters that reflect adiposity distribution and body composition characteristics. Detailed calculation formulas for all 9 TyG-related indices are provided in the supplemental information.

#### Definition of glycemic status

Glycemic status was defined using American Diabetes Association criteria adapted to NHANES measures.^64^^,^^65^ Diabetes was defined by fasting plasma glucose ≥126 mg/dL, glycated hemoglobin (HbA1c) ≥6.5%, or self-reported clinician diagnosis. Dysglycemia was defined by fasting plasma glucose 100–125 mg/dL or HbA1c 5.7–6.5%. Normal glycemia required fasting plasma glucose <100 mg/dL and HbA1c <5.7%, with no history of diabetes diagnosis or treatment.

#### Determination of mortality

Outcomes were all-cause and cardiovascular mortality.^63^ Vital status was ascertained via linkage to the NHANES Public-Use Linked Mortality File, with follow-up through Dec 31, 2019. Causes of death were coded using International Classification of Diseases, Tenth Revision (ICD-10). Cardiovascular mortality included deaths from heart disease (I00–I09, I11, I13, I20–I51) or cerebrovascular disease (I60–I69); all deaths contributed to the all-cause endpoint.

#### Covariates

Multivariable models adjusted for demographic, behavioural, and clinical factors. Demographic covariates included age (continuous), sex, race/ethnicity (Mexican American, Other Hispanic, Non-Hispanic White, Non-Hispanic Black, and Other Race including multi-racial), education (less than 9th grade; 9th–11th grade including 12th grade without diploma; high school graduate/GED; some college/associate degree; college graduate or above), poverty–income ratio (continuous), and marital status (married/living with partner vs separated/divorced/widowed/never married). Behavioural covariates were smoking status (never vs former) and alcohol use (current drinker vs non-drinker). Clinical covariates included antihypertensive medication use and self-reported physician-diagnosed hypertension, congestive heart failure, coronary heart disease, angina pectoris, myocardial infarction, stroke, chronic kidney disease, and cancer (all coded as yes/no).^62^

### Quantification and statistical analysis

#### Statistical analysis

All analyses incorporated NHANES survey weights to obtain nationally representative estimates. Baseline characteristics were summarised as weighted means (SD) or weighted frequencies (percentages); groups were compared using weighted analysis of variance (ANOVA) or χ^2^ tests. Proportional hazards were evaluated using Schoenfeld residuals; where violated, time-varying Cox models (exposure×log[time]) were used as sensitivity analyses. For hazard ratios reported per SD increase, SDs were computed in the overall analytic sample for total-population analyses and within each glycaemic stratum for stratified analyses. Associations of each TyG-related index with all-cause and cardiovascular mortality were examined using survey-weighted Cox proportional hazards models: a crude model; Model 1, which adjusted for demographic and behavioral covariates (age, sex, race/ethnicity, education, poverty–income ratio, marital status, smoking, and alcohol use) and was intended to estimate the overall association under a standard confounding set; and Model 2, which further adjusted for baseline clinical conditions and medication use (antihypertensive medication use, hypertension, heart failure, coronary heart disease/angina, myocardial infarction, stroke, chronic kidney disease, and cancer) to provide a more conservative estimate conditioned on baseline comorbidity burden and to assess whether TyG-related indices retained prognostic associations beyond established clinical risk factors. Dose–response relationships were assessed with restricted cubic splines (four knots). Joint associations of glycaemic status and TyG-ABSI were evaluated using six categories (normal/dysglycaemia/diabetes × low/high TyG-ABSI), with cut-points derived from maximally selected rank statistics; Kaplan–Meier curves (KM) and log-rank tests compared survival across categories. Predictive performance was assessed by time-dependent ROC analyses at 5, 10, and 15 years using the cumulative/dynamic definition with inverse probability of censoring weighting. These analyses used baseline TyG-related indices as fixed predictors and did not represent dynamically updated time-varying prediction models. The incremental prognostic value of TyG-ABSI was quantified using the continuous (category-free) NRI and IDI, while clinical utility was evaluated via decision curve analysis. In addition, we constructed life tables (starting at age 45 with 5-year intervals) to estimate remaining life expectancy at the tertiles of TyG-derived indices and compare differences between the T3 and T1 groups. The same method was applied within glycemic status strata, and cause-specific life table decomposition was used to further break down life expectancy differences into cardiovascular and non-cardiovascular components. Population attributable fractions estimated the mortality burden associated with elevated indices. Exploratory bootstrap mediation analyses assessed statistical mediation by the systemic immune-inflammation index (SII), systemic inflammatory response index (SIRI), and aggregate index of systemic inflammation (AISI) in the TyG-ABSI–mortality association; network diagrams visualised conditional association structures. Effect modification by age and sex was tested via multiplicative interaction terms.

Sensitivity analyses included assessment of multicollinearity (variance inflation factors), generalized propensity score (GPS) weighting and G-formula standardisation, inverse probability of treatment weighting (IPTW; reported as Model 3, with Model 2 representing the conventional survey-weighted Cox model), E-values for unmeasured confounding, a 2-year lag analysis excluding early deaths, false discovery rate correction for multiple comparisons, a life table sensitivity analysis using broader 10-year age bands to mitigate sparse-cell instability, and external validation in CHARLS.

Analyses used R (version 4.3.0); packages are listed in the supplemental information.

## Supplemental information

Document S1. Figures S1–S9, Tables S1, S2, S4, S7, S8, and S11, and Methods S1

Table S3. Incremental predictive value of TyG-ABSI compared with alternative TyG-derived indices

Table S5. E-value analysis of TyG index and derived metrics with mortality risk

Table S6. Association of TyG and its derived measures with all-cause and cardiovascular mortality after excluding deaths within the first 2 years of follow-up

Table S9. Association of TyG and its derivative indices with all-cause and cardiovascular mortality across conventional and IPTW models

Table S10. Association of TyG-related indices with all-cause mortality in the CHARLS validation cohort
