# Supplementary material for: Nine anthropometric insulin resistance indices predict mortality and life expectancy across glucose states
Source: iScience. 2026 Sep 7;29(9):117400. doi: 10.1016/j.isci.2026.117400 (PMC13577881; doi:10.1016/j.isci.2026.117400)
Supplement: Supplementary file 1 [file mmc1.pdf]

iScience, Volume *29*

Supplemental information

Nine anthropometric insulin resistance indices predict mortality and life expectancy across glucose states

Yi Tang, Yameng Xu, Zilong Yue, Wencai Wang, Song Wen, Haiyang Wang, Lingyun Liu, Juncang Wu, and Zhonghua Sun

Supplementary material

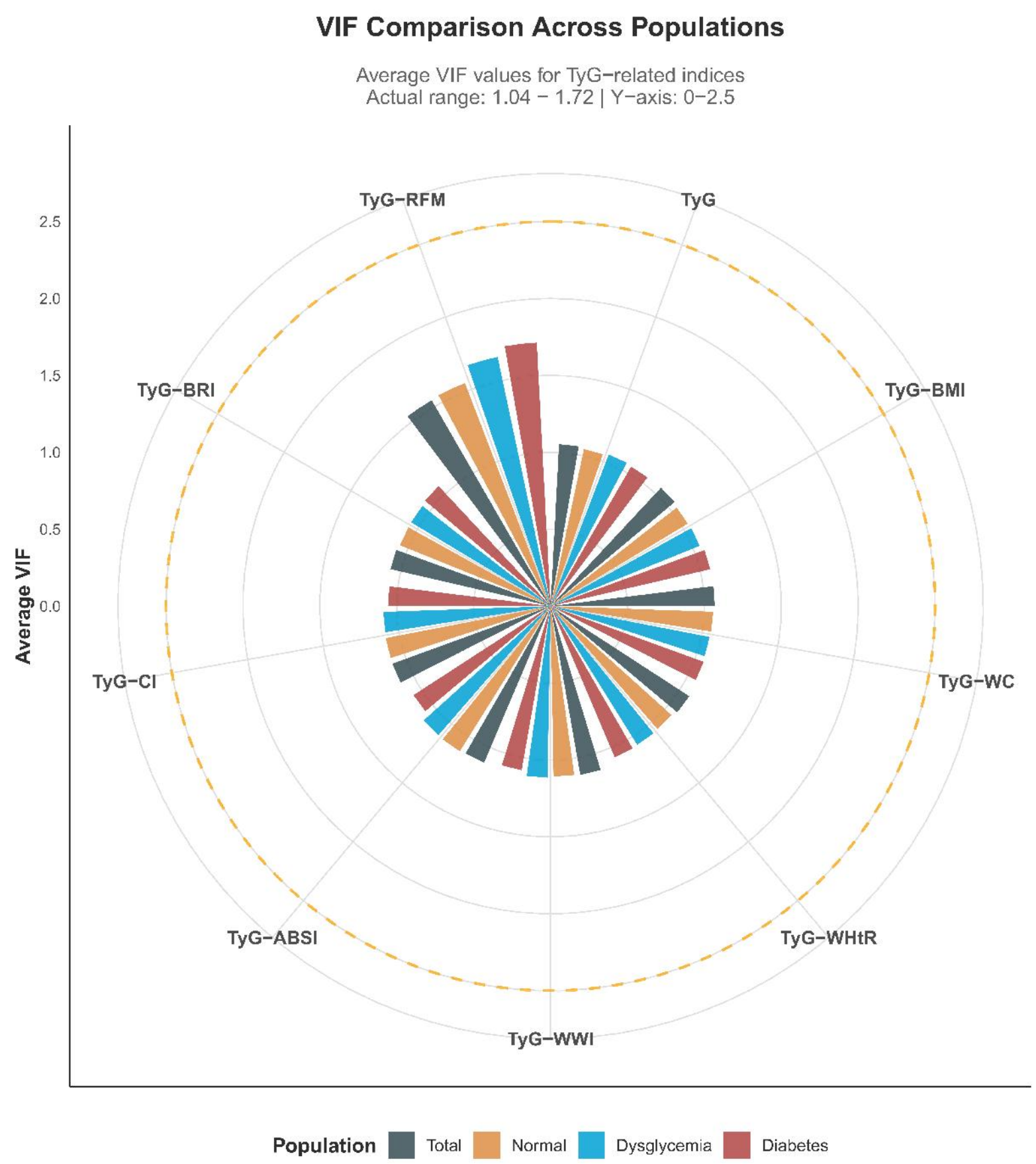

Supplementary Fig. 1. Multicollinearity assessment of TyG-related indices Using Variance Inflation Factor

Radar chart displaying the average variance inflation factor (VIF) values for nine TyG-related indices across four study populations (Total, Normal, Dysglycemia, and Diabetes). The dashed orange circle indicates the VIF threshold of 2.5. All indices demonstrated VIF values ranging from 1.04 to 1.72, well below the threshold, indicating no substantial multicollinearity concerns in the regression models.

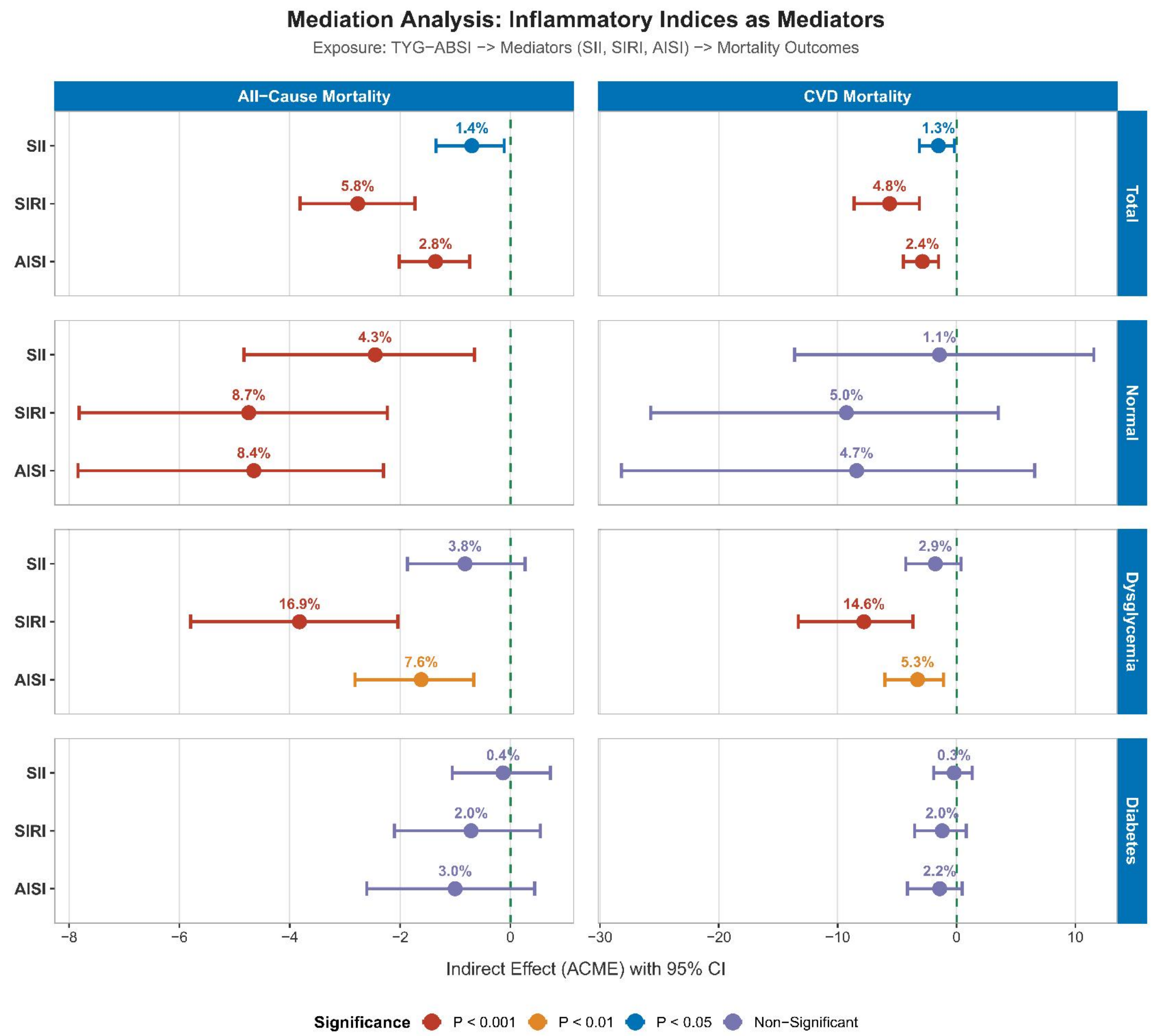

Supplementary Fig. 2. Mediation analysis of inflammatory indices in the association between TyG-ABSI and mortality. Forest plots illustrating the mediating effects of systemic inflammatory indices (SII, SIRI, and AISI) on the association between TyG-ABSI and all-cause mortality (left panels) and cardiovascular mortality (right panels). The analyses were conducted in the total population and subgroups stratified by glycemic status (normal glucose tolerance, dysglycemia, and diabetes). The dots and error bars represent the average causal mediation effects (ACME) and their 95% confidence intervals. The percentage values adjacent to the error bars indicate the proportion of the total effect mediated by each inflammatory index. Models were adjusted for age, gender, race, education, PIR, marital status, smoking, alcohol use, antihypertensive drug, hypertension, heart failure, heart disease, angina, heart attack, stroke, failing kidney, and cancer. Abbreviations: SII, systemic immune-inflammation index; SIRI, systemic inflammatory response index; AISI, aggregate index of systemic inflammation; PIR, poverty income ratio; ACME, average causal mediation effect.

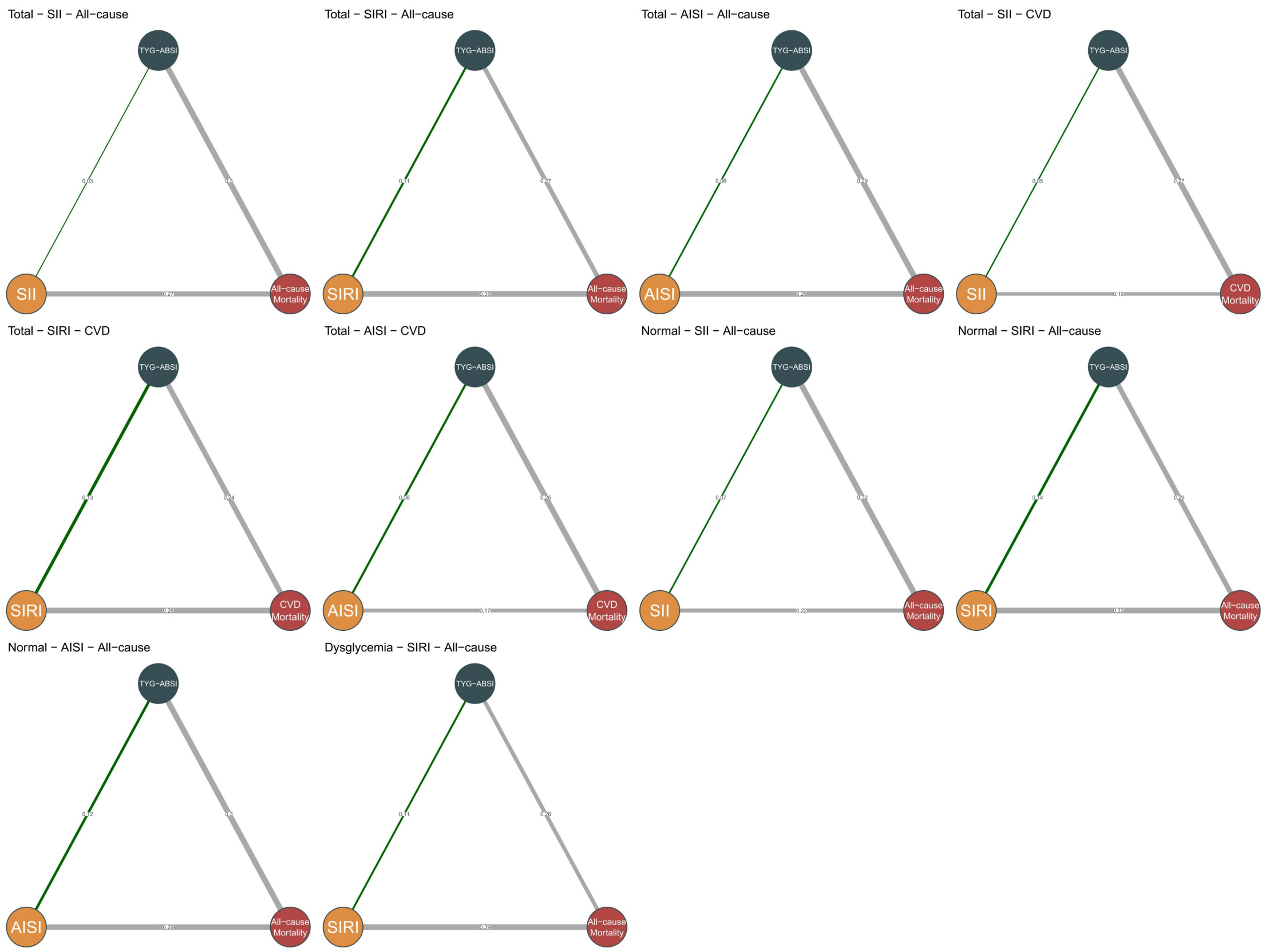

Supplementary Fig. 3. Network visualizations of the associations among TyG-ABSI, systemic inflammation indices, and mortality risks.

The network plots illustrate the pairwise conditional dependencies between the exposure (TyG-ABSI), mediators (SIRI, SII, or AISI), and outcomes (All-cause or CVD mortality) across different population subgroups. Each node represents a variable, and the edges (lines) represent the partial correlation weights between them. Thicker edges indicate stronger associations, while green edges denote positive correlations. The specific inflammation index and outcome used in each model are labeled accordingly.

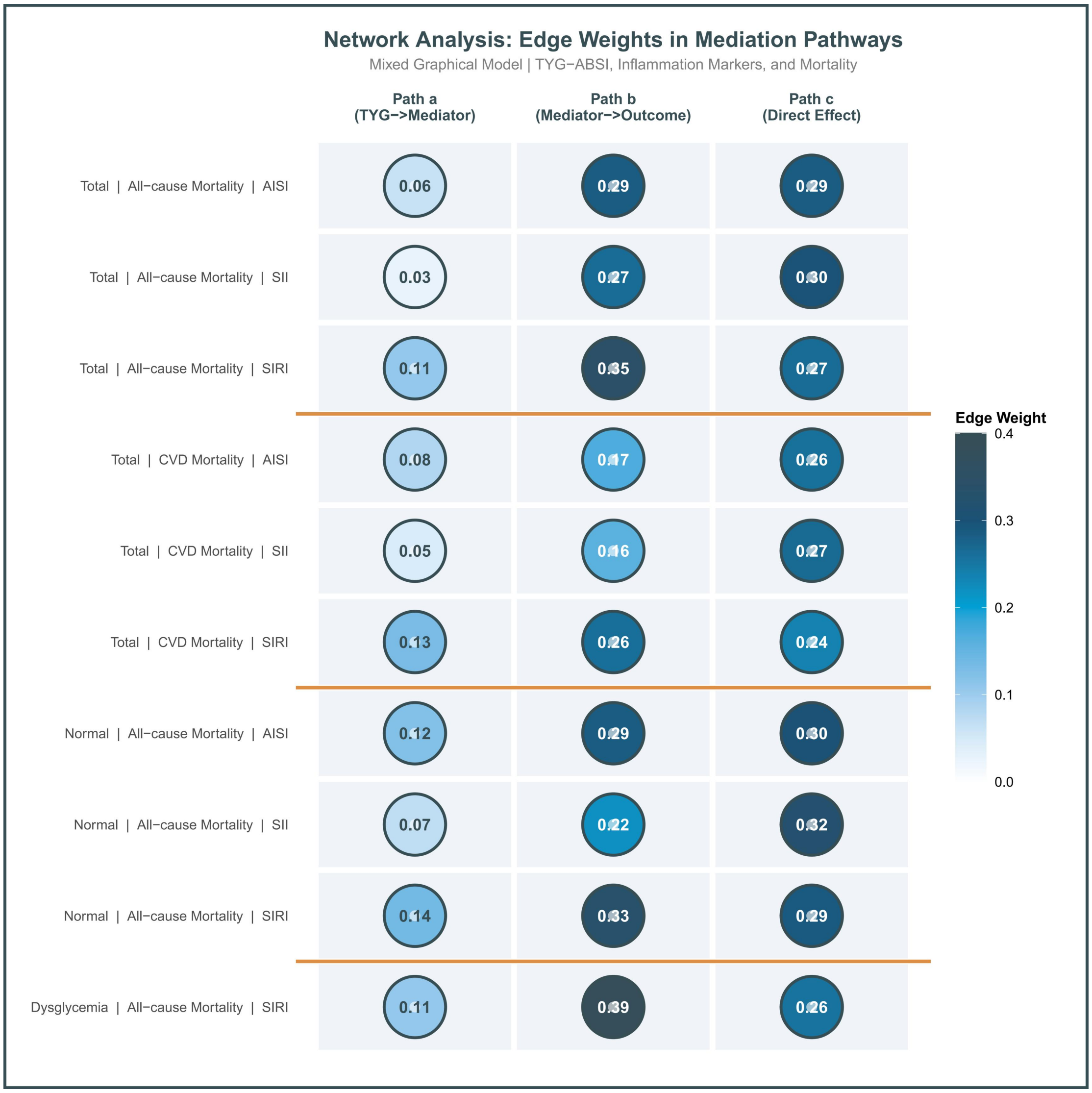

Supplementary Fig. 4. Heatmap summarizing edge weights among TyG-ABSI, inflammatory indices, and mortality outcomes.

The figure displays the numerical edge weights for the three key pathways in the network models: the association between the mediator and outcome (Mediator-Outcome), the exposure and mediator (TyG-ABSI-Mediator), and the direct association between the exposure and outcome (TyG-ABSI-Outcome). Higher values indicate a stronger connection strength within the network. Data are shown for total, normal glucose tolerance, and dysglycemia subgroups.

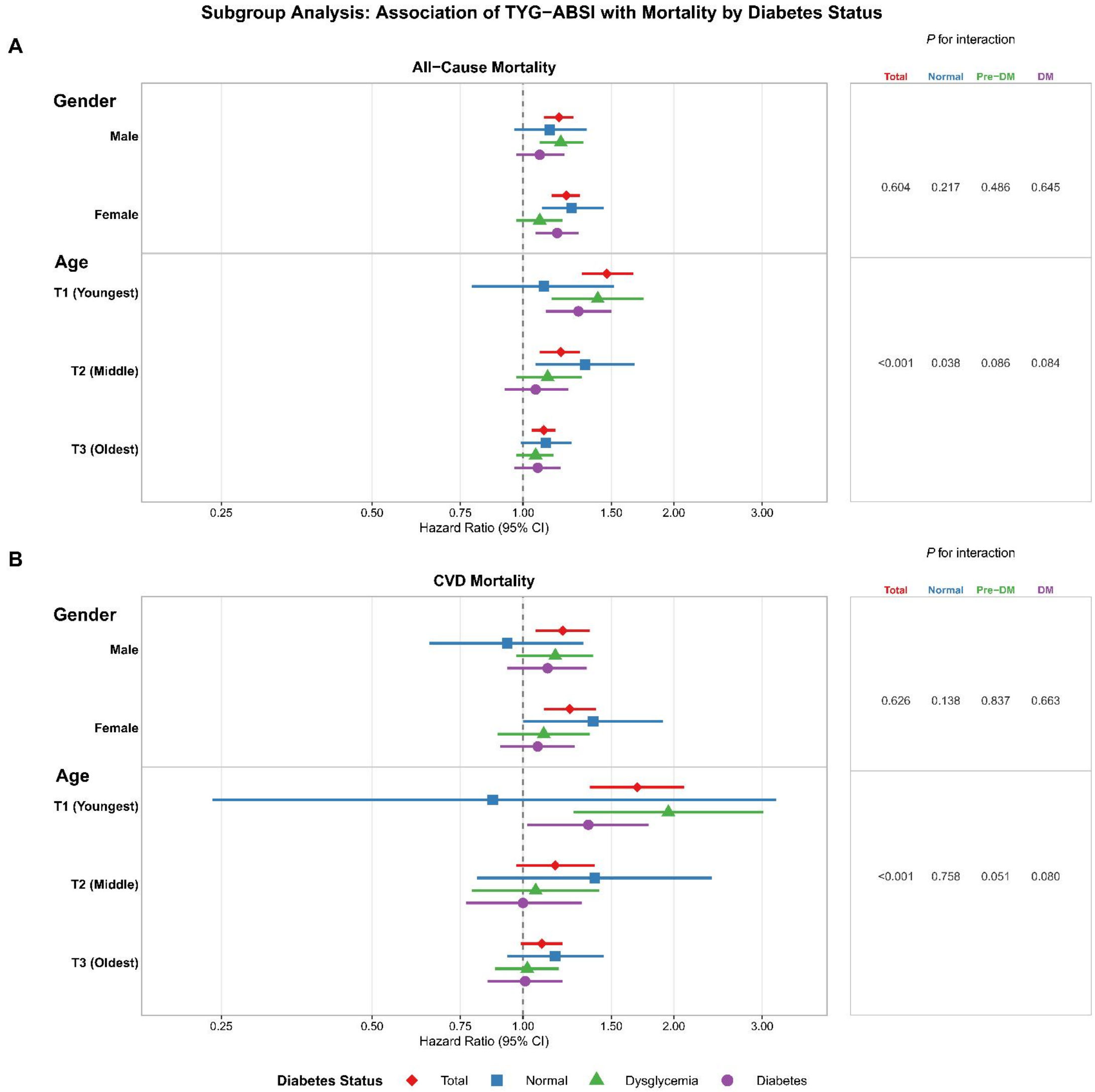

Supplementary Fig. 5. Subgroup analyses of the association between TyG-ABSI and mortality.

Solid points represent Hazard Ratios (HR) and horizontal lines indicate 95% confidence intervals (CI). ***P*** values for interaction are provided for each subgroup variable.

Models were adjusted for age, gender, race, education, PIR, marital status, smoking, alcohol use, antihypertensive drug, hypertension, heart failure, heart disease, angina, heart attack, stroke, failing kidney, and cancer. (Note: The stratification variable was excluded from the adjustment model in each respective subgroup analysis).

Total: T1: 45-56; T2: 56-68; T3: 68-85; Normal: T1: 45-53; T2: 53-64; T3: 64-85; Dysglycemia: T1: 45-56; T2: 56-68; T3: 68-85; Diabetes: T1: 45-60; T2: 60-70; T3: 70-85.

Abbreviations: PIR, poverty income ratio; HR, hazard ratio; CI, confidence interval.

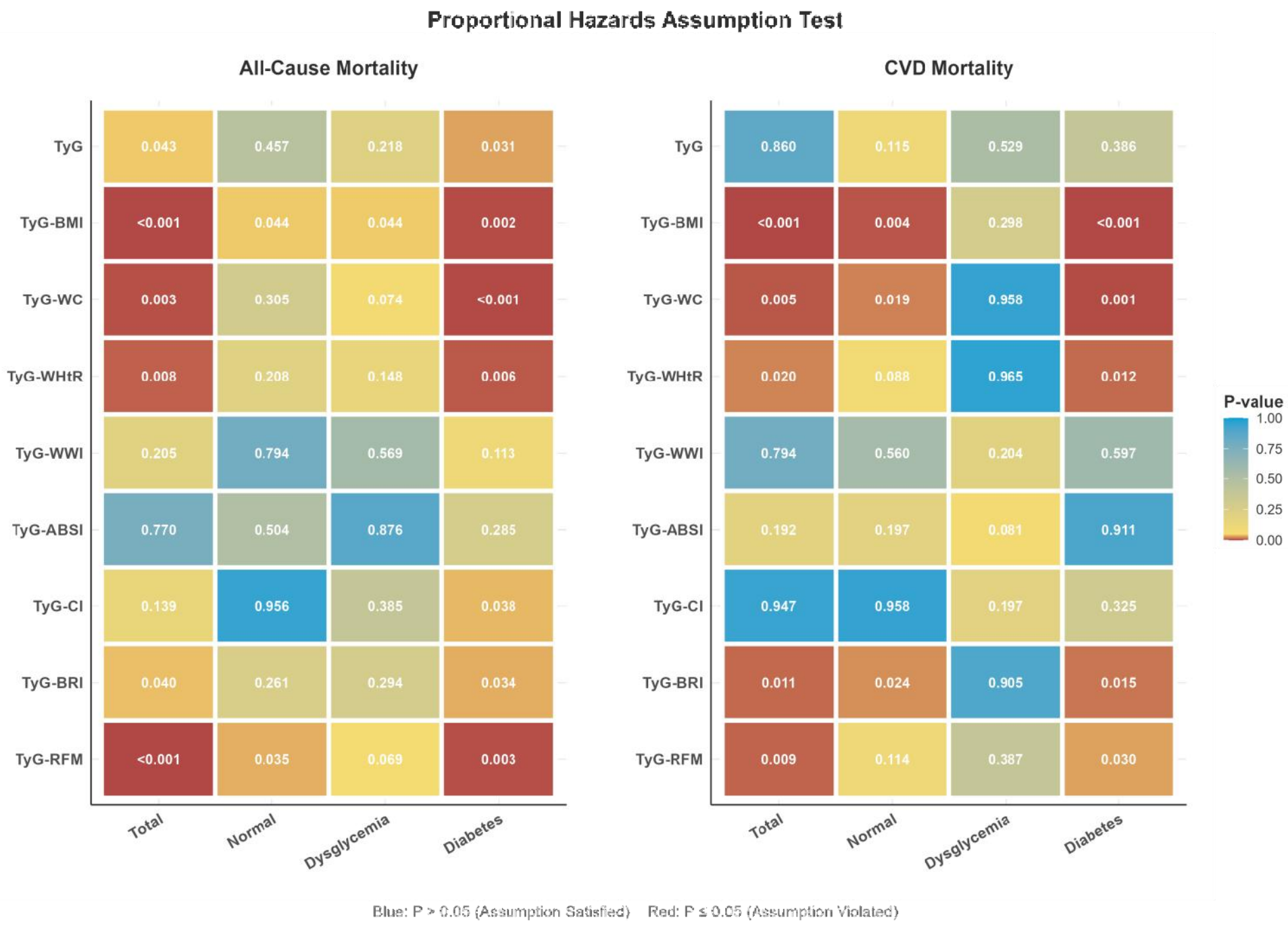

Supplementary Fig. 6. Assessment of the Proportional Hazards Assumption for TyG-Related Indices Across Study Populations.

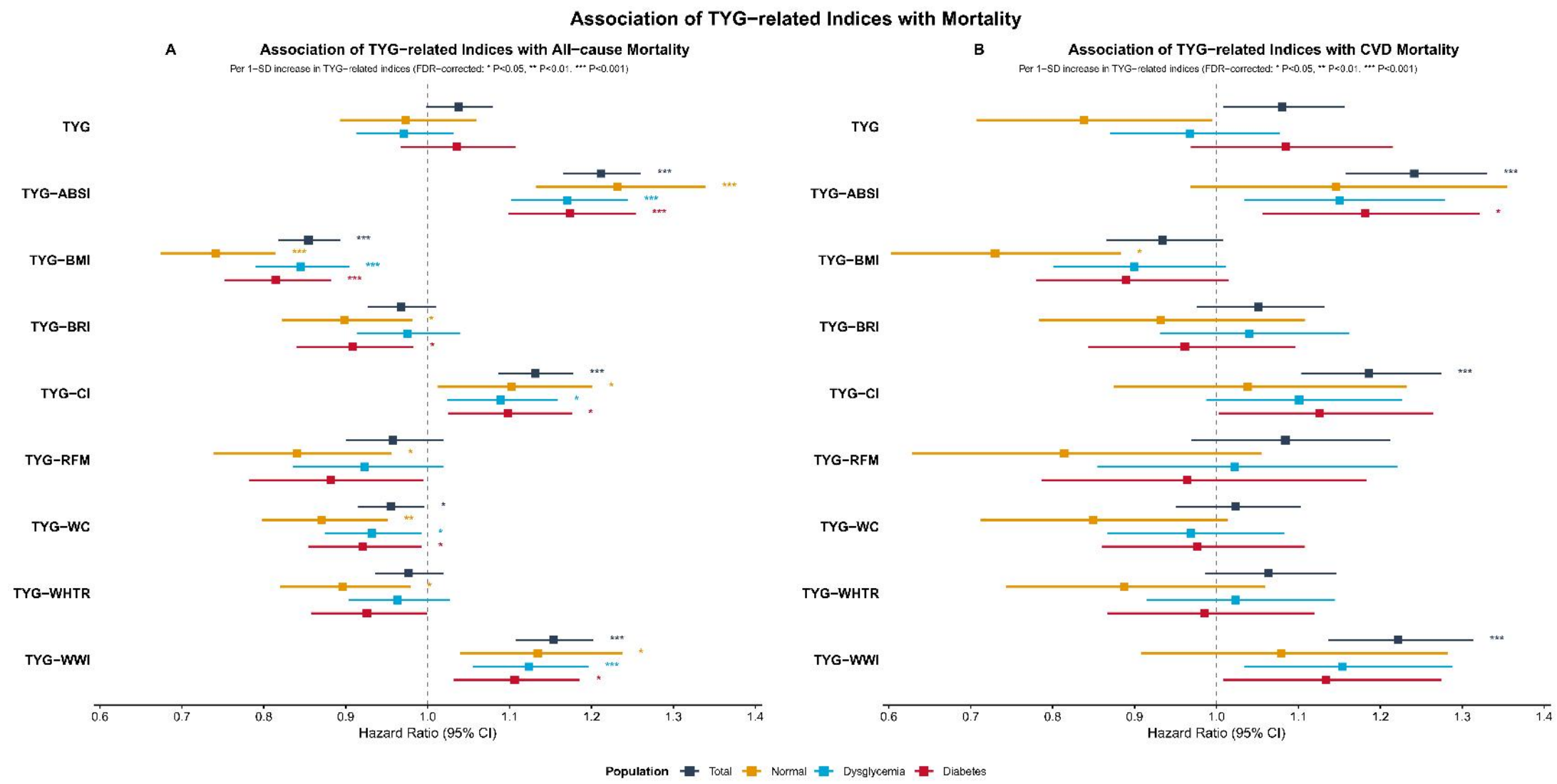

Supplementary Fig. 7. Association of TyG-related indices with all-cause and cardiovascular mortality after False Discovery Rate (FDR) correction.

Forest plots displaying the multivariable-adjusted hazard ratios (HRs) and 95% confidence intervals for all-cause mortality (A) and cardiovascular mortality (B) per 1-standard deviation (SD) increase in TyG index and TyG-related indices. For total-population estimates, SDs were calculated from the overall analytic sample; for glycaemic-status–stratified estimates, SDs were calculated separately within each corresponding glycaemic stratum. The analyses were performed in the total population and stratified by glycemic status (participants with normal glucose tolerance, dysglycemia, and diabetes). ***P*-values were adjusted for multiple comparisons using the Benjamini-Hochberg False Discovery Rate (FDR) method (*$P < 0.05$, **$P < 0.01$, ***$P < 0.001$).

Total population, n = 11,787; normoglycemia, n = 3,037; dysglycemia, n = 5,683; diabetes, n = 3,067.

Models were adjusted for age, gender, race, education, PIR, marital status, smoking, alcohol use, antihypertensive drug, hypertension, heart failure, heart disease, angina, heart attack, stroke, failing kidney, and cancer.

Abbreviations: FDR, False Discovery Rate; SD, standard deviation; PIR, poverty income ratio.

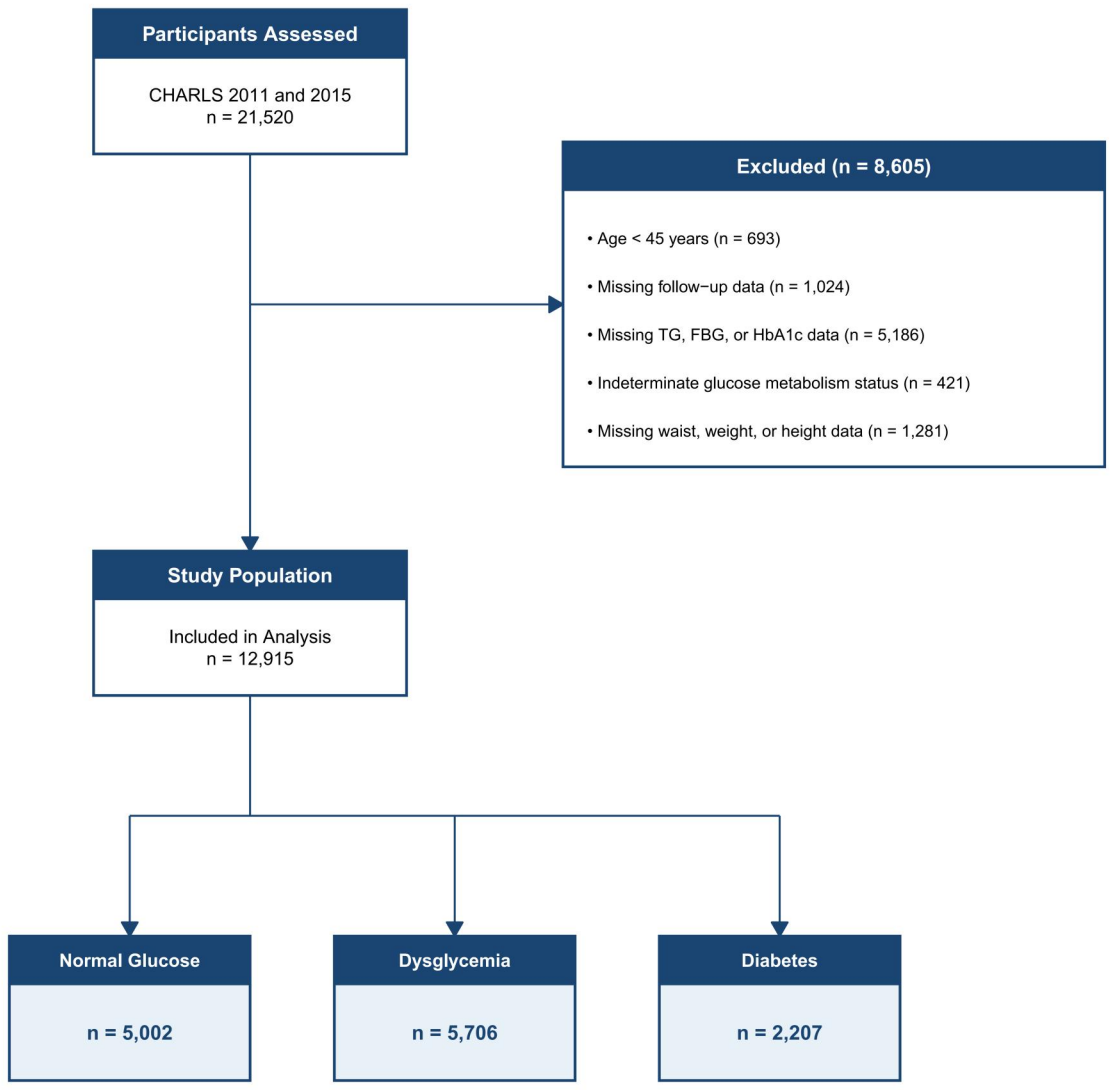

Supplementary Fig. 8. Flowchart of Participant Selection from the CHARLS 2011 and 2015 Waves
Abbreviations: CHARLS, China Health and Retirement Longitudinal Study.

Time−dependent AUC Curves of Baseline TyG−related Indices (All−cause Mortality, CHARLS)

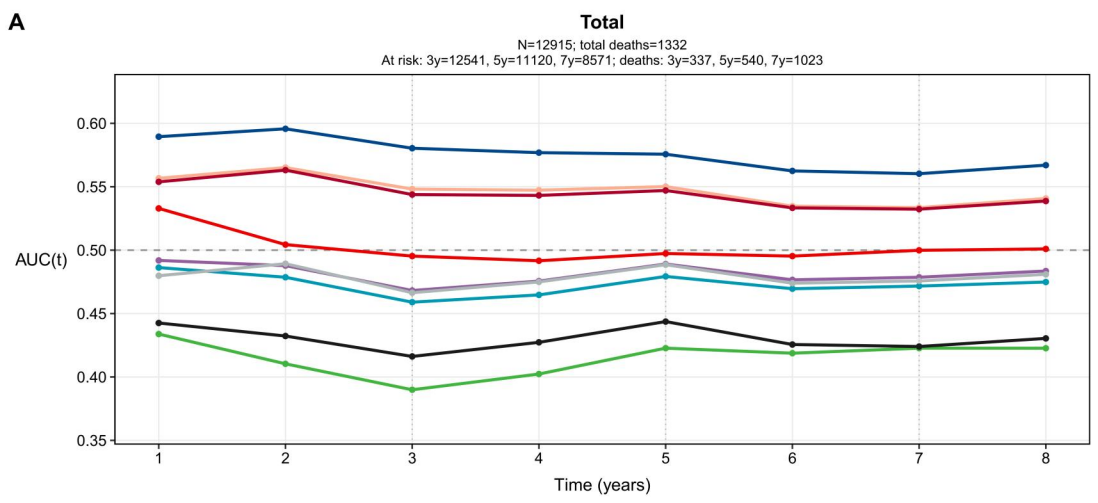

Index	AUC 3y	dAUC 3y	AUC 5y	dAUC 5y	AUC 7y	dAUC 7y
TyG−ABSI	0.580 (0.544,0.618)	Ref.	0.576 (0.553,0.601)	Ref.	0.560 (0.540,0.578)	Ref.
TyG	0.495 (0.457,0.532)	+0.085 (+0.058,+0.111)	0.497 (0.472,0.526)	+0.078 (+0.059,+0.094)	0.500 (0.481,0.520)	+0.060 (+0.045,+0.072)
TyG−BMI	0.390 (0.351,0.430)	+0.190 (+0.150,+0.227)	0.423 (0.395,0.454)	+0.153 (+0.128,+0.180)	0.423 (0.403,0.446)	+0.138 (+0.116,+0.156)
TyG−WC	0.459 (0.417,0.498)	+0.121 (+0.090,+0.151)	0.479 (0.451,0.510)	+0.096 (+0.076,+0.119)	0.472 (0.453,0.495)	+0.089 (+0.074,+0.103)
TyG−WHtR	0.468 (0.426,0.505)	+0.112 (+0.084,+0.138)	0.489 (0.461,0.520)	+0.087 (+0.068,+0.107)	0.479 (0.460,0.502)	+0.082 (+0.066,+0.096)
TyG−WWI	0.548 (0.512,0.589)	+0.032 (+0.021,+0.044)	0.550 (0.525,0.577)	+0.026 (+0.017,+0.033)	0.534 (0.513,0.556)	+0.027 (+0.019,+0.034)
TyG−CI	0.544 (0.505,0.584)	+0.037 (+0.026,+0.046)	0.547 (0.524,0.574)	+0.029 (+0.022,+0.036)	0.532 (0.512,0.553)	+0.028 (+0.023,+0.033)
TyG−BRI	0.466 (0.422,0.505)	+0.114 (+0.080,+0.143)	0.489 (0.459,0.518)	+0.087 (+0.066,+0.111)	0.476 (0.457,0.498)	+0.085 (+0.067,+0.101)
TyG−RFM	0.416 (0.376,0.453)	+0.164 (+0.128,+0.196)	0.444 (0.418,0.469)	+0.132 (+0.110,+0.155)	0.424 (0.405,0.447)	+0.136 (+0.115,+0.153)

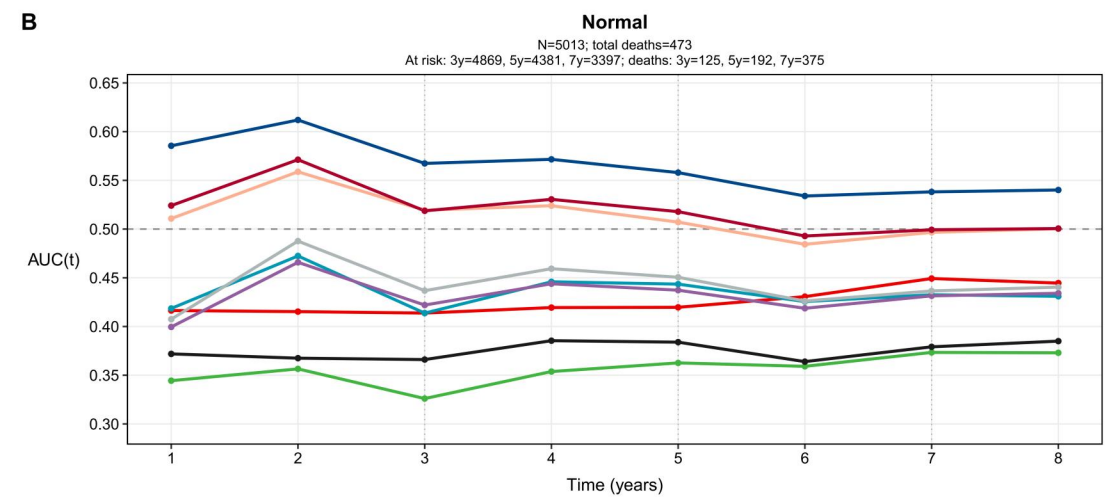

Index	AUC 3y	dAUC 3y	AUC 5y	dAUC 5y	AUC 7y	dAUC 7y
TyG−ABSI	0.567 (0.513,0.621)	Ref.	0.558 (0.513,0.605)	Ref.	0.538 (0.509,0.572)	Ref.
TyG	0.414 (0.364,0.467)	+0.154 (+0.105,+0.211)	0.420 (0.382,0.458)	+0.138 (+0.106,+0.175)	0.449 (0.417,0.480)	+0.089 (+0.066,+0.117)
TyG−BMI	0.326 (0.276,0.383)	+0.241 (+0.174,+0.314)	0.363 (0.329,0.404)	+0.195 (+0.146,+0.247)	0.373 (0.343,0.407)	+0.165 (+0.129,+0.205)
TyG−WC	0.414 (0.360,0.467)	+0.154 (+0.102,+0.203)	0.443 (0.411,0.488)	+0.114 (+0.083,+0.147)	0.433 (0.401,0.468)	+0.106 (+0.080,+0.135)
TyG−WHtR	0.422 (0.370,0.475)	+0.145 (+0.098,+0.192)	0.437 (0.401,0.485)	+0.121 (+0.087,+0.155)	0.431 (0.400,0.468)	+0.107 (+0.084,+0.133)
TyG−WWI	0.519 (0.463,0.576)	+0.048 (+0.024,+0.070)	0.507 (0.467,0.558)	+0.051 (+0.034,+0.068)	0.497 (0.466,0.532)	+0.042 (+0.030,+0.056)
TyG−CI	0.519 (0.465,0.574)	+0.049 (+0.032,+0.065)	0.518 (0.477,0.567)	+0.040 (+0.029,+0.051)	0.499 (0.469,0.537)	+0.039 (+0.031,+0.048)
TyG−BRI	0.437 (0.386,0.490)	+0.131 (+0.074,+0.190)	0.451 (0.412,0.500)	+0.107 (+0.068,+0.150)	0.437 (0.406,0.474)	+0.102 (+0.077,+0.132)
TyG−RFM	0.366 (0.309,0.430)	+0.201 (+0.146,+0.261)	0.384 (0.340,0.424)	+0.174 (+0.132,+0.219)	0.379 (0.347,0.409)	+0.159 (+0.133,+0.192)

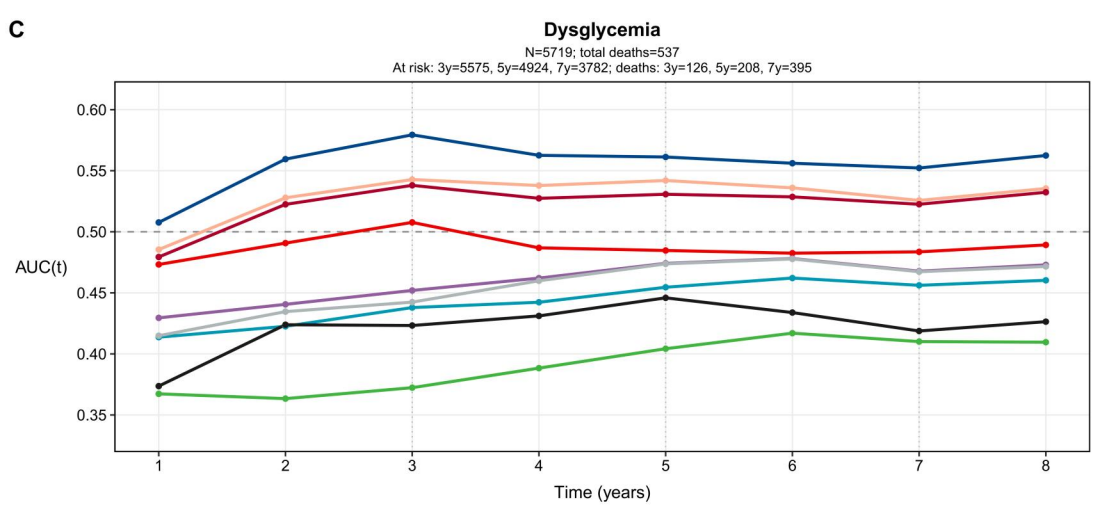

Index	AUC 3y	dAUC 3y	AUC 5y	dAUC 5y	AUC 7y	dAUC 7y
TyG−ABSI	0.579 (0.515,0.637)	Ref.	0.561 (0.521,0.603)	Ref.	0.552 (0.523,0.580)	Ref.
TyG	0.508 (0.442,0.567)	+0.072 (+0.027,+0.119)	0.485 (0.448,0.529)	+0.077 (+0.046,+0.111)	0.484 (0.457,0.513)	+0.069 (+0.042,+0.095)
TyG−BMI	0.372 (0.317,0.433)	+0.207 (+0.146,+0.281)	0.404 (0.367,0.447)	+0.157 (+0.118,+0.203)	0.410 (0.380,0.444)	+0.142 (+0.107,+0.173)
TyG−WC	0.438 (0.378,0.499)	+0.141 (+0.090,+0.201)	0.455 (0.417,0.496)	+0.107 (+0.078,+0.139)	0.456 (0.429,0.487)	+0.096 (+0.071,+0.118)
TyG−WHtR	0.452 (0.391,0.518)	+0.127 (+0.083,+0.182)	0.474 (0.432,0.521)	+0.087 (+0.057,+0.119)	0.468 (0.439,0.496)	+0.084 (+0.060,+0.106)
TyG−WWI	0.543 (0.482,0.598)	+0.037 (+0.019,+0.057)	0.542 (0.498,0.587)	+0.019 (+0.005,+0.034)	0.526 (0.495,0.555)	+0.027 (+0.016,+0.036)
TyG−CI	0.538 (0.474,0.599)	+0.041 (+0.025,+0.059)	0.531 (0.489,0.575)	+0.031 (+0.020,+0.042)	0.523 (0.493,0.552)	+0.030 (+0.020,+0.038)
TyG−BRI	0.442 (0.383,0.506)	+0.137 (+0.086,+0.197)	0.474 (0.429,0.518)	+0.088 (+0.053,+0.123)	0.467 (0.437,0.495)	+0.085 (+0.058,+0.107)
TyG−RFM	0.423 (0.363,0.482)	+0.156 (+0.107,+0.204)	0.446 (0.405,0.487)	+0.115 (+0.082,+0.151)	0.419 (0.387,0.449)	+0.133 (+0.107,+0.159)

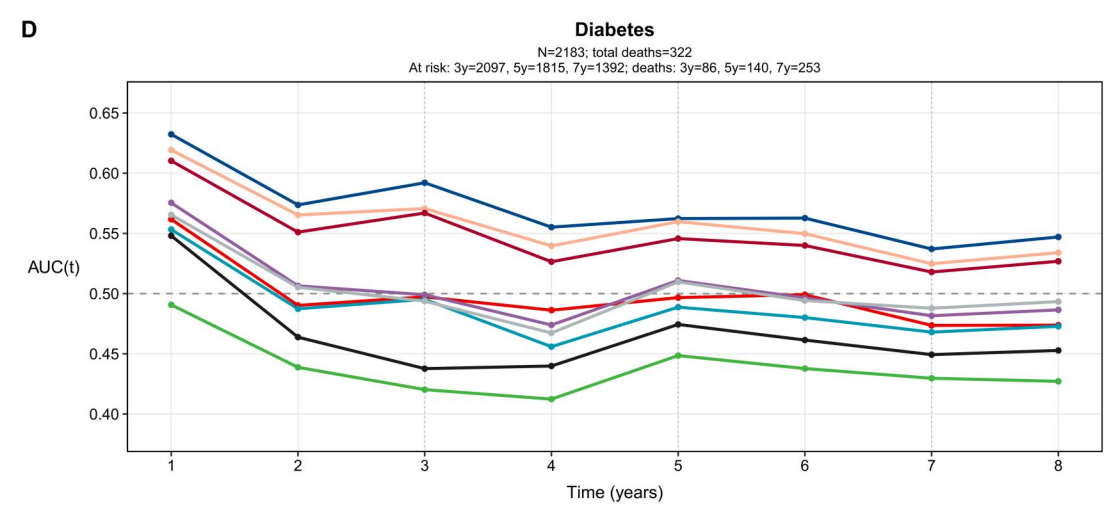

Index	AUC 3y	dAUC 3y	AUC 5y	dAUC 5y	AUC 7y	dAUC 7y
TyG−ABSI	0.592 (0.517,0.668)	Ref.	0.562 (0.512,0.609)	Ref.	0.537 (0.495,0.571)	Ref.
TyG	0.497 (0.429,0.576)	+0.095 (+0.050,+0.139)	0.497 (0.449,0.550)	+0.066 (+0.037,+0.095)	0.474 (0.433,0.517)	+0.063 (+0.038,+0.085)
TyG−BMI	0.420 (0.360,0.492)	+0.172 (+0.100,+0.244)	0.449 (0.402,0.497)	+0.114 (+0.058,+0.164)	0.430 (0.393,0.472)	+0.107 (+0.065,+0.146)
TyG−WC	0.495 (0.426,0.579)	+0.097 (+0.041,+0.148)	0.489 (0.445,0.537)	+0.074 (+0.033,+0.111)	0.468 (0.426,0.510)	+0.069 (+0.039,+0.096)
TyG−WHtR	0.499 (0.430,0.578)	+0.093 (+0.042,+0.141)	0.511 (0.467,0.556)	+0.051 (+0.013,+0.084)	0.482 (0.440,0.523)	+0.055 (+0.026,+0.081)
TyG−WWI	0.571 (0.494,0.649)	+0.021 (−0.001,+0.044)	0.560 (0.511,0.609)	+0.003 (−0.013,+0.018)	0.525 (0.484,0.563)	+0.012 (−0.001,+0.027)
TyG−CI	0.567 (0.495,0.649)	+0.025 (+0.006,+0.042)	0.546 (0.500,0.593)	+0.017 (+0.004,+0.029)	0.518 (0.476,0.554)	+0.019 (+0.008,+0.028)
TyG−BRI	0.494 (0.427,0.570)	+0.098 (+0.035,+0.161)	0.510 (0.462,0.558)	+0.053 (+0.008,+0.090)	0.488 (0.447,0.529)	+0.049 (+0.014,+0.084)
TyG−RFM	0.438 (0.372,0.510)	+0.154 (+0.082,+0.223)	0.474 (0.424,0.520)	+0.088 (+0.039,+0.140)	0.449 (0.410,0.488)	+0.088 (+0.050,+0.133)

Index: TyG−ABSI, TyG, TyG−BMI, TyG−WC, TyG−WHtR, TyG−WWI, TyG−CI, TyG−BRI, TyG−RFM

Note: AUC(t) was estimated using baseline TyG−related indices and does not represent a dynamically updated time−varying prediction model. Values are AUC or dAUC with 95% bootstrap CIs. dAUC = AUC(TyG−ABSI) − AUC(comparator).

Supplementary Fig. 9. Time-dependent discriminative performance of TyG-related indices for predicting all-cause mortality in the CHARLS cohort.

Time-dependent AUC(t) curves were estimated using baseline TyG-related indices as fixed predictors and therefore did not represent dynamically updated time-varying prediction models. Panels show the total CHARLS cohort and subgroups stratified by glycemic status. Embedded summaries show AUC estimates with 95% bootstrap CIs and ΔAUC values [AUC(TyG-ABSI) − AUC(comparator)] at 3, 5, and 7 years, together with sample size, cumulative deaths, and number at risk.

Abbreviations: AUC, area under the receiver operating characteristic curve; TyG, triglyceride-glucose index; BMI, body mass index; WC, waist circumference; WHtR, waist-to-height ratio; WWI, weight-adjusted waist index; ABSI, a body shape index; CI, conicity index; BRI, body roundness index; RFM, relative fat mass; CHARLS, China Health and Retirement Longitudinal Study.

Table S1. Comparison of baseline characteristics between the final analytical sample and participants excluded due to missing data, related to STAR Methods.

Characteristic	Included (n = 11,787)	Excluded (n = 19,789)	P-value
N	11,787	19,789	
Age (years)	62.55 ± 11.02 (n = 11,787)	63.83 ± 11.66 (n = 19,789)	<0.001
PIR	2.63 ± 1.61 (n = 11,787)	2.60 ± 1.62 (n = 17,564)	0.075
Waist circumference (cm)	101.20 ± 14.98 (n = 11,787)	101.17 ± 15.05 (n = 16,064)	0.859
Height (cm)	166.36 ± 10.11 (n = 11,787)	165.88 ± 10.20 (n = 17,464)	<0.001
Weight (kg)	80.88 ± 19.75 (n = 11,787)	80.70 ± 20.41 (n = 17,499)	0.454
Follow-up (months)	101.52 ± 60.59 (n = 11,787)	106.68 ± 66.58 (n = 19,723)	<0.001
HbA1c (%)	5.98 ± 1.17 (n = 11,787)	5.98 ± 1.19 (n = 16,749)	0.586
Fasting glucose (mg/dL)	115.32 ± 40.08 (n = 11,787)	121.07 ± 49.35 (n = 878)	<0.001
Triglycerides (mg/dL)	138.14 ± 110.66 (n = 11,787)	175.04 ± 134.40 (n = 16,202)	<0.001
TyG index	8.78 ± 0.65 (n = 11,787)	8.85 ± 0.68 (n = 672)	0.007
TyG-BMI	256.71 ± 62.66 (n = 11,787)	271.52 ± 85.92 (n = 465)	<0.001
TyG-WC	891.13 ± 163.66 (n = 11,787)	925.69 ± 181.46 (n = 53)	0.172
TyG-WHtR	5.37 ± 0.98 (n = 11,787)	5.39 ± 0.95 (n = 32)	0.903
TyG-WWI	99.48 ± 11.30 (n = 11,787)	101.95 ± 11.45 (n = 37)	0.198
TyG-ABSI	0.73 ± 0.07 (n = 11,787)	0.73 ± 0.07 (n = 18)	0.726
TyG-CI	11.76 ± 1.30 (n = 11,787)	11.53 ± 1.01 (n = 18)	0.359
TyG-BRI	51.30 ± 20.49 (n = 11,787)	50.73 ± 20.65 (n = 32)	0.877
TyG-RFM	321.56 ± 78.66 (n = 11,787)	313.33 ± 82.87 (n = 32)	0.578
Sex	Available n = 11,787	Available n = 19,789	0.220
Male	5826 (49.4%)	9640 (48.7%)	
Female	5961 (50.6%)	10149 (51.3%)	
Race/ethnicity	Available n = 11,787	Available n = 19,789	<0.001
Mexican American	1843 (15.6%)	2929 (14.8%)	
Other Hispanic	1064 (9.0%)	1478 (7.5%)	
Non-Hispanic White	5563 (47.2%)	9559 (48.3%)	
Non-Hispanic Black	2301 (19.5%)	4259 (21.5%)	
Other Race	1016 (8.6%)	1564 (7.9%)	
Education	Available n = 11,787	Available n = 19,710	<0.001
Less than 9th grade	1803 (15.3%)	3314 (16.8%)	
9-11th grade	1736 (14.7%)	2970 (15.1%)	
High school graduate/GED	2722 (23.1%)	4607 (23.4%)	

**Characteristic**	**Included (n = 11,787)**	**Excluded (n = 19,789)**	**P-value**
Some college or AA degree	3047 (25.9%)	4934 (25.0%)	
College graduate or above	2479 (21.0%)	3885 (19.7%)	
Marital status	Available n = 11,787	Available n = 19,559	<0.001
Married/Living with partner	7397 (62.8%)	11453 (58.6%)	
Others	4390 (37.2%)	8106 (41.4%)	
Smoking	Available n = 11,787	Available n = 19,750	0.114
Yes	6008 (51.0%)	9885 (50.1%)	
No	5779 (49.0%)	9865 (49.9%)	

Abbreviations: PIR, household income-poverty ratio; BMI, body mass index; GED, General Educational Development Certificate; AA, Associate Degree.

Table S2. Baseline characteristics of participants stratified by all-cause mortality in the CHARLS database, related to Table 1.

	All-cause mortality			
Characteristics	Total	Alive	Deceased	***P*-value**
N	12915	11583	1332	
Age (year)	57.609 ± 10.164	56.443 ± 9.464	67.749 ± 10.405	<0.001
Total family consumption (CNY)	24037.957 ± 43733.227	24816.024 ± 45085.806	17271.933 ± 28593.183	<0.001
Height (m)	1.582 ± 0.091	1.583 ± 0.090	1.573 ± 0.098	<0.001
Waist (cm)	85.607 ± 10.176	85.763 ± 10.103	84.246 ± 10.697	<0.001
Weight (kg)	59.567 ± 11.644	60.019 ± 11.545	55.638 ± 11.764	<0.001
HbA1c (%)	5.424 ± 0.880	5.422 ± 0.849	5.443 ± 1.116	0.410
Fasting Blood Glucose (mg/dL)	107.961 ± 36.307	106.881 ± 33.530	117.357 ± 53.927	<0.001
Triglycerides (mg/dL)	136.454 ± 106.608	137.336 ± 107.283	128.783 ± 100.256	0.006
TyG	8.691 ± 0.671	8.691 ± 0.667	8.695 ± 0.708	0.805
TyG-BMI	209.173 ± 105.691	210.375 ± 102.328	198.724 ± 130.936	<0.001
TyG-WC	746.301 ± 121.899	747.640 ± 121.467	734.659 ± 125.036	<0.001
TyG-WHtR	4.733 ± 0.863	4.737 ± 0.849	4.696 ± 0.979	0.103
TyG-WWI	97.049 ± 11.916	96.808 ± 11.578	99.147 ± 14.366	<0.001
TyG-ABSI	0.720 ± 0.085	0.718 ± 0.080	0.741 ± 0.119	<0.001
TyG-CI	11.184 ± 1.328	11.161 ± 1.297	11.382 ± 1.561	<0.001
TyG-BRI	37.930 ± 28.741	37.948 ± 26.282	37.773 ± 44.769	0.833
TyG-RFM	288.630 ± 81.376	290.823 ± 80.280	269.560 ± 88.110	<0.001
**Follow-up time (month)**	89.588 ± 25.104	92.907 ± 22.505	60.725 ± 27.888	<0.001
Gender				<0.001
Female	7019 (54.348%)	6496 (56.082%)	523 (39.264%)	
Male	5896 (45.652%)	5087 (43.918%)	809 (60.736%)	
**Nation**				<0.001
Han Chinese	11457 (88.711%)	10690 (92.290%)	767 (57.583%)	
Others	1458 (11.289%)	893 (7.710%)	565 (42.417%)	
**Education**				<0.001
Illiterate	6064 (46.953%)	5241 (45.247%)	823 (61.787%)	
Elementary school/private tutoring	3001 (23.237%)	2706 (23.362%)	295 (22.147%)	
Junior high school	2572 (19.915%)	2417 (20.867%)	155 (11.637%)	

High school/vocational high school/technical secondary school	1278 (9.895%)	1219 (10.524%)	59 (4.429%)	
**Marital status**				<0.001
Married and living with spouse	10854 (84.042%)	9926 (85.695%)	928 (69.670%)	
Others	2061 (15.958%)	1657 (14.305%)	404 (30.330%)	
**Residence**				<0.001
Urban area	4874 (37.739%)	4440 (38.332%)	434 (32.583%)	
Rural areas	8041 (62.261%)	7143 (61.668%)	898 (67.417%)	
**Smoking**				<0.001
No	9128 (70.678%)	8318 (71.812%)	810 (60.811%)	
Yes	3787 (29.322%)	3265 (28.188%)	522 (39.189%)	
**Alcohol use**				0.187
No	8567 (66.334%)	7705 (66.520%)	862 (64.715%)	
Yes	4348 (33.666%)	3878 (33.480%)	470 (35.285%)	
**Hypertension**				<0.001
No	9443 (73.117%)	8591 (74.169%)	852 (63.964%)	
Yes	3472 (26.883%)	2992 (25.831%)	480 (36.036%)	
**Dyslipidemia**				0.390
No	11357 (87.937%)	10176 (87.853%)	1181 (88.664%)	
Yes	1558 (12.063%)	1407 (12.147%)	151 (11.336%)	
Heart disease				<0.001
No	11238 (87.021%)	10141 (87.551%)	1097 (82.357%)	
Yes	1677 (12.979%)	1442 (12.449%)	235 (17.643%)	
**Stroke**				<0.001
No	12559 (97.255%)	11304 (97.591%)	1255 (94.219%)	
Yes	356 (2.745%)	279 (2.409%)	77 (5.781%)	
**Antidiabetic Drugs**				<0.001
No	10411 (95.584%)	9223 (95.953%)	1188 (92.812%)	
Yes	481 (4.416%)	389 (4.047%)	92 (7.187%)	
**Kidney disease**				0.206
No	12672 (98.117%)	11371 (98.170%)	1301 (97.673%)	
Yes	243 (1.883%)	212 (1.830%)	31 (2.327%)	
**State of Glucose Metabolism**				<0.001
Normal glucose metabolism	5002 (38.737%)	4529 (39.100%)	473 (35.511%)	
Diabetes	2207 (17.091%)	1883 (16.257%)	324 (24.324%)	

Dysglycemia	5706 (44.172%)	5171 (44.643%)	535 (40.165%)	

Abbreviations: TyG, triglyceride-glucose index; BMI, body mass index; WC, waist circumference; WHtR, waist-to-height ratio; WWI, weight-adjusted waist index; ABSI, a body shape index; CI, conicity index; BRI, Body Roundness Index; RFM, Relative Fat Mass.

Table S4. Test for proportional hazards assumption using time-varying coefficient Cox models, related to STAR Methods.

Exposure	Total Population		Normal Glucose		Dysglycemia		Diabetes	
	All-cause	CVD	All-cause	CVD	All-cause	CVD	All-cause	CVD
TyG index	**0.018**	0.616	0.728	0.070	0.250	0.678	**0.003**	0.237
TyG-BMI	**<0.001**	**<0.001**	0.304	**0.014**	**0.049**	0.501	**<0.001**	**0.002**
TyG-WC	**0.003**	**0.005**	0.914	0.070	0.051	0.977	**<0.001**	**0.010**
TyG-WHtR	**0.007**	**0.015**	0.689	0.068	0.182	0.790	**0.002**	**0.027**
TyG-WWI	0.154	0.782	0.720	0.991	0.701	0.220	**0.034**	0.573
TyG-CI	0.085	0.527	0.388	0.998	0.348	0.315	**0.010**	0.353
TyG-BRI	**0.031**	**0.010**	0.745	**0.013**	0.315	0.818	**0.017**	**0.031**
TyG-RFM	**<0.001**	**0.017**	**0.030**	**0.028**	0.114	0.976	**0.001**	0.051
TyG-ABSI	0.618	0.476	0.166	0.280	0.918	0.153	0.138	0.958

Values are P-values for the interaction term between the exposure and the logarithm of follow-up time (Time-Varying Coefficient Cox Models).

Bold values ($P < 0.05$) indicate a statistically significant violation of the proportional hazards assumption (i.e., the hazard ratio changes over time).

Non-bold values ($P \geq 0.05$) indicate that the proportional hazards assumption is satisfied (i.e., the hazard ratio remains stable).

Abbreviations: CVD, cardiovascular disease; TyG, triglyceride-glucose index; BMI, body mass index; WC, waist circumference; WHtR, waist-to-height ratio; WWI, weight-adjusted-waist index; CI, conicity index; BRI, body roundness index; RFM, relative fat mass; ABSI, a body shape index.

Table S7. Association between TyG–ABSI and cardiovascular mortality using Fine–Gray competing-risk models, related to Table 3.

Population	TyG-ABSI (z score)	HR (95% CI)	*P*-value
Total	Continuous (per 1-SD)	1.16 (1.07 - 1.25)	< 0.001
	T2 (vs T1)	1.24 (1.02 - 1.51)	0.032
	T3 (vs T1)	1.31 (1.07 - 1.59)	0.008
	P for trend		0.003
Normal	Continuous (per 1-SD)	1.02 (0.84 - 1.23)	0.850
	T2 (vs T1)	0.67 (0.43 - 1.04)	0.073
	T3 (vs T1)	0.78 (0.52 - 1.17)	0.230
	P for trend		0.230
Dysglycemia	Continuous (per 1-SD)	1.04 (0.93 - 1.15)	0.510
	T2 (vs T1)	1.61 (1.20 - 2.18)	0.002
	T3 (vs T1)	1.32 (0.98 - 1.80)	0.071
	P for trend		0.015
Diabetes	Continuous (per 1-SD)	1.15 (1.02 - 1.30)	0.022
	T2 (vs T1)	1.00 (0.74 - 1.37)	0.970
	T3 (vs T1)	1.36 (1.02 - 1.83)	0.038
	P for trend		0.038

The model was adjusted for the following variables: age, gender, race, education, PIR, marital status, smoking, alcohol use, antihypertensive drug, hypertension, heart failure, heart disease, angina, heart attack, stroke, failing kidney, and cancer.

Note: Continuous estimates are expressed per 1-SD increase in TyG-ABSI. For the total population, the SD was calculated from the overall analytic sample; for glycaemic-status–stratified analyses, SDs were calculated separately within each corresponding glycaemic stratum.

Abbreviations: TyG–ABSI, triglyceride–glucose index combined with a body shape index; HR, hazard ratio; CI, confidence interval.

Table S8. Comparison of Cox model 2, GPS weighting, and G-formula estimates for TyG-related indices and mortality, related to STAR Methods.

	Cox Model 2		GPS-Weighted		G-formula	
	HR (95%CI)	*P*-value	HR (95%CI)	*P*-value	RR (95%CI)	*P*-value
**All-cause mortality**						
**Total**						
TyG index (Per SD increase)	1.031 (0.991-1.073)	0.135	1.053 (1.009-1.098)	0.017	1.071 (0.974-1.172)	0.148
TyG-BMI (Per SD increase)	0.855 (0.818-0.893)	<0.001	0.894 (0.853-0.938)	<0.001	0.708 (0.637-0.783)	<0.001
TyG-WC (Per SD increase)	0.935 (0.896-0.976)	0.002	0.974 (0.929-1.020)	0.265	0.863 (0.783-0.961)	0.005
TyG-WHtR (Per SD increase)	0.967 (0.927-1.009)	0.126	1.009 (0.963-1.058)	0.694	0.933 (0.848-1.034)	0.169
TyG-WWI (Per SD increase)	1.139 (1.092-1.187)	<0.001	1.179 (1.126-1.235)	<0.001	1.352 (1.215-1.492)	<0.001
TyG-ABSI (Per SD increase)	1.188 (1.141-1.237)	<0.001	1.238 (1.186-1.293)	<0.001	1.489 (1.333-1.632)	<0.001
TyG-BRI (Per SD increase)	0.959 (0.918-1.001)	0.055	1.013 (0.967-1.060)	0.590	0.917 (0.830-1.014)	0.090
TyG-CI (Per SD increase)	1.104 (1.059-1.151)	<0.001	1.148 (1.098-1.200)	<0.001	1.263 (1.139-1.397)	<0.001
TyG-RFM (Per SD increase)	0.944 (0.888-1.005)	0.070	1.039 (0.953-1.133)	0.385	0.887 (0.770-1.033)	0.109
**Normal**						
TyG index (Per SD increase)	0.959 (0.880-1.046)	0.345	0.967 (0.888-1.051)	0.428	0.910 (0.737-1.119)	0.377
TyG-BMI (Per SD increase)	0.744 (0.676-0.819)	<0.001	0.787 (0.717-0.864)	<0.001	0.509 (0.399-0.628)	<0.001
TyG-WC (Per SD increase)	0.850 (0.778-0.928)	<0.001	0.858 (0.786-0.936)	<0.001	0.683 (0.534-0.844)	0.001
TyG-WHtR (Per SD increase)	0.884 (0.809-0.966)	0.006	0.908 (0.830-0.992)	0.032	0.748 (0.595-0.941)	0.013
TyG-WWI (Per SD increase)	1.103 (1.009-1.206)	0.031	1.119 (1.013-1.235)	0.027	1.276 (1.005-1.600)	0.040
TyG-ABSI (Per SD increase)	1.190 (1.091-1.299)	<0.001	1.179 (1.070-1.300)	<0.001	1.519 (1.209-1.919)	<0.001
TyG-BRI (Per SD increase)	0.887 (0.812-0.970)	0.008	0.926 (0.846-1.014)	0.097	0.765 (0.617-0.951)	0.015
TyG-CI (Per SD increase)	1.061 (0.972-1.158)	0.186	1.054 (0.961-1.155)	0.266	1.159 (0.913-1.455)	0.215
TyG-RFM (Per SD increase)	0.822 (0.722-0.936)	0.003	1.016 (0.884-1.167)	0.828	0.637 (0.447-0.891)	0.010
**Dysglycemia**						
TyG index (Per SD increase)	0.934 (0.877-0.994)	0.033	0.955 (0.896-1.017)	0.151	0.853 (0.734-0.988)	0.035
TyG-BMI (Per SD increase)	0.836 (0.781-0.894)	<0.001	0.877 (0.812-0.948)	<0.001	0.667 (0.561-0.788)	<0.001
TyG-WC (Per SD increase)	0.895 (0.840-0.954)	<0.001	0.947 (0.883-1.016)	0.128	0.775 (0.661-0.910)	0.002
TyG-WHtR (Per SD increase)	0.938 (0.879-1.000)	0.049	0.993 (0.923-1.069)	0.858	0.860 (0.726-1.011)	0.075
TyG-WWI (Per SD increase)	1.079 (1.011-1.152)	0.022	1.128 (1.049-1.213)	0.001	1.194 (0.998-1.397)	0.039
TyG-ABSI (Per SD increase)	1.113 (1.045-1.186)	<0.001	1.171 (1.093-1.255)	<0.001	1.286 (1.092-1.512)	0.002
TyG-BRI (Per SD increase)	0.956 (0.896-1.020)	0.175	1.014 (0.942-1.091)	0.720	0.904 (0.766-1.056)	0.217
TyG-CI (Per SD increase)	1.032 (0.969-1.100)	0.328	1.078 (1.006-1.155)	0.034	1.076 (0.912-1.259)	0.375
TyG-RFM (Per SD increase)	0.883 (0.800-0.976)	0.015	0.981 (0.835-1.152)	0.814	0.748 (0.571-0.968)	0.031
**Diabetes**						
TyG index (Per SD increase)	1.015 (0.948-1.088)	0.664	1.026 (0.953-1.106)	0.494	1.039 (0.897-1.191)	0.596
TyG-BMI (Per SD increase)	0.790 (0.729-0.857)	<0.001	0.858 (0.786-0.936)	<0.001	0.612 (0.500-0.733)	<0.001
TyG-WC (Per SD increase)	0.875 (0.811-0.945)	<0.001	0.925 (0.851-1.005)	0.065	0.758 (0.622-0.907)	0.004
TyG-WHtR (Per SD increase)	0.893 (0.827-0.964)	0.004	0.938 (0.862-1.021)	0.138	0.788 (0.644-0.947)	0.016
TyG-WWI (Per SD increase)	1.076 (1.002-1.155)	0.044	1.084 (0.999-1.177)	0.054	1.182 (1.005-1.411)	0.054
TyG-ABSI (Per SD increase)	1.141 (1.067-1.221)	<0.001	1.142 (1.063-1.227)	<0.001	1.344 (1.142-1.569)	<0.001
TyG-BRI (Per SD increase)	0.877 (0.810-0.950)	0.001	0.918 (0.844-1.000)	0.050	0.766 (0.622-0.918)	0.007
TyG-CI (Per SD increase)	1.057 (0.986-1.134)	0.120	1.074 (0.995-1.159)	0.069	1.139 (0.971-1.336)	0.110
TyG-RFM (Per SD increase)	0.832 (0.738-0.939)	0.003	0.870 (0.741-1.021)	0.088	0.677 (0.493-0.919)	0.014

**CVD mortality**						
**Total**						
TyG index (Per SD increase)	1.078 (1.005-1.156)	0.036	1.089 (1.010-1.174)	0.027	1.200 (1.015-1.427)	0.035
TyG-BMI (Per SD increase)	0.934 (0.866-1.009)	0.082	0.992 (0.915-1.074)	0.837	0.867 (0.711-1.050)	0.151
TyG-WC (Per SD increase)	1.006 (0.933-1.084)	0.879	1.051 (0.970-1.138)	0.223	1.035 (0.840-1.293)	0.755
TyG-WHtR (Per SD increase)	1.057 (0.980-1.139)	0.152	1.110 (1.025-1.202)	0.010	1.164 (0.951-1.429)	0.145
TyG-WWI (Per SD increase)	1.216 (1.129-1.309)	<0.001	1.263 (1.166-1.368)	<0.001	1.627 (1.364-2.036)	<0.001
TyG-ABSI (Per SD increase)	1.226 (1.142-1.317)	<0.001	1.269 (1.179-1.366)	<0.001	1.655 (1.390-2.017)	<0.001
TyG-BRI (Per SD increase)	1.045 (0.970-1.125)	0.248	1.103 (1.025-1.187)	0.009	1.124 (0.929-1.355)	0.225
TyG-CI (Per SD increase)	1.167 (1.085-1.256)	<0.001	1.203 (1.114-1.298)	<0.001	1.481 (1.241-1.836)	<0.001
TyG-RFM (Per SD increase)	1.073 (0.960-1.200)	0.215	1.235 (1.072-1.422)	0.003	1.234 (0.916-1.684)	0.175
**Normal**						
TyG index (Per SD increase)	0.825 (0.695-0.980)	0.029	0.879 (0.760-1.017)	0.083	0.647 (0.429-0.964)	0.035
TyG-BMI (Per SD increase)	0.724 (0.598-0.878)	<0.001	0.833 (0.706-0.984)	0.031	0.489 (0.281-0.763)	0.005
TyG-WC (Per SD increase)	0.814 (0.682-0.973)	0.023	0.858 (0.722-1.018)	0.080	0.640 (0.371-0.995)	0.076
TyG-WHtR (Per SD increase)	0.868 (0.727-1.036)	0.117	0.955 (0.811-1.124)	0.579	0.741 (0.433-1.151)	0.231
TyG-WWI (Per SD increase)	1.047 (0.877-1.250)	0.610	1.101 (0.930-1.303)	0.264	1.180 (0.691-1.861)	0.512
TyG-ABSI (Per SD increase)	1.097 (0.922-1.306)	0.296	1.080 (0.900-1.296)	0.408	1.307 (0.733-2.092)	0.317
TyG-BRI (Per SD increase)	0.912 (0.766-1.085)	0.297	1.004 (0.850-1.186)	0.961	0.839 (0.505-1.258)	0.450
TyG-CI (Per SD increase)	0.989 (0.830-1.178)	0.899	1.007 (0.853-1.188)	0.938	1.024 (0.593-1.642)	0.927
TyG-RFM (Per SD increase)	0.788 (0.607-1.023)	0.074	1.122 (0.894-1.407)	0.321	0.620 (0.288-1.157)	0.178
**Dysglycemia**						
TyG index (Per SD increase)	0.936 (0.838-1.045)	0.240	0.953 (0.855-1.063)	0.385	0.855 (0.647-1.092)	0.241
TyG-BMI (Per SD increase)	0.893 (0.795-1.004)	0.059	0.917 (0.808-1.040)	0.176	0.770 (0.550-1.000)	0.087
TyG-WC (Per SD increase)	0.936 (0.837-1.047)	0.247	0.987 (0.880-1.107)	0.817	0.856 (0.635-1.117)	0.283
TyG-WHtR (Per SD increase)	1.003 (0.896-1.122)	0.960	1.058 (0.937-1.193)	0.364	1.012 (0.726-1.307)	0.937
TyG-WWI (Per SD increase)	1.120 (1.000-1.255)	0.050	1.198 (1.060-1.355)	0.004	1.331 (0.995-1.748)	0.047
TyG-ABSI (Per SD increase)	1.102 (0.985-1.232)	0.089	1.213 (1.084-1.358)	<0.001	1.279 (0.980-1.669)	0.070
TyG-BRI (Per SD increase)	1.026 (0.918-1.147)	0.647	1.077 (0.957-1.211)	0.217	1.066 (0.789-1.369)	0.648
TyG-CI (Per SD increase)	1.052 (0.941-1.176)	0.372	1.145 (1.016-1.289)	0.026	1.139 (0.863-1.487)	0.348
TyG-RFM (Per SD increase)	0.986 (0.825-1.178)	0.877	1.166 (0.925-1.471)	0.195	0.988 (0.581-1.489)	0.958
**Diabetes**						
TyG index (Per SD increase)	1.081 (0.963-1.213)	0.188	1.080 (0.955-1.222)	0.222	1.214 (0.894-1.563)	0.174
TyG-BMI (Per SD increase)	0.864 (0.755-0.987)	0.032	0.985 (0.854-1.136)	0.834	0.723 (0.501-0.981)	0.058
TyG-WC (Per SD increase)	0.934 (0.820-1.063)	0.298	1.018 (0.892-1.162)	0.793	0.870 (0.609-1.174)	0.406
TyG-WHtR (Per SD increase)	0.958 (0.841-1.090)	0.513	1.065 (0.937-1.210)	0.336	0.922 (0.653-1.251)	0.625
TyG-WWI (Per SD increase)	1.121 (0.995-1.263)	0.061	1.169 (1.029-1.328)	0.016	1.341 (0.990-1.741)	0.042
TyG-ABSI (Per SD increase)	1.166 (1.041-1.307)	0.008	1.154 (1.027-1.296)	0.016	1.472 (1.099-1.885)	0.005
TyG-BRI (Per SD increase)	0.933 (0.816-1.066)	0.306	1.012 (0.889-1.153)	0.854	0.869 (0.611-1.181)	0.405
TyG-CI (Per SD increase)	1.101 (0.978-1.239)	0.112	1.131 (1.002-1.275)	0.046	1.287 (0.936-1.697)	0.097
TyG-RFM (Per SD increase)	0.920 (0.749-1.131)	0.428	1.100 (0.923-1.310)	0.289	0.861 (0.474-1.370)	0.582

Notes:

1. Cox Model 2: Adjusted for age, gender, race, education, PIR, marital status, smoking, alcohol use, antihypertensive drug, hypertension, heart failure, heart disease, angina, heart attack, stroke, failing kidney, and cancer.

2. GPS-Weighted: Generalized propensity score weighted hazard ratios.

3. G-formula: Standardized marginal risk ratios (P90 vs P10 of exposure) with 95% bootstrap CI (500 iterations). ***P***-values calculated from CI using normal approximation on log scale.

Table S11. Life expectancy at age 45 years and years of life lost by tertiles of TyG-derived indices using 10-year age intervals, related to Table 4.

TyG index	Population	T1 (years)	T3 (years)	ΔLE (years)
TyG	Total	33.6	30.2	+3.3
	Normal	33.3	32.9	+0.4
	Dysglycemia	33.8	32.4	+1.4
	Diabetes	28.7	25.1	+3.6
TYG-BMI	Total	32.5	30.7	+1.8
	Normal	32.2	35.1	-2.9
	Dysglycemia	33.7	33.3	+0.5
	Diabetes	27.5	25.3	+2.3
TYG-WC	Total	33.4	29.7	+3.7
	Normal	33.6	33.6	+0.1
	Dysglycemia	34.1	31.2	+2.9
	Diabetes	28.6	24.8	+3.8
TyG-WHtR	Total	33.3	30.1	+3.2
	Normal	33.3	33.5	-0.2
	Dysglycemia	33.9	32.2	+1.7
	Diabetes	28.6	25.4	+3.2
TYG-WWI	Total	34.1	29.2	+4.9
	Normal	35.6	32.5	+3.0
	Dysglycemia	34.2	31.5	+2.7
	Diabetes	29.0	24.3	+4.7
TYG-ABSI	Total	35.2	28.9	+6.2
	Normal	35.8	31.6	+4.2
	Dysglycemia	35.5	31.2	+4.2
	Diabetes	29.6	24.2	+5.4
TYG-CI	Total	34.7	29.1	+5.6
	Normal	34.8	31.7	+3.1
	Dysglycemia	35.1	31.5	+3.6
	Diabetes	29.7	24.2	+5.5
TYG-BRI	Total	33.1	30.2	+2.9
	Normal	33.2	33.2	0.0
	Dysglycemia	34.0	31.8	+2.2
	Diabetes	27.7	25.1	+2.6
TYG-RFM	Total	31.6	32.6	-1.0
	Normal	31.8	34.7	-2.8

Dysglycemia	32.2	35.1	-2.9
Diabetes	27.8	27.7	+0.1

Abbreviations: T1, lowest tertile; T3, highest tertile; ΔLE, years of life lost (T1 − T3); positive values indicate shorter life expectancy in T3 compared with T1; TYG, triglyceride-glucose index; BMI, body mass index; WC, waist circumference; WHtR, waist-to-height ratio; WWI, weight-adjusted waist index; ABSI, a body shape index; CI, conicity index; BRI, body roundness index; RFM, relative fat mass.

Life expectancy at age 45 was calculated using abridged period life tables (10-year age intervals) based on the Chiang method.

Methods S1. Supplemental methodological details for CHARLS cohort selection, TyG-derived index calculations, NHANES survey weighting, and statistical software.

The flowchart (Supplementary Fig. 8) illustrates the participant selection process for the external validation cohort from the China Health and Retirement Longitudinal Study (CHARLS). Of the initial 21,520 participants assessed in the 2011 and 2015 waves, 8,605 were excluded due to: age < 45 years (n = 693), missing follow-up data (n = 1,024), missing triglyceride, fasting blood glucose, or HbA1c data (n = 5,186), indeterminate glucose metabolism status (n = 421), and missing waist, weight, or height data (n = 1,281). The final study population comprised 12,915 participants, stratified by glycemic status into normal glucose metabolism (n = 5,002), dysglycemia (n = 5,706), and diabetes (n = 2,207).

Calculation of the TyG index and its derivative indicators

The triglyceride-glucose (TyG) index and its derivative indices represent composite biomarkers that integrate metabolic and anthropometric parameters to assess cardiometabolic risk. The foundational TyG index combines fasting triglycerides and glucose levels to estimate insulin resistance. We further developed eight derivative indices by incorporating various anthropometric measurements that capture distinct dimensions of body composition and adiposity distribution. These composite indices aim to enhance risk stratification by simultaneously accounting for both metabolic dysfunction and obesity phenotypes. Below, we provide the mathematical formulas for calculating each index.

1. Triglyceride-glucose index (TyG) Formula: $\ln [(TG \times FBG) / 2]$
2. TyG-Body Mass Index (TyG-BMI) Formula: $TyG \times (Weight / Height^2)$
3. TyG-Waist Circumference (TyG-WC) Formula: $TyG \times WC$
4. TyG-Waist-to-Height Ratio (TyG-WHtR) Formula: $TyG \times (WC / Height)$
5. TyG-Weight-Adjusted Waist Index (TyG-WWI) Formula: $TyG \times (WC / \sqrt{Weight})$
6. TyG-A Body Shape Index (TyG-ABSI) Formula: $TyG \times [WC (m) / (BMI^{(2/3)} \times Height (m)^{(1/2)})]$
7. TyG-Conicity Index (TyG-CI) Formula: $TyG \times [WC (m) / (0.109 \times \sqrt{(Weight / Height (m))})]$
8. TyG-Body Roundness Index (TyG-BRI) Formula: $TyG \times (364.2 - 365.5 \times \sqrt{[1 - ((WC / 2\pi)^2 / (0.5 \times Height)^2)]})$
9. TyG-Relative Fat Mass (TyG-RFM) Formula: $TyG \times (64 - 20 \times (Height / WC) + 12 \times Sex)$

NHANES complex survey design and fasting subsample weights

In NHANES, fasting biomarkers (e.g., fasting serum triglycerides and fasting plasma glucose) were measured in the morning fasting subsample. Therefore, analyses involving these biomarkers used the fasting subsample examination weight rather than the standard MEC examination weight. The complex, multistage sampling design was accounted for by incorporating the stratification variable (SDMVSTRA) and primary sampling unit (SDMVPSU).

For analyses combining ten consecutive NHANES cycles (1999–2018), fasting subsample weights were recalculated according to

NCHS analytic guidelines. Specifically, the 4-year fasting weight for 1999–2002 (WTSAF4YR) was divided by five, and each 2-year fasting weight for 2003–2018 (WTSAF2YR) was divided by ten. This rescaling ensured that the combined sample appropriately represented the U.S. civilian noninstitutionalized population over the 1999–2018 study period.

Statistical software and R packages

All statistical analyses in this study were performed using the R programming language (version 4.3.0; R Foundation for Statistical Computing, Vienna, Austria). To meet the complex analytical demands at different stages, we comprehensively utilized multiple specialized R packages. Data preparation and complex sampling design processing during the initial research phase were primarily accomplished using the survey and srvyr packages. The NHANES complex sampling structure was specified using SDMVSTRA and SDMVPSU with appropriate examination weights, and variance estimation was conducted using Taylor series linearization as implemented in the survey package (nest = TRUE).

Survey-weighted Cox proportional hazards models were fitted using svycoxph() to appropriately account for the complex sampling design. Survival analysis formed the core of this study. We employed the survival and survey packages to construct Cox proportional hazards models. Visualization tasks, such as plotting Kaplan-Meier curves, were performed using the survminer package, which also provided an interface for calculating the maximum choice rank statistic to determine optimal cut-off points for continuous variables. For time-dependent ROC analysis, the timeROC package was utilized. For model validation and comparison, the survIDINRI package calculates the Net Receiver Operating Characteristic Improvement Index (NRI) and Integrated Discrimination Improvement Index (IDI). The rmda package performs Decision Curve Analysis (DCA), while the car package assesses multicollinearity (Variance Inflation Factor, VIF). Life expectancy and its cause-specific decomposition were derived from abridged period life tables and an Arriaga-type decomposition implemented with custom R code, with dplyr and tidyr used for data handling and result assembly. For sensitivity analyses and exploratory mechanism-related analyses, we employed multiple advanced methods: The AF package was used to compute population-attributable fractions, the mediation and lavaan packages for mediation effect analysis, the CausalGPS package for generalized propensity score weighting of continuous exposures, and the gfoRmula package for G-formula standardization. Additionally, the boot package provided Bootstrap resampling estimates for key statistics, and the EValue package assessed potential unmeasured confounding bias. Finally, network analysis was implemented using the qgraph and igraph packages, with multiple testing corrected via false discovery rate (FDR) adjustment through the p.adjust function in the base stats package. Dose-response relationships were modeled using restricted cubic splines from the rms package, which also provided relevant modeling functions.
